# Synthesis and properties of 6-alkynyl-5-aryluracils

**DOI:** 10.3762/bjoc.20.80

**Published:** 2024-04-22

**Authors:** Ruben Manuel Figueira de Abreu, Till Brockmann, Alexander Villinger, Peter Ehlers, Peter Langer

**Affiliations:** 1 Universität Rostock, Institut für Chemie, A.-Einstein-Str.3a, 18059 Rostock, Germany; 2 Leibniz Institut für Katalyse an der Universität Rostock, A.-Einstein-Str.29a, 18059 Rostock, Germany

**Keywords:** catalysis, cross-coupling, fluorescence, heterocycles, regioselectivity

## Abstract

The development of a new and straightforward chemoselective method for the synthesis of uracil-based structures by combining Suzuki–Miyaura and Sonogashira–Hagihara cross-coupling is reported. The methodology was applied to synthesize a series of novel compounds. The tolerance of the combination of different functional groups was tested. The influence of different functional groups on the physical properties was studied by ultraviolet–visible (UV–vis) and fluorescence spectroscopy, providing new insights into the potential applications of uracil-based structures.

## Introduction

Organic life is a complex interplay of many different building blocks. One of these building blocks is uracil. Discovered for the first time in 1901 by Alberto Ascoli, it is now known to be one of the four nucleobases of RNA [1]. It therefore plays a very important role in many vital biological processes in the human body and other life forms. Uracil is rarely found in DNA, due to its lower stability and mutagenic properties when mismatched with guanine [2–5]. This fact can be used to differentiate between RNA and DNA-dependent targets, making uracil very interesting for medical applications. The first modification of uracil was already synthesized in 1906 [6]. However, the medical potential of uracil was not discovered until 40 years later. One of the first antibacterial studies was carried out in 1945. The first anticarcinogenic studies followed in 1953 [7–8]. Since then, uracil has played an important role in the development of antiviral and anticarcinogenic agents against various targets [9–16].

**Figure 1:**
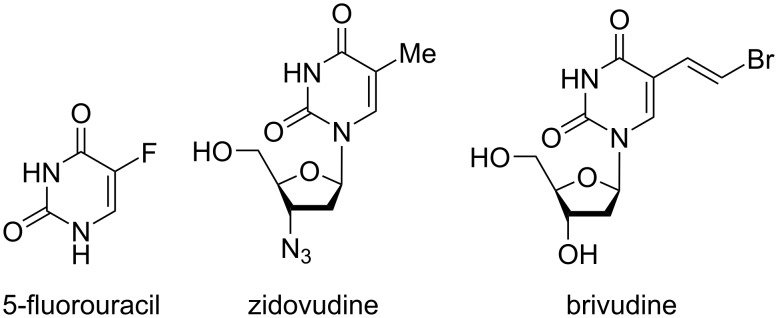
Uracil derivatives in drugs.

5-Fluorouracil is one of the best-known anticancer drugs and is used to treat a variety of cancers, including pancreatic, breast, and cervical cancers. Zidovudine is effective against retroviruses and is still used today for the prevention and treatment of HIV/AIDS infections. Brivudine is one of the most potent antiviral agents against herpes zoster virus infections (Figure 1) [17–20].

**Figure 2:**
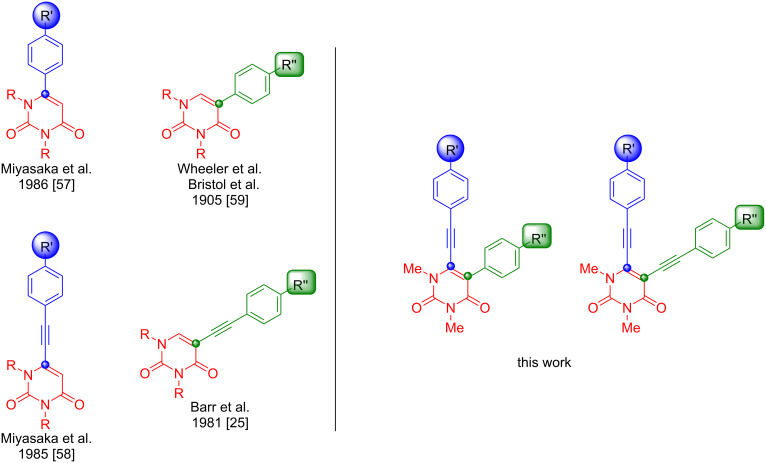
Present work as compared to previous studies [25,57–59].

Given the proven medical potential of uracil, further investigation was carried out to fully utilize the synthetic possibilities uracil has to offer and to synthesize new drugs against existing or hitherto unknown targets. Moreover, studies have even shown that a sugar moiety is not always required to act against a targeted enzyme [21–23]. One of these focus areas was the synthesis of alkyne-linked derivatives. The first alkyne-linked compound was already published in 1976, accompanied by new synthesis methods in the following years [24–27]. With the discovery of potential antiviral properties and other synthetic opportunities, alkyne-linked derivatives remained an integral part of research to the present day [28–35]. However, the main methods known so far are to substitute uracil either only at position 6 or at position 5 [26–28,34–47]. The remaining known methods use only both positions to induce cyclization [25,31–33,48–56]. Therefore, to the best of our knowledge, there are no known methods that allow the selective reaction of both positions of uracil (Figure 2).

In this work, we report a new chemoselective method for the synthesis of a series of hitherto unknown uracil-based compounds by combining Suzuki–Miyaura and Sonogashira–Hagihara cross-coupling [60–61]. The method is designed to be flexible and could also be used to synthesize other structural motifs. Applications and tolerance to a wide range of functional groups have been tested. Furthermore, their physical properties were analyzed by ultraviolet–visible and fluorescence spectroscopy.

## Results and Discussion

### Synthesis

**Scheme 1:**
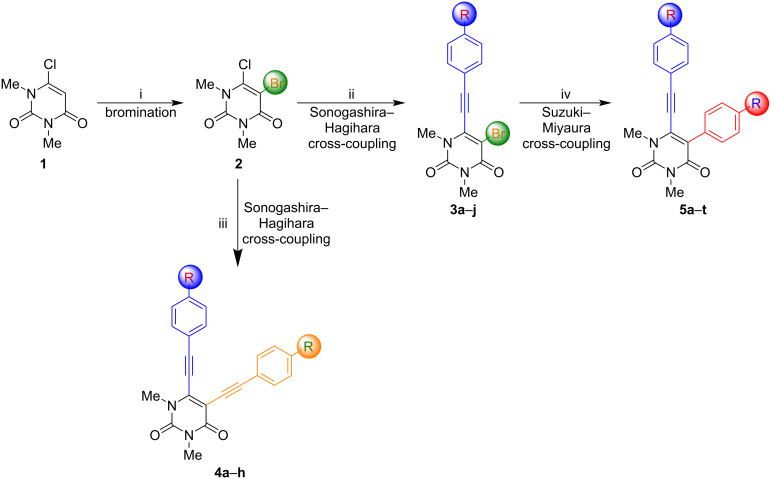
Synthesis of 1,3-dimethly-5-[2-(aryl)ethynyl]-6-[2-(aryl)ethynyl]uracils **4** and 1,3-dimethyl-5-aryl-6-[2-(aryl)ethynyl]uracils **5**. Conditions: i) Br_2_ (1.0 equiv), Ac_2_O (1.5 equiv), AcOH, 25 °C, 1 h [62]; ii) Pd(PPh_3_)Cl_2_ (5 mol %), CuI (5 mol %), NEt_3_ (10 equiv), DMSO, 25 °C, 6 h; iii) Pd(CH_3_CN)_2_Cl_2_ (5 mol %), CuI (5 mol %), NEt_3_ (10 equiv), 1,4-dioxane, 100 °C, 6 h; iv) Pd(PPh_3_)_4_ (10 mol %), NaOH (3 equiv), 1,4-dioxane/water 5:1, 100 °C, 1 h.

The synthetic strategy for the desired compounds is based on a four-step sequence starting with commercially available 6-chloro-1,2-dimethyluracil (**1**), as depicted in Scheme 1. Subsequently, 5-bromo-6-chloro-1,3-dimethyluracil (**2**) was synthesized by brominating the starting material. The single Sonogashira–Hagihara cross-coupling afforded **3a**–**j** and, by a two-fold approach, **4a**–**h** could be obtained. Compounds **3a**–**j** were subsequently transformed to **5a**–**t** by Suzuki–Miyaura cross-coupling.

**Scheme 2:**
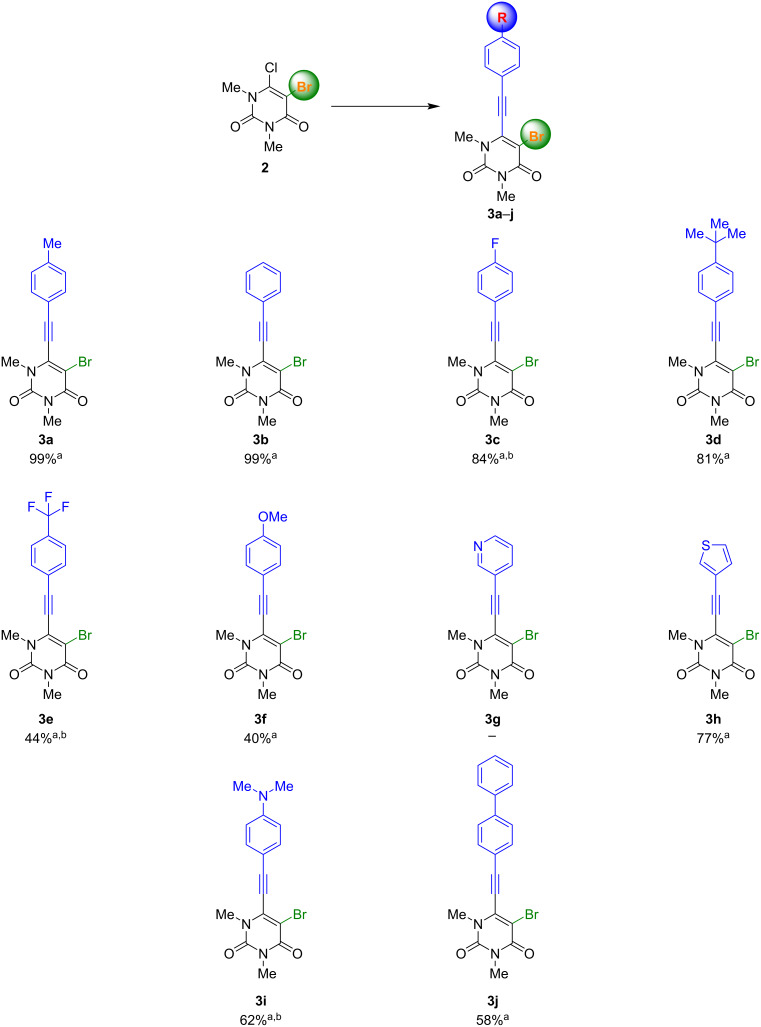
Synthesis of 5-bromo-1,3-dimethyl-6-[2-(aryl)ethynyl]uracils **3a**–**j**. Reaction conditions: **2** (1.0 equiv), arylacetylene (1.2 equiv), Pd(PPh_3_)Cl_2_ (5 mol %), CuI (5 mol %), NEt_3_ (10 equiv), DMSO, 25 °C, 6 h. ^a^Yields of isolated products. ^b^Reaction temperature: 50 °C.

The bromination of **1** was performed by using Br_2_ (1 equiv), Ac_2_O (1.5 equiv), AcOH (25 °C, 1 h) and yielded the desired product in 52% [62]. With the starting material in hand, initial Sonogashira–Hagihara cross-coupling was carried out using Pd(PPh_3_)_4_ as catalyst with K_3_PO_4_ as base in toluene as solvent which gave a mixture of different products. Further investigation revealed the presence of the two-fold Sonogashira–Hagihara product, starting material **2**, and the desired product **3**. Hence, starting material **2** and product **3** show similar reactivity under the employed reaction conditions. Therefore, the reaction had to be optimized to overcome this competition and to achieve a higher yield and selectivity for the desired product (Supporting Information File 1, Table S1). Finally, the transformation was realized by using Pd(PPh_3_)Cl_2_ (5 mol %), CuI (5 mol %), NEt_3_ (10 equiv), DMSO, at 25 °C for 6 h as reaction conditions with excellent yield and selectivity. With the optimized conditions in hand, the next step was to investigate the scope and afford **3a**–**j**. The corresponding results are depicted in Scheme 2.

**Scheme 3:**
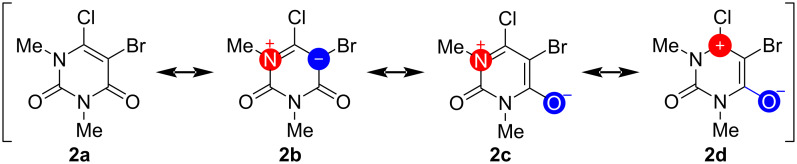
Structure of the starting material **2** with its possible mesomeric structures.

The scope resulted in the synthesis of products **3a**–**j** with very good to good yields and was performed with a high tolerance towards different functional groups, allowing for a wide range of applications. However, lower yields were observed when a strong electron-withdrawing or pushing group was used. This effect can be observed for **3e**, **3f** and **3i**. Furthermore, the separation of these products has proven to be more challenging than other compounds with higher yields. In the reaction with 3-pyridylacetylene no product **3g** could be obtained. The reaction at 50 °C was found to be chemoselective, giving only the 6-substituted product. This behavior has also been observed in previous studies [63]. It was expected that there would be a low chemoselectivity, due to the availability of two halogenated positions in the starting material. However, the 5-substituted product was not observed. At higher temperatures, only the double-substituted product could be found. No reaction was observed when the reaction temperature was lowered to 0 °C. This could be due to the double activation of the 5-position, despite the fact that bromine is a better leaving group than chlorine. Both positions might be influenced by the functional groups adjacent to them, due to withdrawing effects. Chlorine has a stronger electron-withdrawing effect than bromine, so the electrophilic character of the 6-position should be higher than that of the 5-position. However, it is unlikely to be the only reason for the formation of just one intermediate. A second effect appears to play a more important role and could be related to the structure of the starting material. The 6-position is part of an enamine and an α,β-unsaturated carbonyl structure, as depicted in Scheme 3. According to the mesomeric structure of the enamine, the 6-position could be activated, and the 5-position deactivated for the nucleophilic attack that occurs during the oxidative addition of the metal catalyst. This may explain the formation of only the 6-substituted product during the Sonogashira reaction.

**Scheme 4:**
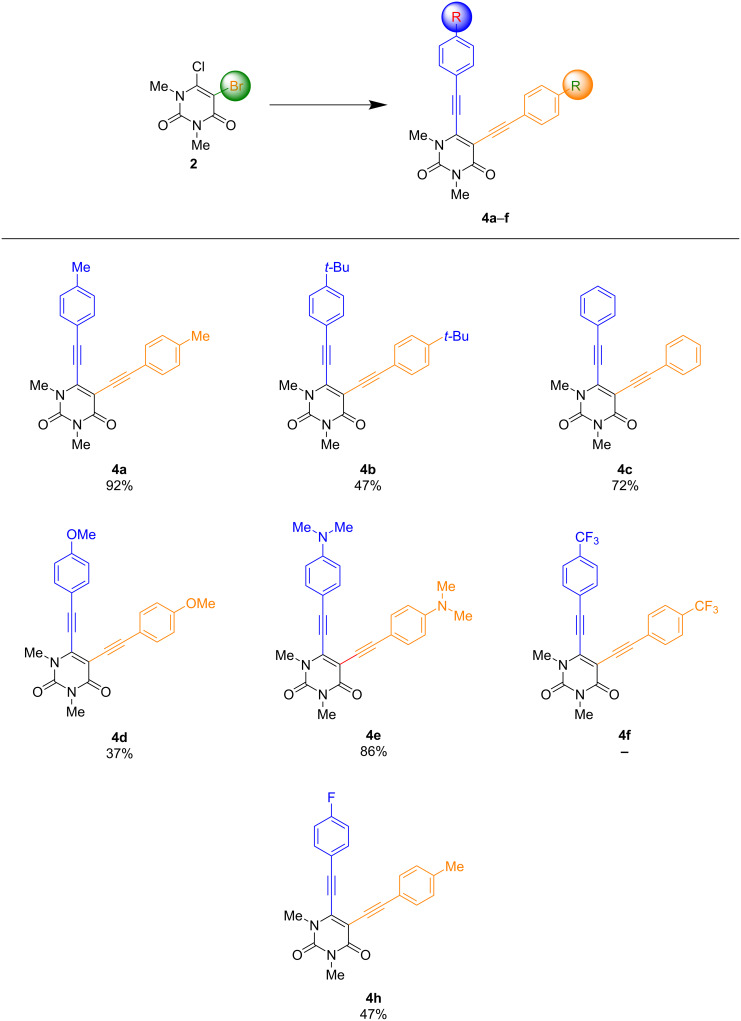
Synthesis of 1,3-dimethyl-5-[2-(aryl)ethynyl]-6-[2-(aryl)ethynyl]uracils **4a**–**h**. Reaction conditions: **2** (1 equiv), arylacetylene (2.2 equiv), Pd(CH_3_CN)_2_Cl_2_ (5 mol %), CuI (5 mol %), NEt_3_ (10 equiv), dioxane, 100 °C, 6 h. Yields of isolated products.

As mentioned above, new reaction conditions had to be chosen to synthesize the desired product **4** and to avoid a mixture. A different catalyst and a higher temperature were chosen to obtain the desired products in higher yields. With the optimized conditions in hand (Pd(CH_3_CN)_2_Cl_2_ (5 mol %), CuI (5 mol %), NEt_3_ (10 equiv), dioxane, 100 °C, 6 h), the scope was investigated next and allowed for the synthesis of compounds **4a**–**h** (Scheme 4).

The formation of compounds **4a**–**h** was achieved over two steps with very good to good yields and a high tolerance towards different functional groups was observed. Similarly to the monoalkynylated products, lower yields were obtained with stronger electron-withdrawing groups, as can be seen in case of product **4d**. The yield of **4b** was comparatively lower, which may be due to the higher steric hindrance of the used arylacetylene. Product **4f** could not be synthesized, due to decomposition during the reaction.

Furthermore, no precursor could be isolated. This could be due to instabilities caused by two strong electron-withdrawing groups. As a prove of concept, a sequential reaction set-up was used to realize the formation of product **4h** containing differentially functionalized arylalkynes.

Subsequently, the formation of the desired product **5** was carried out by application of the Suzuki–Miyaura cross-coupling. The optimization was carried out with **3a** as the model compound (Table 1).

**Table 1:** Optimization of the reaction conditions for the synthesis of **5a**.

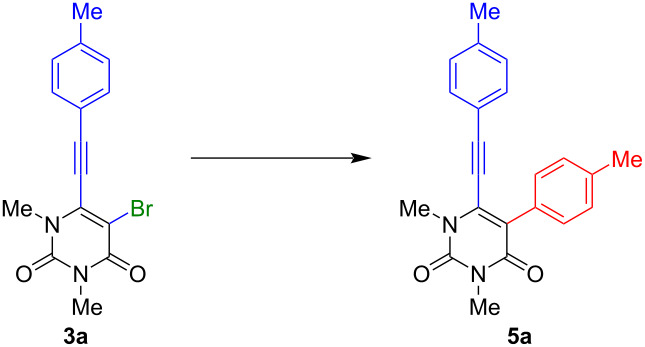								

Entry	Boronic acid (equiv)	Cat. (mol %)	Ligand (mol %)	Base (equiv)	Solvent (mixture)	Temp (°C)	Time (h)	Yield (%)

1	1.2	Pd(CH_3_CN)_2_Cl_2_ (5)	SPhos (10)	Na_2_CO_3_ (3)	toluene	100	16	27
2	1.2	Pd(CH_3_CN)_2_Cl_2_ (5)	SPhos (10)	Na_2_CO_3_ (3)	DMA	150	16	–
3	2.5	Pd(CH_3_CN)_2_Cl_2_ (5)	SPhos (10)	Na_2_CO_3_ (3)	toluene	100	16	34
4	1.2	Pd(PPh_3_)_2_Cl_2_ (5)	SPhos (10)	Na_2_CO_3_ (3)	toluene	100	16	–
5	1.2	Pd(PPh_3_)_4_ (5)	SPhos (10)	Na_2_CO_3_ (3)	toluene	100	16	31
6	1.2	Pd(PPh_3_)_4_ (10)	–	Na_2_CO_3_ (3)	toluene	100	16	27
7	1.2	Pd(PPh_3_)_4_ (10)	–	K_3_PO_4_ (3)	toluene	100	16	24
8	1.2	Pd(PPh_3_)_4_ (10)	–	KO*t*-Bu (3)	toluene	100	16	–
9	1.2	Pd(PPh_3_)_4_ (10)	–	KO*t*-Bu (3)	dioxane	100	16	–
10	1.2	Pd(PPh_3_)_4_ (10)	–	NaOH (3)	dioxane	100	16	44
11	1.2	Pd(PPh_3_)_4_ (10)	–	NaOH (3)	dioxane/water 5:1	100	16	55
12	1.2	Pd(PPh_3_)_4_ (10)	–	NaOH (3)	dioxane/water 5:1	100	6	53
13	1.2	Pd(PPh_3_)_4_ (10)	–	NaOH (3)	dioxane/water 5:1	100	1	62

The starting material **3a** is a sterically hindered system in which the bromine is only partially accessible, due to the large residue. This could be the reason for the low yield of the first approach. Replacing the catalyst and increasing the temperature or the amount of boronic acid proved to be unsuccessful. With entry 6 (Table 1) it was shown that similar yields could be obtained by removing the ligand and using higher amounts of catalyst. Therefore, no additional ligand was used in the next attempts.

**Scheme 5:**
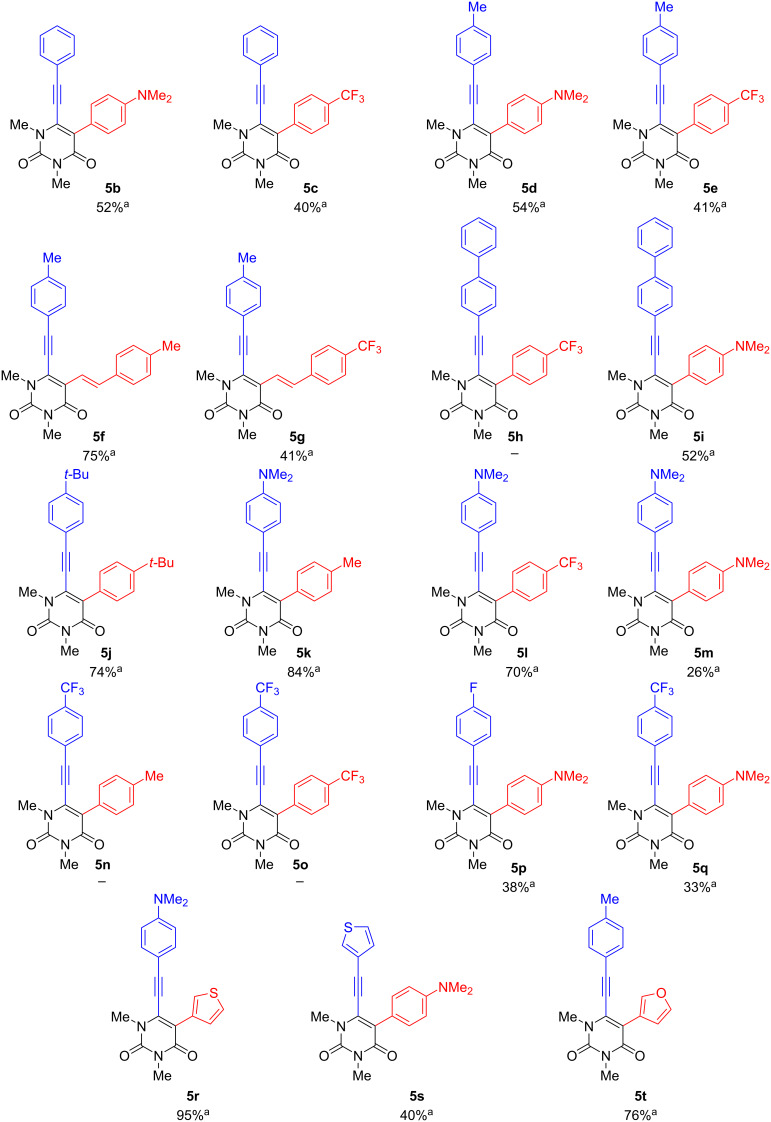
Synthesis of 1,3-dimethyl-5-aryl-6-[2-(aryl)ethynyl]uracils **5a**–**t**. Reaction conditions: **3** (1 equiv), boronic acid (1.2 equiv), Pd(PPh_3_)_4_ (5 mol %), NaOH (3 equiv), dioxane/water 5:1, 100 °C, 1 h. ^a^Yields of isolated products.

Additionally, it was observed that by replacing the less polar solvent by a mixture of a more polar solvent and water in a 5:1 ratio, yields could be significantly improved. Therefore, sodium hydroxide was used as the water-soluble base, which also proved to be beneficial to the yield. Finally, by monitoring the reaction time, it was discovered that reducing the reaction time to 1 h further improved the yield to 62%. Finally, using the optimized conditions (Table 1, entry13), the scope was carried out and afforded the desired products **5a**–**t** (Scheme 5).

The products **5a**–**t** were obtained in 95 to 25% yields, with an average yield of 56%. Furthermore, the method was shown to be highly tolerant towards different functional groups and their combinations. A higher yield tended to be observed when a donor group was located on the arylalkyne at the 6-position (**5k**, **5l, 5r**). However, this effect seems to be neutralized when using an electron-rich arylboronic acid (**5m**). Lower yields are obtained when an acceptor group is present on the arylalkyne (**5p**, **5q**). This leads to the suggestion that an electron-donor group activates and an electron-withdrawing group deactivates the compound for the subsequent Suzuki reaction. Furthermore, lower yields were generally observed when a strong electron-donor or -acceptor was attached to the phenyl group at position 5. Higher yields could be obtained by reducing the steric hindrance at position 5 by introducing a 5-membered ring instead of a benzene moiety. This was demonstrated by the introduction of thiophene (**5r**) and furan (**5t**) to the uracil structure. The molecules **5n** and **5o** could not be obtained, due to decomposition during the reaction.

**Figure 3:**
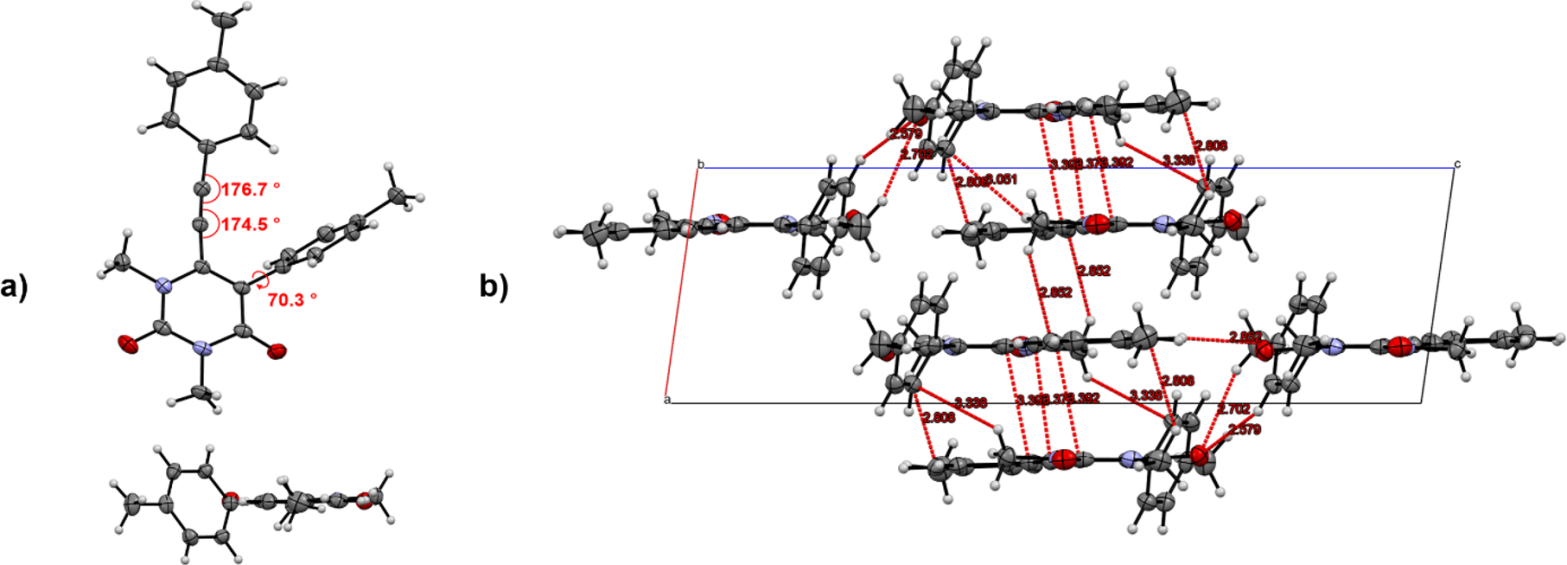
ORTEP diagram of **5a** front view (a) and side view (b). a) Interactions of the molecules within and between a layer with two molecules at the top and the bottom of the unit cell. b) Determined from X-ray structural analysis at 123 K. Element color: carbon (grey), hydrogen (white), oxygen (red) and nitrogen (blue). The thermal ellipsoids are drawn at the 50% probability level.

The structure of **5a** was confirmed by X-ray crystallographic analysis. Crystals were obtained by slow evaporation of the solvent from a mixture of the compound in dichloromethane and heptane at room temperature (Figure 3).

Crystal structure analysis revealed that **5a** crystallizes in a base-centered monoclinic system with the *P*21/*c* space group. The structure is mostly planar, except for the 5-phenyl group, which is twisted out of the plane with a dihedral angle of φ = 70.3°. Furthermore, it could be observed that the 6-[2-(phenyl)ethynyl] group is slightly curved, due to the dihedral angle of the alkyne group (174.5 ° and 176.7 °).

Analysis of the lattice structure of **5a** revealed that the distance between the molecules within the unit cell is higher than the distance between the unit cells. In addition, the molecules are arranged parallel-displaced to each other, which is considered to be more stable than the sandwich arrangement [64–68]. Consequently, the distance between the layers varies periodically between 3.373 Å and 3.662 Å. This results in different interactions between the layers, as depicted in Figure 3. Furthermore, the layers are arranged in an anti-parallel face-to-face order to reduce the steric hindrance. Within the shorter distance layers (3.373 Å), π–π interactions between the alkyne groups can be observed. Moreover, the alkyne phenyl group interacts mainly with molecules between different layers in the vertical direction, while the phenyl group interacts vertically and horizontally with different molecules.

The two-fold Suzuki reaction was also investigated, but no desired product could be obtained. In fact, starting material **2** appears to be too unreactive to undergo a Suzuki reaction. Reversing the reaction steps gave the desired intermediate 5-bromo-6-phenyl-1,3-dimehtyluracil, which turned out to be unstable. This could be the reason why the desired product could not be synthesized at the first attempt. Therefore, the synthesis of the desired product from starting material **2** appears to be unlikely. It is reasonable to assume that this finding is due to the instability of the intermediate formed, as the desired product has been synthesized by other methods and the single Suzuki reaction on uracil is well studied [42,69–71].

### Physical properties

**Figure 4:**
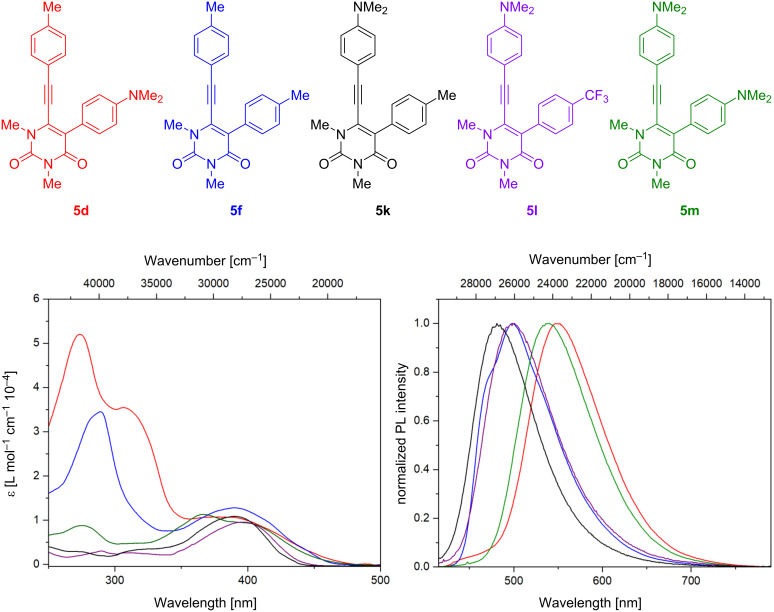
UV–vis absorption (left) and emission (right, λ_ex_ = 400 nm) spectra of 1,3-dimethyl-5-phenyl-6-[2-(phenyl)ethynyl]uracil derivatives **5d**, **5f**, **5k**, **5l**, and **5m** in dichloromethane (*c* = 1·10^−5^ M).

The photophysical properties of selected derivatives were investigated by steady-state absorption and photoluminescence spectroscopy. The influence of the substitution pattern on the photophysical properties is displayed in Figure 4. Corresponding photophysical data and quantum yields are described in Table 2.

**Table 2:** Photophysical data of selected 1,3-dimethyl-5-phenyl-6-[2-(phenyl)ethynyl]uracil derivatives **5d**, **5f**, **5k**, **5l**, and **5m** in dichloromethane (*c* = 1·10^−5^ M) at 20 °C.

	**5d**	**5f**	**5k**	**5l**	**5m**

λ_1,abs_ (nm) ε_λ1_ (M^−1^ cm^−1^)	273 52015	289 35001	324 3470	289 3008	274 8766
λ_2,abs_ (nm) ε_λ2_ (M^−1^ cm^−1^)	306 35462	389 13267	390 10846	396 9548	368 11283
λ_3,abs_ (nm) ε_λ3_ (M^−1^ cm^−1^)	387 10644				402 9259
λ_1,em_^400^ (nm) λ_2,em_^400^ (nm)	548	473^a^ 497	480	498	537
Φ^b,c^	13%	23%	2%	9%	

^a^Shoulder in the spectrum. ^b^For the excitation wavelength λ_ex_ = 400 nm. ^c^Fluorescence standards: coumarin153 in ethanol (Φ = 0.38) [72–73].

The analysis of the absorption spectra revealed that the spectra can be divided into a short-wave and long-wave region. In the long wavelength region (350–500 nm) all measured compounds show a very similar absorption band, indicating that the first transition state (S_0_ → S_1_) does not seem to be affected by the substitution pattern. Furthermore, all compounds show broadened absorption bands and no major differences in the first absorption band were observed. The greatest difference was observed in the short wavelength region (250–350 nm). Interestingly, compounds with a π-donor group on the 5-phenyl group were found to have the highest absorption intensity (**5d**, **5f**, and **5m**) in the short wavelength region. Particularly noteworthy is compound **5d**, where the highest intensity could be observed with an absorption band at 273 nm and a shoulder at 306 nm. The second highest peak was observed for **5f**, followed by **5m**. Only one broadened peak was observed in both cases.

The influence of the substitution pattern could be revealed by comparing the two regioisomers **5d** and **5k**. As a result, the overall absorption intensity in the short-wavelength region was drastically reduced. This leads to the assumption that this absorption band is highly influenced by the dimethylamino group on the 5-phenyl group, even though the phenyl ring is twisted out of the plane. Interaction between the two π-systems is rather unlikely due to the twisted position of the phenyl ring. It is therefore reasonable to assume that this behavior is more likely to be related to a possible interaction of the dimethylamino group with the rest of the system.

However, the combination of two dimethylamino groups appears to have the opposite effect, as **5m** demonstrates. Although it was expected that this would result in a higher absorption intensity, a drastic reduction in the intensity was observed. The extinction coefficient of **5m** (8766 M^−1^ cm^−1^) was rather low compared to **5d**. Furthermore, the substitution of a π-donor group by a π-acceptor group led to a reduction of the absorption intensity until it almost disappeared. This behavior can be observed in the spectra of **5l**. As the spectra of **5k** and **5l** indicate, the spectral influence of a *p*-substituted alkyne-linked phenyl group appears to be negligible. However, this negligible influence could also be explained by the distance between the functional group and the core system.

Compound **5f** is extended by a double bond between the uracil entity and the phenyl group. In addition, it is not influenced by groups other than methyl. Interestingly, it has the second-highest overall absorption intensity after **5d** and a similar absorption peak can be observed. The similarity could be due to the double bond and its ability to interact with the system as a π-donor group. Consequently, the double bond could similarly influence the system as the dimethylamino group. As already discussed for **5d**, this again reinforces the assumption that this phenomenon could be caused by a π-donor at 5-position, regardless of its structure. Further investigation is required, but if confirmed, this could be used to create specific desired absorption behaviors.

Subsequently, the emission spectra were investigated. As explained in the previous section, a similar behavior was observed in the emission spectra with respect to the substitution pattern. In general, a bathochromic shift of the emission was observed in the presence of a dimethylamino group at the 5-position. The bathochromic shift of **5d** compared to its regioisomer **5k** is 68 nm. This underlines the spectral effect of the π-donor on the 5- or 6-position and the dependence of the distance between the functional group and the uracil core system.

In the spectra of **5m**, a combination of both situations can be observed. The bathochromic shift of **5m** is 57 nm and lies between the two emission peaks of **5d** and **5k**. Therefore, it can be concluded that combining the two substitution patterns leads to this intermediate behavior. Substitution of the 5-π-donor with a π-acceptor group reduced the bathochromic shift from 57 nm to 18 nm, which may indicate that the influence of the π-acceptor group is higher than that of a methyl group, but much weaker than that of a π-donor group (**5l**). Similar findings were also observed in the absorption behavior.

In the emission spectra of **5f**, no significant difference regarding the shift could be observed, despite the presence of the π-donor group at the same position. However, a shoulder on the emission peak was observed, which may indicate the influence of the double bond.

Finally, the quantum yields of all compounds were determined and compared. Compound **5f** afforded the highest quantum yield with 23% and **5k** the lowest with 2%. The second highest quantum yield was obtained with **5d** closely followed by **5m**. With regard to the previously discussed data, this could also be due to the influence of a π-donor group directly connected to the core system. This again shows the influence of the substitution pattern on the phenyl groups and that the properties can be modulated by the choice of substituents.

## Conclusion

In summary, we have developed a new, straightforward method for the synthesis of a series of new and hitherto unknown uracil derivatives. Different structural motifs could be obtained based on the same starting material. Furthermore, we could demonstrate a high tolerance towards different functional groups and their combinations. The physical properties of selected derivatives were investigated by steady-state absorption and photoluminescence spectroscopy. The corresponding data gives first insights into the optical properties. It was observed that the photophysical properties could be partially modulated by the chosen substituents.

## Experimental

### General information

Nuclear magnetic resonance spectra (^1^H/^13^C/^19^F NMR) were recorded on a Bruker AVANCE 300 III, 250II, or 500. The analyzed chemical shifts δ are referenced to the residual solvent signals of the deuterated solvents CDCl_3_ (δ = 7.26 ppm/77.16 ppm). Multiplicities due to spin–spin correlation are reported as follows: s = singlet, d = doublet, dd = double doublet, m = multiplet; they are further described by their coupling constants *J*. Infrared spectra (IR) were measured as attenuated total reflection (ATR) experiments using a Nicolet 380 FT-IR spectrometer. The signals were characterized by their wavenumbers and corresponding absorption as very strong (vs), strong (s), medium (m), weak (w) or very weak (vw). UV–vis spectra were recorded on a Cary 60 UV–vis spectrophotometer and emission spectra were recorded on an Agilent Cary Eclipse fluorescence spectrophotometer. Basic and high-resolution mass spectra (MS/HRMS) were measured on instruments coupled to a preceding gas chromatograph (GC) or liquid chromatograph (LC). Samples were ionized by electron impact ionization (EI) on an Agilent 6890/5973 or Agilent 7890/5977 GC–MS equipped with a HP-5 capillary column using helium carrier gas or by electron spray ionization (ESI) on an Agilent 1200/6210 time-of-flight (TOF) LC–MS. X-ray single-crystal structure analysis was performed on a Bruker Apex Kappa-II CCD diffractometer. The solvents used, dimethyl sulfoxide and 1,4-dioxane, were purchased as dry solvents and applied without further purification. Other reagents, catalysts, ligands, acids, and bases were used as purchased from commercial suppliers. Column chromatography was performed on Merck Silica gel 60 (particle size 63–200 μm). Solvents for extraction and column chromatography were distilled prior employment.

### Representative method for the preparation of starting materials

**5-Bromo-6-chloro-1,3-dimethyluracil (2)**. Uracil **1** (22.9 mmol; 4.00 g) was dissolved in glacial acetic acid (60 mL) and after 5 min acetic anhydride (3 mL) was added. The reaction mixture was stirred for 10 min. Then, bromine (1 equiv; 23.4 mmol; 1.2 mL) was slowly added dropwise. After 1 hour, the reaction was stopped by adding water (25 mL) and cooling to 4 °C for 30 minutes. The precipitate was then filtered and dried.

**Representative procedure A for the synthesis of 3a–j**. A mixture of **2** (1.99 mmol; 0.504 mg), Pd(PPh_3_)Cl_2_ (5 mol %; 98.3 µmol; 6.9 mg), CuI (5 mol %; 98.7 µmol; 18.8 mg) was dissolved in DMSO (5 mL) and stirred for 5 min under an argon atmosphere. NEt_3_ (11 equiv; 21.5 mmol; 3 mL) was added and the emulsion was stirred for 10 min at room temperature. The corresponding arylacetylene (*p*-tolylacetylene, 2.20 mmol, 0.28 mL) was slowly added dropwise to the reaction mixture and stirred for 6 hours at room temperature. The reaction was monitored by TLC until the reaction was complete. The reaction was neutralized with an HCl solution (1 M) and diluted with water (40 mL). The phases were separated, and the aqueous layer was extracted with dichloromethane (3 × 30 mL). The combined organic layers were dried over Na_2_SO_4_, concentrated under reduced pressure, and purified by column chromatography (heptane/ethyl acetate).

**Representative procedure B for the synthesis of 4a–j**. A mixture of **2** (402 µmol; 102 mg), Pd(CH_3_CN)_2_Cl_2_ (5 mol %; 23 µmol; 6 mg), CuI (5 mol %; 21 µmol; 4 mg) was dissolved in 1,4-dioxane (5 mL) and stirred for 5 min under an argon atmosphere. NEt_3_ (11 equiv; 4.30 mmol; 0.6 mL) was added and the reaction mixture was heated to 100 °C. The corresponding arylacetylene (*p*-tolylacetylene, 2.2 equiv; 946 µmol; 0.12 mL) was slowly added dropwise to the reaction mixture and stirred for 6 hours at 100 °C. The reaction was monitored by TLC until the reaction was complete. The reaction was neutralized with an HCl solution (1 M) and diluted with water (40 mL). The phases were separated, and the aqueous layer was extracted with dichloromethane (3 × 30 mL). The combined organic layers were dried over Na_2_SO_4_, concentrated under reduced pressure, and purified by column chromatography (heptane/ethyl acetate).

**Representative procedure C for the synthesis of 4h**. A mixture of **2** (400 µmol; 100 mg), Pd(CH_3_CN)_2_Cl_2_ (5 mol %; 23 µmol; 6 mg), CuI (5 mol %; 21 µmol; 4 mg) was dissolved in 1,4-dioxane (5 mL) and stirred for 5 min under an argon atmosphere. NEt_3_ (11 equiv; 4.30 mmol; 0.6 mL) was added and the reaction mixture was heated to 50 °C. The corresponding arylacetylene (*p*-tolylacetylene, 1.1 equiv; 473 µmol; 0.06 mL) was slowly added dropwise to the reaction mixture and stirred for 6 hours at 50 °C. After 6 hours, the second corresponding arylacetylene (4-fluorophenylacetylene, 1.1 equiv; 460 µmol; 0.06 mL) was slowly added dropwise to the reaction mixture and heated at 100 °C. The reaction was stirred at 100 °C for a further 6 h. The reaction was monitored by TLC until the reaction was complete. The reaction was neutralized with an HCl solution (1 M) and diluted with water (40 mL). The phases were separated, and the aqueous layer was extracted with dichloromethane (3 × 30 mL). The combined organic layers were dried over Na_2_SO_4_, concentrated under reduced pressure, and purified by column chromatography (heptane/ethyl acetate).

**Representative procedure D for the synthesis of 5a–t**. A mixture of **3a** (303 µmol; 109 mg), Pd(PPh_3_)_4_ (10 mol %; 369 µmol; 5 mg), NaOH (3.0 equiv; 933 µmol; 37 mg) and the corresponding boronic acid (4-tolylboronic acid, 1.2 equiv; 369 µmol; 5 mg) was dissolved in a mixture of 1,4-dioxane and water 5:1. The reaction mixture was heated to 100 °C and stirred for 1 hour. The reaction was monitored by TLC until the reaction was complete. The reaction was neutralized with an HCl solution (1 M) and diluted with water (40 mL). The phases were separated, and the aqueous layer was extracted with dichloromethane (3 × 30 mL). The combined organic layers were dried over Na_2_SO_4_, concentrated under reduced pressure, and purified by column chromatography (heptane/ethyl acetate).

## Supporting Information

File 1 Experimental data and copies of spectra.

## Data Availability

All data that supports the findings of this study is available in the published article and/or the supporting information to this article.
