# Supplementary material for: Synthesis and properties of 6-alkynyl-5-aryluracils
Source: Beilstein J Org Chem. 2024 Apr 22;20:898–911. doi: 10.3762/bjoc.20.80 (PMC11070962; doi:10.3762/bjoc.20.80)

BEILSTEIN JOURNAL OF ORGANIC CHEMISTRY

Supporting Information

for

Synthesis and properties of 6-alkynyl-5-aryluracils

Ruben Manuel Figueira de Abreu, Till Brockmann, Alexander Villinger, Peter Ehlers and Peter Langer

Beilstein J. Org. Chem. **2024,** *20,* 898–911. doi:10.3762/bjoc.20.80

Experimental data and copies of spectra

License and Terms: This is a supporting information file under the terms of the Creative Commons Attribution License (https://creativecommons.org/licenses/by/4.0). Please note that the reuse, redistribution and reproduction in particular requires that the author(s) and source are credited and that individual graphics may be subject to special legal provisions.
The license is subject to the *Beilstein Journal of Organic Chemistry* terms and conditions: (https://www.beilstein-journals.org/bjoc/terms)

Table of contents

Single-crystal X-ray diffraction data S1

Optimisation: Synthesis of **3a**.. S2

Experimental data S3

^{1}H, ^{13}C, and ^{19}F NMR spectra.. S14

Single-crystal X-ray diffraction data

a)

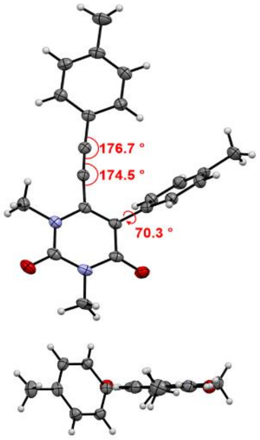

b)

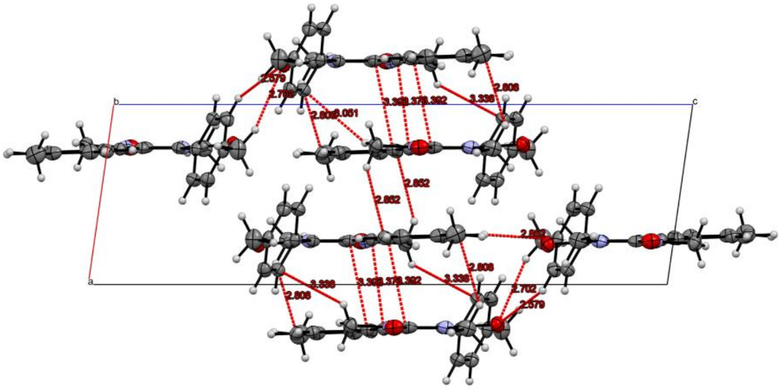

Figure 1: ORTEP diagram of **5a** - front view (top) and side view (bottom) (a). Interactions of the molecules within and between a layer with two molecules at the top and the bottom of the unit cell (b). Determined from X-ray structural analysis at 123 K. Element colour: carbon (grey), hydrogen (white), oxygen (red) and nitrogen (blue). The thermal ellipsoids are drawn at the 50 % probability level.

	**5a**
Chem. Formula	$C_{22}H_{20}N_2O_2$
Form. Weight [g mol−1]	344.40
Cryst. system	monoclinic
Cryt. descript	block
Space group<br>(Hall group)	P 21/c<br>-P 2ybc
Color	colourless
a [Å]	6.7559(3)
b [Å]	12.3613(5)
c [Å]	21.5403(10)
α [°]	90
β [°]	98.074
γ [°]	90
V [Å^3]	1781.04(14)
Z	4
N_{ref}	4292
θ [°]	28.000
h,k,l_{max}	8,16,28
Dx [g cm^{-3}]	1.284
μ [mm^{-1}]	0.083
$\lambda_{MoK\backslash\alpha}$ [Å]	0.71073
T [K]	123
F(000)	728.0
Npar	240
R	0.0470(3634)
w*R*	0.1406(4292)
S	1.080

Table S1: Synthesis of **3a**

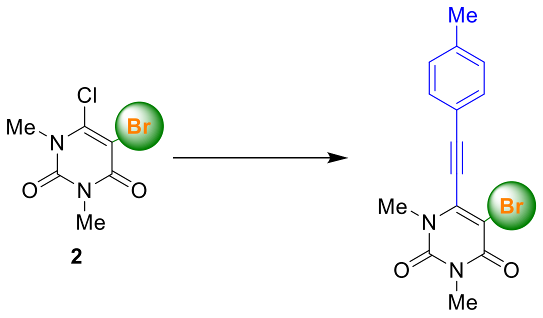

Entry	alkyne [equiv.]	Cat. [mol%]	Additive [mol%]	Base [equiv.]	Solvent	Temp (°C)	Time (h)	Yield
1	1.5	$Pd(PPh_3)_4$ [5]	/	K_3PO_4 [3]	Toluene	100	15	/
2	1.5	$Pd(PPh_3)_4$ [5]	/	K_3PO_4 [3]	Toluene	**80**	15	21
4	1.5	**$PdCl_2$(dppf)** [5]	/	K_3PO_4 [3]	Toluene	80	15	17
5	1.5	**$Pd(OAc)_2$** [5]	/	K_3PO_4 [3]	Toluene	80	15	24
**6***	1.5	$Pd(PPh_3)_4$ [5]	**SPhos [10]**	K_3PO_4 [3]	Toluene	80	15	37
7	1.5	$Pd(PPh_3)_4$ [5]	**Xphos [10]**	K_3PO_4 [3]	Toluene	80	15	29
8	1.5	$Pd(PPh_3)_4$ [5]	**RuPhos [10]**	K_3PO_4 [3]	Toluene	80	15	14
9	1.2	$Pd(PPh_3)Cl_2$ [5]	CuI [5]	NEt_3 [10]	DMSO	100	15	25
10	1.2	$Pd(PPh_3)Cl_2$ [5]	CuI [5]	NEt_3 [10]	DMSO	**25**	15	99
11	1.2	$Pd(PPh_3)Cl_2$ [5]	CuI [5]	NEt_3 [10]	DMSO	25	**6**	99

Experimental data

5-Bromo-6-chloro-1,3-dimethyluracil (2). The compound **2** was obtained as a white solid in 52% yield (3.01 g, 11.9 mmol, R_f = 0.21 heptane/ethyl acetate, 3: 2). ^{1}H NMR (300 MHz, DMSO-d_6) δ = 3.52 (s, 3H), 3.22 (s, 3H). ^{13}C {^{1}H} NMR (75 MHz, DMSO-d_6) δ = 157.7, 149.9, 145.6, 96.6, 35.4, 29.3. HRMS (ESI-TOF): calcd. for $C_6H_7BrClN_2O_2$ $[M+H]^+$ 252.9379, found: 252.9385.

5-Bromo-1,3-dimethyl-6-[2-(4-methylphenyl)ethynyl]uracil (3a). According to general procedure A, compound **3a** was obtained as a brown solid in 99% yield (681 mg, 2.04 mmol, R_f = 0.24 heptane/ethyl acetate, 3: 2); mp: 182 – 184 °C. IR (ATR): $\tilde{\nu}$ [cm^{-1}] = 2213 (m), 1693 (s), 1642 (vs), 1570 (s), 1489 (m), 1432 (s), 1362 (s), 1182 (m), 1009 (s), 822 (s), 745 (s). ^{1}H NMR (300 MHz, Chloroform-*d*) δ = 7.55 – 7.45 (m, 2H), 7.28 – 7.19 (m, 2H), 3.68 (s, 3H), 3.44 (s, 3H), 2.41 (s, 3H). ^{13}C {^{1}H} NMR (75 MHz, Chloroform-*d*) δ = 159.0, 151.0, 141.9, 136.4, 132.2, 129.7, 117.2, 107.3, 102.0, 80.5, 35.5, 29.5, 21.9 (signal of two carbons are absent, which may relate to signal overlap). (EI, 70 eV): m/z (%) = 332 (M^+, 92), 249 (9), 247 (9), 197 (1), 168 (40), 167 (16), 156 (100). (ESI-TOF): calcd. for $C_{15}H_{14}BrN_2O_2$ $[M+H]^+$ 333.0239, found: 333.0242.

5-Bromo-1,3-dimethyl-6-[2-(phenyl)ethynyl]uracil (3b). According to general procedure A, compound **3b** was obtained as a brown solid in 99% yield (376 mg, 1.18 mmol, R_f = 0.24 heptane/ethyl acetate, 3: 2); mp: 181 – 183 °C. IR (ATR): $\tilde{\nu}$ [cm^{-1}] = 2219 (m), 1695 (s), 1646 (vs), 1576 (m), 1425 (s), 1088 (m), 1007 (m), 762 (s), 745 (s), 690 (s). ^{1}H NMR (300 MHz, Chloroform-*d*) δ = 7.60 – 7.51 (m, 2H), 7.51 – 7.28 (m, 3H), 3.62 (s, 3H), 3.37 (s, 3H). ^{13}C {^{1}H} NMR (75 MHz, Chloroform-*d*) δ = 159.0, 151.1, 136.3, 132.3, 131.1, 129.0, 120.3, 106.7, 102.4, 80.7, 35.6, 29.6 (signal of two carbons are absent, which may relate to signal overlap). (EI, 70 eV): m/z (%) = 318 (M^+, 59), 235 (8), 233 (8), 154 (26), 153 (12). (ESI-TOF): calcd. for $C_{14}H_{12}BrN_2O_2$ $[M+H]^+$ 319.0082, found: 319.0088.

5-Bromo-1,3-dimethyl-6-[2-(4-fluorophenyl)ethynyl]uracil (3c). According to general procedure A, compound **3c** was obtained as a brown solid in 84% yield (559 mg, 1.66 mmol, R_f = 0.20 heptane/ethyl acetate, 3: 2); mp: 127 – 129 °C. IR (ATR): $\tilde{\nu}$ [cm^{-1}] = 2921 (m), 2851 (m), 1693 (s), 1603 (m), 1460 (vs), 1324 (m), 1240 (vs), 1168 (m), 1032 (m), 853 (s), 811 (vs), 739 (vs). ^{1}H NMR (300 MHz, Chloroform-*d*) δ = 7.68 – 7.55 (m, 2H), 7.20 – 7.07 (m, 2H), 3.67 (s, 3H), 3.44 (s, 3H). ^{19}F NMR (282 MHz, Chloroform-*d*) δ = -105.7. ^{13}C {^{1}H} NMR (75 MHz, Chloroform-*d*) δ = 164.23 (d, *J* = 254.3 Hz), 159.0, 151.0, 136.1, 134.57 (d, *J* = 8.8 Hz), 116.57 (d, *J* = 22.4 Hz), 116.5, 105.5, 102.5, 80.6, 35.6, 29.6 (signal of two carbons are

absent, which may relate to signal overlap). (EI, 70 eV): m/z (%) = 336 (M^+, 56), 172 (25), 171 (11), 161 (11), 160 (100), 147 (13), 145 (29). (ESI-TOF): calcd. for $C_{14}H_{11}BrFN_2O_2$ $[M+H]^+$ 336.9988, found: 336.9993.

5-Bromo-1,3-dimethyl-6-[2-(4-*tert*-butylphenyl)ethynyl]uracil (3d). According to general procedure A, compound **3d** was obtained as a brown solid in 81% yield (361 mg, 0.963 mmol, R_f = 0.21 heptane/ethyl acetate, 3: 2); mp: 145 – 147 °C. IR (ATR): $\tilde{\nu}$ [cm^{-1}] = 2946 (m), 2209 (m), 1704 (s), 1652 (vs), 1574 (s), 1432 (s), 1180 (m), 1007 (m), 838 (m), 748 (s), 560 (s). ^1H NMR (300 MHz, Chloroform-*d*) δ = 7.57 – 7.52 (m, 2H), 7.47 – 7.42 (m, 2H), 3.67 (s, 3H), 3.43 (s, 3H), 1.33 (s, 9H). ^{13}C {^1H} NMR (75 MHz, Chloroform-*d*) δ = 159.0, 154.9, 151.1, 136.4, 132.1, 126.0, 117.2, 107.3, 102.1, 80.4, 35.5, 35.3, 31.2, 29.5 (signal of four carbons are absent, which may relate to signal overlap). (EI, 70 eV): m/z (%) = 374 (M^+, 79), 362 (17), 361 (100), 59 (98), 333 (12), 198 (12), 195 (13). (ESI-TOF): calcd. for $C_{18}H_{20}BrN_2O_2$ $[M+H]^+$ 375.0708, found: 375.0714.

5-Bromo-1,3-dimethyl-6-[2-(4-trifluoromethylphenyl)ethynyl]uracil (3e). According to general procedure A, compound **3e** was obtained as a black solid in 44% yield (302 mg, 0.881 mmol, R_f = 0.23 heptane/ethyl acetate, 3: 2); mp: 131 – 133 °C. IR (ATR): $\tilde{\nu}$ [cm^{-1}] = 2921 (m), 2202 (m), 1702 (s), 1644 (s), 1574 (vs), 1434 (s), 1250 (vs), 1020 (s), 834 (s), 748 (s). ^1H NMR (300 MHz, Chloroform-*d*) δ 7.78 – 7.65 (m, 4H), 3.69 (s, 3H), 3.45 (s, 3H). ^{19}F NMR (282 MHz, Chloroform-*d*) δ = -63.2. ^{13}C {^1H} NMR (75 MHz, Chloroform-*d*) δ = 158.9, 151.0, 135.6, 132.6 (q, *J* = 33.1 Hz), 132.6, 125.9 (q, *J* = 3.8 Hz), 124.0, 123.6 (q, *J* = 272.6 Hz), 104.2, 103.4, 82.3, 35.6, 29.7 (signal of two carbons are absent, which may relate to signal overlap). (EI, 70 eV): m/z (%) = 386 (M^+,40), 222 (14), 211 (12), 210 (100). (ESI-TOF): calcd. for $C_{15}H_{11}BrF_3N_2O_2$ $[M+H]^+$ 386.9956, found: 386.9964.

5-Bromo-1,3-dimethyl-6-[2-(4-methoxyphenyl)ethynyl]uracil (3f). According to general procedure A, compound **3f** was obtained as a brown solid in 40% yield (280 mg, 0.802 mmol, R_f = 0.18 heptane/ethyl acetate, 3: 2); mp: 164 – 166 °C. IR (ATR): $\tilde{\nu}$ [cm^{-1}] = 2921 (m), 2202 (m), 1702 (s), 1642 (s), 1574 (vs), 1428 (s), 1250 (vs), 1020 (s), 834 (vs), 748 (vs). ^1H NMR (300 MHz, Chloroform-*d*) δ = 7.61 – 7.50 (m, 2H), 7.01 – 6.88 (m, 2H), 3.86 (s, 3H), 3.67 (s, 3H), 3.43 (s, 3H). ^{13}C {^1H} NMR (75 MHz, Chloroform-*d*) δ = 161.9, 159.1, 151.1, 136.6, 134.2, 114.7, 112.2, 107.7, 101.6, 80.3, 55.6, 35.6, 29.5 (signal of two carbons are absent, which may relate to signal overlap). (EI, 70 eV): m/z (%) = 348 (M^+, 98), 265 (9), 263 (9), 212 (22), 184 (44), 172 (59), 169 (21). (ESI-TOF): calcd. for $C_{15}H_{14}BrN_2O_3$ $[M+H]^+$ 349.0188, found: 349.0183.

5-Bromo-1,3-dimethyl-6-[2-(thiophene)ethynyl]uracil (3h). According to general procedure A, compound **3h** was obtained as a brown solid in 77% yield (249 mg, 0.767 mmol, R_f = 0.20 heptane/ethyl acetate, 3: 2); mp: 166 – 168 °C. IR (ATR): $\tilde{\nu}$ [cm^{-1}] = 2213 (m), 1695 (s), 1644 (vs), 1576 (s), 1432 (s), 1009 (s), 785 (s), 745 (vs). ^{1}H NMR (300 MHz, Chloroform-*d*) δ = 7.77 (dd, *J* = 3.0, 1.2 Hz, 1H), 7.40 (dd, *J* = 5.1, 3.0 Hz, 1H), 7.26 (dd, *J* = 5.0, 1.2 Hz, 1H), 3.66 (s, 3H), 3.43 (s, 3H). ^{13}C {^{1}H} NMR (75 MHz, Chloroform-*d*) δ = 159.0, 151.1, 136.3, 132.8, 129.6, 126.7, 119.5, 102.2, 102.1, 80.6, 35.6, 29.6. (EI, 70 eV): m/z (%) = 324 (M^{+}, 60), 241 (8), 239 (8), 188 (14), 160 (31), 159 (11), 149 (12). (ESI-TOF): calcd. for $C_{12}H_{10}BrN_2O_2S$ [M+H]$^{+}$ 324.9646, found: 324.9652.

5-Bromo-1,3-dimethyl-6-[2-(4-dimehtylamino)ethynyl]uracil (3i). According to general procedure A, compound **3i** was obtained as a black solid in 62% yield (88.9 mg, 0.245 mmol, R_f = 0.18 heptane/ethyl acetate, 3: 2); mp: 240 – 242 °C. IR (ATR): $\tilde{\nu}$ [cm^{-1}] = 2921 (s), 2851 (m), 2198 (m), 1691 (s), 1642 (s), 1547 (vs), 1425 (s), 1355 (s), 1174 (s), 999 (s), 809 (vs), 745 (vs). ^{1}H NMR (500 MHz, Chloroform-*d*) δ = 7.48 – 7.41 (m, 2H), 6.67 – 6.61 (m, 2H), 3.65 (s, 3H), 3.40 (s, 3H), 3.04 (s, 6H). ^{13}C {^{1}H} NMR (125 MHz, Chloroform-*d*) δ = 159.2, 151.8, 151.2, 137.1, 133.9, 111.7, 110.5, 106.0, 100.0, 80.6, 40.1, 35.5, 29.4 (signal of three carbons are absent, which may relate to signal overlap). (EI, 70 eV): m/z (%) = 361 (M^{+}, 98), 305 (10), 277 (15), 276 (13), 225 (45), 197 (29), 182 (20). (ESI-TOF): calcd. for $C_{16}H_{17}BrN_3O_2$ [M+H]$^{+}$ 362.0504, found: 362.0508.

5-Bromo-1,3-dimethyl-6-[2-(biphenyl)ethynyl]uracil (3j). According to general procedure A, compound **3j** was obtained as a brown solid in 58% yield (226 mg, 0.572 mmol, R_f = 0.23 heptane/ethyl acetate, 3: 2); mp: 195 – 197 °C. IR (ATR): $\tilde{\nu}$ [cm^{-1}] = 2924 (m), 2217 (m), 1702 (s), 1650 (vs), 1576 (vs), 1428 (s), 1186 (m), 762 (vs), 743 (vs). ^{1}H NMR (300 MHz, Chloroform-*d*) δ = 7.67 (d, *J* = 0.8 Hz, 4H), 7.64 – 7.58 (m, 2H), 7.51 – 7.37 (m, 3H), 3.69 (s, 3H), 3.43 (s, 3H). ^{13}C {^{1}H} NMR (75 MHz, Chloroform-*d*) δ = 159.0, 151.0, 143.9, 139.7, 136.3, 132.8, 129.2, 128.4, 127.5, 127.2, 118.9, 106.7, 102.3, 81.4, 35.6, 29.6 (signal of four carbons are absent, which may relate to signal overlap). (EI, 70 eV): m/z (%) = 394 (M^{+}, 100), 309 (8), 258 (14), 230 (40), 229 (11), 219 (12), 218 (64), 214 (20). (ESI-TOF): calcd. for $C_{20}H_{16}BrN_2O_2$ [M+H]$^{+}$ 395.0395, found: 395.0401.

1,3-Dimethyl-5,6-[2-(4-methylphenyl)ethynyl]uracil (4a). According to general procedure B, compound **4a** was obtained as a brown solid in 92% yield (137 mg, 0.368 mmol, R_f = 0.35 heptane/ethyl acetate, 3: 2); mp: 122 – 124 °C. IR (ATR): $\tilde{\nu}$ [cm^{-1}] = 2921 (m), 1697 (s), 1646 (vs), 1446 (s), 1368 (s), 1174 (m), 1040 (m), 811 (s), 748 (s). ^{1}H NMR (300 MHz, Chloroform-*d*) δ = 7.50 – 7.41 (m, 4H), 7.24 – 7.18 (m, 2H), 7.16 – 7.10 (m, 2H), 3.67 (s, 3H), 3.41 (s,

3H), 2.40 (s, 3H), 2.36 (s, 3H). ^{13}C {^{1}H} NMR (75 MHz, Chloroform-*d*) δ = 161.1, 151.0, 141.6, 138.8, 138.6, 132.2, 131.6, 129.7, 129.2, 120.2, 117.6, 107.2, 103.6, 97.7, 81.9, 80.4, 35.0, 28.7, 21.9, 21.7 (signal of four carbons are absent, which may relate to signal overlap). (EI, 70 eV): m/z (%) = 368 (M^+, 100), 226 (11), 255 (50), 223 (22), 197 (19), 157 (12), 156 (95). (EI): calcd. for $C_{24}H_{20}N_2O_2$ $[M]^+$ 368.15193, found: 368.15202.

1,3-Dimethyl-5,6-[2-(4-*tert*-butylphenyl)ethynyl]uracil (4b). According to general procedure B, compound **4b** was obtained as a brown solid in 47% yield (85 mg, 0.188 mmol, R_f = 0.46 heptane/ethyl acetate, 3: 2); mp: 167 – 169 °C. IR (ATR): $\tilde{\nu}$ [cm^{-1}] = 2957 (m), 1704 (s), 1652 (vs), 1448 (s), 1268 (m), 830 (s), 748 (s). ^{1}H NMR (500 MHz, Chloroform-*d*) δ = 7.55 – 7.51 (m, 2H), 7.51 – 7.47 (m, 2H), 7.44 – 7.41 (m, 2H), 7.37 – 7.33 (m, 2H), 3.68 (s, 3H), 3.41 (s, 3H), 1.34 (s, 9H), 1.32 (s, 9H). ^{13}C {^{1}H} NMR (126 MHz, Chloroform-*d*) δ = 161.1, 154.6, 151.9, 151.0, 138.5, 132.1, 131.5, 126.0, 125.4, 120.3, 117.6, 107.2, 103.6, 97.8, 81.9, 80.4, 35.3, 35.0, 31.3, 31.2, 28.7 (signal of nine carbons are absent, which may relate to signal overlap). (EI, 70 eV): m/z (%) = 452 (M^+, 100), 438 (48), 43 (96), 296 (8), 211 (28), 198 (53), 183 (19). (EI): calcd. for $C_{30}H_{32}N_2O_2$ $[M]^+$ 452.24583, found: 452.24610.

1,3-Dimethyl-5,6-[2-(phenyl)ethynyl]uracil (4c). According to general procedure B, compound **4c** was obtained as a brown solid in 72% yield (98 mg, 0.288 mmol, R_f = 0.30 heptane/ethyl acetate, 3: 2); mp: 91 – 93 °C. IR (ATR): $\tilde{\nu}$ [cm^{-1}] = 2921 (m), 2202 (m), 1702 (s), 1650 (vs), 1442 (s), 1327 (s), 1044 (m), 758 (vs), 686 (vs). ^{1}H NMR (500 MHz, Chloroform-*d*) δ = 7.61 – 7.58 (m, 2H), 7.57 – 7.52 (m, 2H), 7.50 – 7.46 (m, 1H), 7.43 – 7.39 (m, 2H), 7.35 – 7.31 (m, 3H), 3.69 (s, 3H), 3.43 (s, 3H). ^{13}C {^{1}H} NMR (126 MHz, Chloroform-*d*) δ = 161.0, 150.9, 138.6, 132.3, 131.7, 130.9, 129.0, 128.7, 128.5, 123.2, 120.6, 106.7, 103.7, 97.6, 82.4, 80.7, 35.0, 28.8 (signal of four carbons are absent, which may relate to signal overlap). (EI, 70 eV): m/z (%) = 340 (M^+, 100), 266 (11), 207 (11), 169 (8), 149 (14), 143 (12), 142 (91). (EI): calcd. for $C_{22}H_{16}N_2O_2$ $[M]^+$ 340.12063, found: 340.12119.

1,3-Dimethyl-5,6-[2-(4-methoxyphenyl)ethynyl]uracil (4d). According to general procedure B, compound **4d** was obtained as a brown solid in 37% yield (59 mg, 0.147 mmol, R_f = 0.21 heptane/ethyl acetate, 3: 2); mp: 110 – 112 °C. IR (ATR): $\tilde{\nu}$ [cm^{-1}] = 2926 (m), 2192 (m), 1697 (s), 1650 (vs), 1560 (s), 1434 (s), 1246 (vs), 1017 (s), 828 (s). ^{1}H NMR (500 MHz, Chloroform-*d*) δ = 7.53 – 7.50 (m, 2H), 7.50 – 7.47 (m, 2H), 6.92 – 6.89 (m, 2H), 6.88 – 6.84 (m, 2H), 3.85 (s, 3H), 3.83 (s, 3H), 3.66 (s, 3H), 3.41 (s, 3H). ^{13}C {^{1}H} NMR (126 MHz, Chloroform-*d*) δ = 161.7, 161.1, 160.0, 151.0, 138.5, 134.0, 133.2, 115.5, 114.7, 114.1, 112.6, 107.4, 103.3, 97.4, 81.3, 80.3, 55.6, 55.5, 35.0, 28.7 (signal of four carbons are absent, which may relate to signal overlap). (EI, 70 eV): m/z (%) = 400 (M^+, 100), 385 (15), 200 (13),

173 (13), 172 (96), 159 (11), 157 (30). (EI): calcd. for $C_{24}H_{20}N_2O_4$ $[M]^+$ 400.14176, found: 400.14230.

1,3-Dimethyl-5,6-[2-(4-dimethylaminophenyl)ethynyl]uracil (4e). According to general procedure B, compound **4e** was obtained as a brown solid in 86% yield (147 mg, 0.345 mmol, R_f = 0.17 heptane/ethyl acetate, 3: 2); mp: 157 – 159 °C. IR (ATR): $\tilde{\nu}$ [cm^{-1}] = 2917 (m), 2173 (s), 1697 (s), 1601 (vs), 1518 (vs), 1360 (vs), 1170 (vs), 809 (vs). ^{1}H NMR (250 MHz, Chloroform-*d*) δ = 7.48 – 7.41 (m, 4H), 6.66 – 6.61 (m, 4H), 3.65 (s, 3H), 3.39 (s, 3H), 3.03 (s, 6H), 2.99 (s, 6H). ^{13}C {^{1}H} NMR (63 MHz, Chloroform-*d*) δ = 161.4, 151.6, 151.1, 150.2, 138.2, 133.8, 132.8, 111.9, 111.7, 110.4, 109.8, 106.7, 102.5, 98.4, 80.9, 80.7, 40.3, 40.1, 34.8, 28.6 (signal of six carbons are absent, which may relate to signal overlap). (EI, 70 eV): m/z (%) = 426 (M^+, 100), 262 (14), 185 (23), 183 (14), 17 (16), 169 (10). (EI): calcd. for $C_{26}H_{26}N_4O_2$ $[M]^+$ 426.20503, found: 426.20633.

1,3-Dimethyl-5-[2-(4-fluorophenyl)ethynyl]-6-[2-(4-methylphenyl)-ethynyl]uracil (4h). According to general procedure C, compound **4h** was obtained as a brown solid in 47% yield (70 mg, 0.188 mmol, R_f = 0.35 heptane/ethyl acetate, 3: 2); mp: 173 – 175 °C. IR (ATR): $\tilde{\nu}$ [cm^{-1}] = 2200 (m), 1702 (s), 1648 (vs), 1446 (s), 1331 (s), 1090 (s), 828 (vs), 745 (s). ^{1}H NMR (500 MHz, Chloroform-*d*) δ = 7.54 – 7.49 (m, 2H), 7.48 – 7.44 (m, 2H), 7.23 – 7.19 (m, 2H), 7.05 – 6.99 (m, 2H), 3.67 (s, 3H), 3.41 (s, 3H), 2.41 (s, 3H). ^{19}F NMR (282 MHz, Chloroform-*d*) δ = -110.4. ^{13}C {^{1}H} NMR (126 MHz, Chloroform-*d*) δ = 162.8 (d, *J* = 250.0 Hz), 161.0, 150.9, 141.8, 138.9, 133.6 (d, *J* = 8.6 Hz), 129.8, 119.4, 119.4, 117.5, 115.8 (d, *J* = 22.2 Hz), 107.4, 103.2, 96.3, 82.3 (d, *J* = 1.7 Hz), 80.3, 35.0, 28.8, 21.9 (signal of four carbons are absent, which may relate to signal overlap). (EI, 70 eV): m/z (%) = 372 (M^+, 100), 287 (11), 272 (7), 258 (11), 157 (20), 156 (89). (EI): calcd. for $C_{23}H_{17}FN_2O_2$ $[M]^+$ 372.12686, found: 372.12648.

1,3-Dimethyl-5-(4-methylphenyl)-6-[2-(4-methylphenyl)ethynyl]uracil (5a). According to general procedure D, compound **5a** was obtained as a brown solid in 62% yield (65 mg, 0.189 mmol, R_f = 0.22 heptane/ethyl acetate, 3: 2); mp: 191 – 193 °C. IR (ATR): $\tilde{\nu}$ [cm^{-1}] = 1685 (s), 1640 (vs), 1508 (s), 1452 (s), 1127 (m), 1022 (m), 820 (s), 748 (s). ^{1}H NMR (300 MHz, Chloroform-*d*) δ = 7.42 – 7.35 (m, 2H), 7.25 – 7.19 (m, 2H), 7.14 (s, 3H), 3.69 (s, 3H), 3.43 (s, 3H), 2.40 (s, 3H), 2.36 (s, 3H).^{13}C {^{1}H} NMR (75 MHz, Chloroform-*d*) δ = 162.2, 151.7, 141.0, 138.0, 134.1, 131.8, 130.8, 130.2, 129.5, 128.7, 118.6, 117.7, 104.5, 80.8, 34.6, 28.6, 21.8, 21.5 (signal of four carbons are absent, which may relate to signal overlap). (EI, 70 eV): m/z (%) = 344 (M^+, 100), 329 (53), 286 (26), 258 (47), 244 (40), 229 (16), 215 (13). (ESI-TOF): calcd. for $C_{22}H_{21}N_2O_2$ $[M+H]^+$ 345.1603, found: 345.1604.

1,3-Dimethyl-5-(4-dimethylaminophenyl)-6-[2-phenylethynyl]uracil (5b). According to general procedure D, compound **5b** was obtained as a brown solid in 52% yield (176 mg, 0.491 mmol, R_f = 0.14 heptane/ethyl acetate, 3: 2); mp: 105 – 107 °C. IR (ATR): $\tilde{\nu}$ [cm^{-1}] = 3329 (m), 1693 (s), 1638 (vs), 1611 (vs), 1514 (s), 1442 (s), 1357 (s), 813 (s), 748 (s). ^{1}H NMR (500 MHz, Chloroform-*d*) δ = 7.44 – 7.40 (m, 2H), 7.40 – 7.36 (m, 1H), 7.35 – 7.30 (m, 4H), 6.79 – 6.75 (m, 2H), 3.70 (s, 3H), 3.43 (s, 3H), 2.99 (s, 6H). ^{13}C {^{1}H} NMR (126 MHz, Chloroform-*d*) δ = 162.5, 151.7, 150.5, 132.8, 131.9, 131.7, 130.2, 128.7, 121.0, 120.6, 119.2, 111.8, 103.4, 81.7, 40.7, 34.5, 28.6 (signal of five carbons are absent, which may relate to signal overlap). (EI, 70 eV): m/z (%) = 359 (M$^+$, 100), 301 (7), 273 (16), 259 (14), 180 (12). (ESI-TOF): calcd. for $C_{22}H_{22}N_3O_2$ [M+H]$^+$ 360.1712, found: 360.1717.

1,3-Dimethyl-5-(4-trifluoromethylphenyl)-6-[2-phenylethynyl]uracil (5c). According to general procedure D, compound **5c** was obtained as a brown solid in 40% yield (145 mg, 0.378 mmol, R_f = 0.19 heptane/ethyl acetate, 3: 2); mp: 136 – 139 °C. IR (ATR): $\tilde{\nu}$ [cm^{-1}] = 1702 (s), 1640 (vs), 1615 (s), 1436 (s), 1314 (s), 1120 (vs), 1065 (s), 748 (s). ^{1}H NMR (500 MHz, Chloroform-*d*) δ = 7.72 – 7.67 (m, 2H), 7.66 – 7.62 (m, 2H), 7.44 – 7.39 (m, 1H), 7.36 – 7.31 (m, 2H), 7.22 – 7.19 (m, 2H), 3.72 (s, 3H), 3.45 (s, 3H). ^{19}F NMR (471 MHz, Chloroform-*d*) δ = -62.6. ^{13}C {^{1}H} NMR (126 MHz, Chloroform-*d*) δ = 161.7, 151.4, 137.1, 134.9, 131.9, 131.5, 130.8, 130.2 (q, *J* = 32.5 Hz), 128.9, 124.9 (q, *J* = 3.8 Hz), 124.3 (q, *J* = 272.1 Hz), 120.2, 117.4, 104.9, 80.5, 34.7, 28.7 (signal of four carbons are absent, which may relate to signal overlap). (EI, 70 eV): m/z (%) = 323 (19), 265 (29), 237 (58), 222 (13), 163 (12), 142 (100). (EI): calcd. for $C_{21}H_{15}F_3N_2O_2$ [M]$^+$ 384.10801, found: 384.10687.

1,3-Dimethyl-5-(4-dimethylaminophenyl)-6-[2-(4-methylphenyl)ethynyl]uracil (5d). According to general procedure D, compound **5d** was obtained as a brown solid in 54% yield (183 mg, 0.490 mmol, R_f = 0.15 heptane/ethyl acetate, 3: 2); mp: 126 – 128 °C. IR (ATR): $\tilde{\nu}$ [cm^{-1}] = 1697 (s), 642 (vs), 1580 (s), 1430 (s), 1306 (s), 1197 (s), 1125 (s), 813 (s), 748 (s). ^{1}H NMR (300 MHz, Chloroform-*d*) δ = 7.45 – 7.38 (m, 2H), 7.24 – 7.18 (m, 2H), 7.16 – 7.09 (m, 2H), 6.80 – 6.73 (m, 2H), 3.68 (s, 3H), 3.42 (s, 3H), 2.99 (s, 6H), 2.35 (s, 3H). ^{13}C {^{1}H} NMR (75 MHz, Chloroform-*d*) δ = 162.4, 151.6, 150.4, 140.7, 133.0, 131.8, 131.6, 129.4, 120.7, 118.8, 118.0, 111.8, 103.9, 81.3, 40.6, 34.5, 28.6, 21.8 (signal of five carbons are absent, which may relate to signal overlap). (EI, 70 eV): m/z (%) = 373 (M$^+$, 100), 358 (9), 288 (14), 273 (15), 261 (9), 244 (9), 187 (15). (ESI-TOF): calcd. for $C_{23}H_{24}N_3O_2$ [M+H]$^+$ 374.1868, found: 374.1864.

1,3-Dimethyl-5-(4-trifluoromethylphenyl)-6-[2-(4-methylphenyl)ethynyl]uracil (5e). According to general procedure D, compound **5e** was obtained as a brown solid in 41% yield (150 mg, 0.377 mmol, R_f = 0.22 heptane/ethyl acetate, 3: 2); mp: 140 – 142 °C. IR (ATR): $\tilde{\nu}$ [cm^{-1}] = 2211 (m), 1700 (s), 1644 (vs), 158 (s), 1430 (s), 1322 (vs), 1162 (s), 1067 (vs), 840 (s). ^{1}H NMR (300 MHz, Chloroform-*d*) δ = 7.66 (q, *J* = 8.4 Hz, 4H), 7.11 (q, *J* = 8.3 Hz, 4H), 3.71 (s, 3H), 3.44 (s, 3H), 2.36 (s, 3H). ^{19}F NMR (471 MHz, Chloroform-*d*) δ = -62.6. ^{13}C {^{1}H} NMR (75 MHz, Chloroform-*d*) δ = 161.7, 151.5, 141.6, 137.2 (d, *J* = 1.7 Hz), 135.1, 131.8, 131.5, 130.1 (q, *J* = 32.4 Hz), 129.6, 124.9 (q, *J* = 3.8 Hz), 124.3 (q, *J* = 272.1 Hz), 117.1, 117.0, 105.5, 80.2, 34.7, 28.6, 21.8 (signal of four carbons are absent, which may relate to signal overlap). (EI, 70 eV): m/z (%) = 398 (M$^+$, 80), 383 (61), 340 (25), 312 (35), 298 (31), 283 (19), 272 (17). (ESI-TOF): calcd. for $C_{22}H_{18}F_3N_2O_2$ [M+H]$^+$ 399.1320, found: 399.1322.

1,3-Dimethyl-5-[2-(4-methylphenyl)ethenyl]-6-[2-(4-methylphenyl)ethynyl]uracil (5f). According to general procedure D, compound **5f** was obtained as a brown solid in 75% yield (125 mg, 0.338 mmol, R_f = 0.36 heptane/ethyl acetate, 3: 2); mp: 152 – 154 °C. IR (ATR): $\tilde{\nu}$ [cm^{-1}] = 2211 (m), 1702 (s), 1644 (vs), 1510 (s), 1432 (s), 1212 (s), 1131 (m), 838 (s), 774 (m). ^{1}H NMR (300 MHz, Chloroform-*d*) δ = 8.03 (d, *J* = 16.2 Hz, 1H), 7.51 – 7.46 (m, 2H), 7.44 – 7.37 (m, 2H), 7.33 – 7.20 (m, 3H), 7.15 (d, *J* = 7.9 Hz, 2H), 3.67 (s, 3H), 3.43 (s, 3H), 2.43 (s, 3H), 2.35 (s, 3H). ^{13}C {^{1}H} NMR (75 MHz, Chloroform-*d*) δ = 161.6, 150.8, 141.3, 137.8, 135.6, 133.2, 133.1, 131.8, 129.8, 129.5, 126.5, 120.4, 117.7, 114.4, 107.8, 80.0, 34.6, 28.4, 21.9, 21.4 (signal of four carbons are absent, which may relate to signal overlap). (EI, 70 eV): m/z (%) = 370 (M$^+$, 98), 355 (100), 312 (51), 298 (68), 284 (79), 270 (82), 255 (25). (EI): calcd. for $C_{24}H_{22}N_2O_2$ [M]$^+$ 370.16758, found: 370.16784.

(*E*)-1,3-Dimethyl-6-(*p*-tolylethynyl)-5-(4-(trifluoromethyl)styryl)pyrimidine-2,4(1*H*,3*H*)-dione (5g). According to general procedure D, compound **5g** was obtained as a brown solid in 41% yield (78.5 mg, 0.185 mmol, R_f = 0.32 heptane/ethyl acetate, 3: 2); mp: 127 – 129 °C. IR (ATR): $\tilde{\nu}$ [cm^{-1}] = 1702 (s), 1648 (s), 1510 (m), 1320 (vs), 1162 (s), 1065 (s), 816 (s), 727 (m). ^{1}H NMR (300 MHz, Chloroform-*d*) δ = 8.09 (d, *J* = 16.2 Hz, 1H), 7.57 (m, 4H), 7.52 – 7.45 (m, 2H), 7.38 (d, *J* = 16.2 Hz, 1H), 7.31 – 7.21 (m, 2H), 3.70 (s, 3H), 3.44 (s, 3H), 2.43 (s, 3H). ^{19}F NMR (282 MHz, Chloroform-*d*) δ = -62.4. ^{13}C {^{1}H} NMR (75 MHz, Chloroform-*d*) δ = 161.5, 150.8, 142.0 (d, *J* = 1.5 Hz), 141.8, 134.6, 131.8, 131.5, 129.9, 129.3 (q, *J* = 32.3 Hz), 126.6, 125.8 (q, *J* = 3.9 Hz), 124.4 (q, *J* = 271.9 Hz), 123.9, 117.4, 113.5, 108.5, 79.7, 34.7, 28.4, 21.9 (signal of four carbons are absent, which may relate to signal overlap). (EI, 70 eV): m/z (%) = 424 (M$^+$, 60), 409 (98), 366 (15), 338 (36), 324 (42), 267 (10), 183 (22). (EI): calcd. for $C_{24}H_{19}F_3N_2O_2$ [M]$^+$ 424.13931, found: 424.13907.

1,3-Dimethyl-5-(4-dimethylaminophenyl)-6-[2-(biphenyl)ethynyl]uracil (5i). According to general procedure D, compound **5i** was obtained as a brown solid in 52% yield (85.2 mg, 0.199 mmol, R_f = 0.15 heptane/ethyl acetate, 3: 2); mp: 177 – 179 °C. IR (ATR): $\tilde{\nu}$ [cm^{-1}] = 2921 (m), 1693 (s), 1642 (vs), 1510 (s), 1432 (s), 1308 (s), 1215 (s), 816 (s). ^{1}H NMR (300 MHz, Chloroform-*d*) δ = 7.61 – 7.53 (m, 4H), 7.50 – 7.35 (m, 7H), 6.83 – 6.76 (m, 2H), 3.72 (s, 3H), 3.44 (s, 3H), 3.01 (s, 6H). ^{13}C {^{1}H} NMR (75 MHz, Chloroform-*d*) δ = 162.4, 151.7, 150.5, 142.9, 139.9, 132.8, 132.4, 131.7, 129.1, 128.2, 127.3, 127.2, 120.6, 119.8, 119.2, 111.8, 103.4, 82.4, 40.7, 34.5, 28.6 (signal of seven carbons are absent, which may relate to signal overlap). (EI, 70 eV): m/z (%) = 435 (M^+, 100), 350 (14), 335 (13), 331 (10), 281 (21), 218 (37), 207 (18). (ESI-TOF): calcd. for $C_{28}H_{26}N_3O_2$ $[M+H]^+$ 436.2025, found: 436.2025.

1,3-Dimethyl-5-(4-*tert*-butylphenyl)-6-[2-(4-*tert*-butylphenyl)ethynyl]uracil (5j). According to general procedure D, compound **5j** was obtained as a brown solid in 74% yield (43.6 mg, 0.102 mmol, R_f = 0.30 heptane/ethyl acetate, 3: 2); mp: 169 – 171 °C. IR (ATR): $\tilde{\nu}$ [cm^{-1}] = 2905 (m), 1695 (s), 1644 (vs), 1456 (s), 1306 (m), 1106 (m), 999 (m), 832 (s). ^{1}H NMR (500 MHz, Chloroform-*d*) δ = 7.47 – 7.39 (m, 4H), 7.33 – 7.29 (m, 2H), 7.14 – 7.11 (m, 2H), 3.70 (s, 3H), 3.44 (s, 3H), 1.37 (s, 9H), 1.29 (s, 9H). ^{13}C {^{1}H} NMR (126 MHz, Chloroform-*d*) δ = 162.2, 154.1, 151.7, 151.1, 134.4, 131.8, 130.6, 130.3, 125.7, 125.0, 118.8, 117.8, 104.6, 80.9, 35.2, 34.8, 34.6, 31.5, 31.2, 28.6 (signal of eight carbons are absent, which may relate to signal overlap). (EI, 70 eV): m/z (%) = 428 (M^+, 36), 413 (16), 371 (72), 357 (12), 315 (100), 258 (15), 230 (13). (ESI-TOF): calcd. for $C_{28}H_{33}N_2O_2$ $[M+H]^+$ 429.2542, found: 429.2534.

1,3-Dimethyl-5-(4-methylphenyl)-6-[2-(4-dimethylaminophenyl)ethynyl]uracil (5k). According to general procedure D, compound **5k** was obtained as a brown solid in 84% yield (263 mg, 0.703 mmol, R_f = 0.13 heptane/ethyl acetate, 3: 2); mp: 175 – 177 °C. IR (ATR): $\tilde{\nu}$ [cm^{-1}] = 2917 (m), 1697 (s), 1522 (s), 1415 (s), 1310 (s), 1131 (s), 1061 (s), 818 (vs). ^{1}H NMR (300 MHz, Chloroform-*d*) δ = 7.43 – 7.37 (m, 2H), 7.25 – 7.19 (m, 2H), 7.13 – 7.08 (m, 2H), 6.60 – 6.54 (m, 2H), 3.68 (s, 3H), 3.42 (s, 3H), 3.00 (s, 6H), 2.40 (s, 3H). ^{13}C {^{1}H} NMR (75 MHz, Chloroform-*d*) δ = 162.3, 151.8, 151.3, 137.6, 134.9, 133.4, 130.8, 130.6, 128.5, 116.8, 111.6, 107.1, 106.7, 80.4, 40.1, 34.5, 28.5, 21 (signal of five carbons are absent, which may relate to signal overlap). (EI, 70 eV): m/z (%) = 373 (M^+, 100), 316 (2), 287 (3), 261 (3), 228 (2), 215 (3), 202 (4). (ESI-TOF): calcd. for $C_{23}H_{24}N_3O_2$ $[M+H]^+$ 374.1868, found: 374.1861.

1,3-Dimethyl-5-(4-trifluoromethylphenyl)-6-[2-(4-dimethylaminophenyl)ethynyl]uracil (5l). According to general procedure D, compound **5l** was obtained as a brown solid in 70% yield (124 mg, 0.289 mmol, R_f = 0.08 heptane/ethyl acetate, 3: 2); mp: 162 – 164 °C. IR

(ATR): ṽ [cm^{-1}] = 2196 (m), 1693 (s), 1607 (vs), 1574 (vs), 1430 (s), 1310 (s), 1120 (s), 1057 (s). ^{1}H NMR (300 MHz, Chloroform-*d*) δ = δ 7.71 – 7.62 (m, 4H), 7.08 – 7.02 (m, 2H), 6.61 – 6.56 (m, 2H), 3.71 (s, 3H), 3.44 (s, 3H), 3.01 (s, 6H). ^{19}F NMR (282 MHz, Chloroform-*d*) δ = -62.5. ^{13}C {^{1}H} NMR (63 MHz, Chloroform-*d*) δ = 161.8, 151.6, 151.5, 137.7, 135.9, 133.5, 131.6, 129.8 (q, *J* = 64.8, 32.3 Hz), 124.8 (q, *J* = 3.8 Hz), 124.4 (q, *J* = 544.1, 272.0 Hz), 115.3, 111.8, 108.4, 106.1, 80.1, 40.2, 34.7, 28.6 (signal of five carbons are absent, which may relate to signal overlap). (EI, 70 eV): m/z (%) = 427 (M^+, 100), 369 (12), 341 (17), 327 (13), 312 (5), 298 (9), 273 (4). (ESI-TOF): calcd. for $C_{23}H_{21}F_3N_3O_2$ $[M+H]^+$ 428.1586, found: 428.1587.

1,3-Dimethyl-5-(4-dimethylaminophenyl)-6-[2-(4-dimethylaminophenyl)ethynyl]uracil (5m). According to general procedure D, compound **5m** was obtained as a brown solid in 26% yield (86.9 mg, 0.216 mmol, R_f = 0.17 heptane/ethyl acetate, 3: 2); mp: 188 – 190 °C. IR (ATR): ṽ [cm^{-1}] = 2194 (m), 1691 (s), 1605 (vs), 1520 (s), 1409 (s), 1310 (s), 1188 (s), 1015 (s), 818 (vs). ^{1}H NMR (500 MHz, Chloroform-*d*) δ = 7.46 – 7.40 (m, 2H), 7.21 – 7.14 (m, 2H), 6.81 – 6.74 (m, 2H), 6.60 – 6.54 (m, 2H), 3.68 (s, 3H), 3.42 (s, 3H), 3.00 – 2.99 (m, 12H). ^{13}C {^{1}H} NMR (126 MHz, Chloroform-*d*) δ = 162.6, 151.8, 151.2, 150.3, 133.9, 133.4, 131.7, 121.2, 117.2, 111.9, 111.6, 107.1, 106.4, 80.8, 40.8, 40.1, 34.5, 28.5 (signal of six carbons are absent, which may relate to signal overlap). (EI, 70 eV): m/z (%) = 402 (M^+, 100), 388 (15), 361 (5), 345 (5), 316 (5), 301 (5), 273 (5). (ESI-TOF): calcd. for $C_{24}H_{27}N_4O_2$ $[M+H]^+$ 403.2129, found: 403.2135.

1,3-Dimethyl-6-[2-(4-fluorophenyl)ethynyl]-5-(4-dimethylaminophenyl)uracil (5p). According to general procedure D, compound **5p** was obtained as a brown solid in 38% yield (64.6 mg, 0.171 mmol, R_f = 0.18 heptane/ethyl acetate, 3: 2); mp: 130 – 132 °C. IR (ATR): ṽ [cm^{-1}] = 2853 (m), 2209 (m), 1638 (vs), 1504 (s), 1312 (s), 1219 (s), 1133 (s), 846 (s). ^{1}H NMR (300 MHz, Chloroform-*d*) δ = 7.43 – 7.37 (m, 2H), 7.34 – 7.27 (m, 2H), 7.06 – 6.98 (m, 2H), 6.79 – 6.74 (m, 2H), 3.68 (s, 3H), 3.43 (s, 3H), 2.99 (s, 6H). ^{19}F NMR (282 MHz, Chloroform-*d*) δ = -107.3. ^{13}C {^{1}H} NMR (75 MHz, Chloroform-*d*) δ = 163.5 (d, *J* = 253.0 Hz), 162.3, 151.5, 150.4, 133.9 (d, *J* = 8.7 Hz), 132.6, 131.5, 120.4, 119.2, 117.1 (d, *J* = 3.5 Hz), 116.1 (d, *J* = 22.3 Hz), 111.7, 102.1, 81.4, 40.5, 34.4, 28.5 (signal of five carbons are absent, which may relate to signal overlap). (EI, 70 eV): m/z (%) = 377 (M^+, 95), 320 (22), 292 (42), 276 (15), 265 (13), 248 (16), 207 (13). (EI): calcd. for $C_{22}H_{20}FN_3O_2$ $[M]^+$ 377.15341, found: 377.15319.

1,3-Dimethyl-6-[2-(4-trifluoromethylphenyl)ethynyl]-5-(4-dimethylaminophenyl)uracil (5q). According to general procedure D, compound **5q** was obtained as a brown solid in 33% yield (37.4 mg, 87.5 µmol, R_f = 0.15 heptane/ethyl acetate, 3: 2); mp: 148 – 150 °C. IR (ATR): $\tilde{\nu}$ [cm^{-1}] = 2188 (s), 1638 (vs), 1524 (s), 1322 (vs), 1188 (s), 1120 (vs), 1065 (vs), 840 (s).^{1}H NMR (500 MHz, Chloroform-*d*) δ = 7.70 – 7.63 (m, 4H), 7.06 – 7.02 (m, 2H), 6.59 – 6.54 (m, 2H), 3.70 (s, 3H), 3.44 (s, 3H), 3.01 (s, 6H). ^{19}F NMR (471 MHz, Chloroform-*d*) δ = -62.5. ^{13}C {^{1}H} NMR (75 MHz, Chloroform-*d*) δ = 161.8, 151.6, 151.6, 137.7, 136.0, 133.5, 131.6, 129.8 (q, *J* = 32.4 Hz), 124.8 (q, *J* = 3.8 Hz), 124.4 (q, *J* = 272.0 Hz), 115.3, 111.7, 108.5, 106.0, 80.1, 40.1, 34.7, 28.6 (signal of five carbons are absent, which may relate to signal overlap). (EI, 70 eV): m/z (%) = 427 (M^{+}, 100), 369 (13), 326 (11), 312 (5), 298 (10), 214 (9), 170 (26). (ESI-TOF): calcd. for $C_{23}H_{21}F_{3}N_{3}O_{2}$ [M+H]$^{+}$ 428.1586, found: 428.1575.

6-((4-(Dimethylamino)phenyl)ethynyl)-1,3-dimethyl-5-(thiophen-3-yl)pyrimidine-2,4(1*H*,3*H*)-dione (5r). According to general procedure D, compound **5r** was obtained as a brown solid in 95% yield (143 mg, 0.391 mmol, R_f = 0.17 heptane/ethyl acetate, 3: 2); mp: 207 – 209 °C. IR (ATR): $\tilde{\nu}$ [cm^{-1}] = 2919 (m), 1691 (s), 1568 (s), 1409 (s), 1364 (vs), 1184 (vs), 1011 (s), 801 (vs). ^{1}H NMR (300 MHz, Chloroform-*d*) δ = 7.65 (dd, *J* = 3.0, 1.3 Hz, 1H), 7.43 (dd, *J* = 5.0, 1.3 Hz, 1H), 7.33 (dd, *J* = 5.0, 3.0 Hz, 1H), 7.28 – 7.17 (m, 2H), 6.65 – 6.58 (m, 2H), 3.69 (s, 3H), 3.43 (s, 3H), 3.01 (s, 6H). ^{13}C {^{1}H} NMR (75 MHz, Chloroform-*d*) δ = 161.9, 151.5, 151.4, 134.5, 133.5, 132.9, 130.6, 129.8, 126.2, 123.6, 111.7, 107.7, 106.6, 80.7, 40.1, 34.6, 28.6 (signal of three carbons are absent, which may relate to signal overlap). (EI, 70 eV): m/z (%) = 364 (M^{+}, 86), 349 (28), 332 (80), 321 (67), 308 (90), 293 (93), 280(93). (ESI-TOF): calcd. for $C_{20}H_{20}N_{3}O_{2}S$ [M+H]$^{+}$ 366.1276, found: 366.1279.

5-(4-(Dimethylamino)phenyl)-1,3-dimethyl-6-(thiophen-3-ylethynyl)pyrimidine-2,4(1*H*,3*H*)-dione (5s). According to general procedure D, compound **5s** was obtained as a brown solid in 40% yield (66.8 mg, 0.183 mmol, R_f = 0.13 heptane/ethyl acetate, 3: 2); mp: 142 – 144 °C. IR (ATR): $\tilde{\nu}$ [cm^{-1}] = 2213 (m), 1634 (vs), 1524 (s), 1407 (s), 1304 (s), 1201 (m), 1127 (s), 791 (s). ^{1}H NMR (300 MHz, Chloroform-*d*) δ = 7.47 – 7.36 (m, 3H), 7.33 – 7.24 (m, 1H), 7.04 – 6.98 (m, 1H), 6.80 – 6.73 (m, 2H), 3.67 (s, 3H), 3.43 (s, 3H), 2.99 (s, 6H). ^{13}C {^{1}H} NMR (75 MHz, Chloroform-*d*) δ = 162.5, 151.7, 150.5, 132.9, 131.7, 131.5, 129.5, 126.2, 120.6, 120.3, 119.0, 111.8, 98.8, 81.5, 40.7, 34.5, 28.6 (signal of three carbons are absent, which may relate to signal overlap). (EI, 70 eV): m/z (%) = 365 (M^{+}, 100), 307 (7), 279 (14), 265 (20), 236 (9), 209 (5), 183 (14). (ESI-TOF): calcd. for $C_{20}H_{20}N_{3}O_{2}S$ [M+H]$^{+}$ 366.1276, found: 366.1270.

5-(Furan-3-yl)-1,3-dimethyl-6-(*p*-tolylethynyl)pyrimidine-2,4(1*H*,3*H*)-dione (5t). According to general procedure D, compound **5t** was obtained as a brown solid in 76% yield (110 mg, 0.342 mmol, R_f = 0.38 heptane/ethyl acetate, 3: 2); mp: 128 – 130 °C. IR (ATR): $\tilde{\nu}$ [cm^{-1}] = 1638 (vs), 1508 (m), 1450 (s), 1316 (m), 1164 (m), 1028 (s), 811 (s), 750 (vs). ^1H NMR (300 MHz, Chloroform-*d*) δ = 8.06 (dd, *J* = 1.6, 0.8 Hz, 1H), 7.48 – 7.46 (m, 1H), 7.41 – 7.36 (m, 2H), 7.24 – 7.18 (m, 2H), 6.98 (dd, *J* = 1.9, 0.8 Hz, 1H), 3.70 (s, 3H), 3.43 (s, 3H), 2.40 (s, 3H). ^{13}C {^1H} NMR (75 MHz, Chloroform-*d*) δ = 161.6, 151.2, 143.9, 141.7, 141.4, 132.3, 131.8, 129.7, 117.6, 117.0, 111.3, 110.5, 105.5, 81.1, 34.5, 28.7, 21.9 (signal of two carbons are absent, which may relate to signal overlap). (EI, 70 eV): m/z (%) = 320 (M^+, 62), 291 (15), 277 (14), 262 (43), 235 (53), 220 (33), 208 (100). (ESI-TOF): calcd. for $C_{19}H_{17}N_2O_3$ $[M+H]^+$ 321.1239, found: 321.1234.

^{1}H, ^{13}C, and ^{19}F NMR spectra

5-Bromo-6-chloro-1,3-dimethyluracil (2)

	Parameter	Value
1	Solvent	DMSO
2	Temperature	298.2
3	Pulse Sequence	zg30
4	Experiment	1D
5	Number of Scans	16
6	Receiver Gain	203.0
7	Relaxation Delay	2.0000
8	Pulse Width	10.0000
9	Spectrometer Frequency	300.13
10	Spectral Width	6188.1
11	Lowest Frequency	-1240.3
12	Nucleus	1H
13	Acquired Size	32768
14	Spectral Size	65536

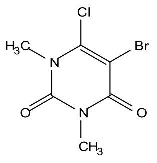

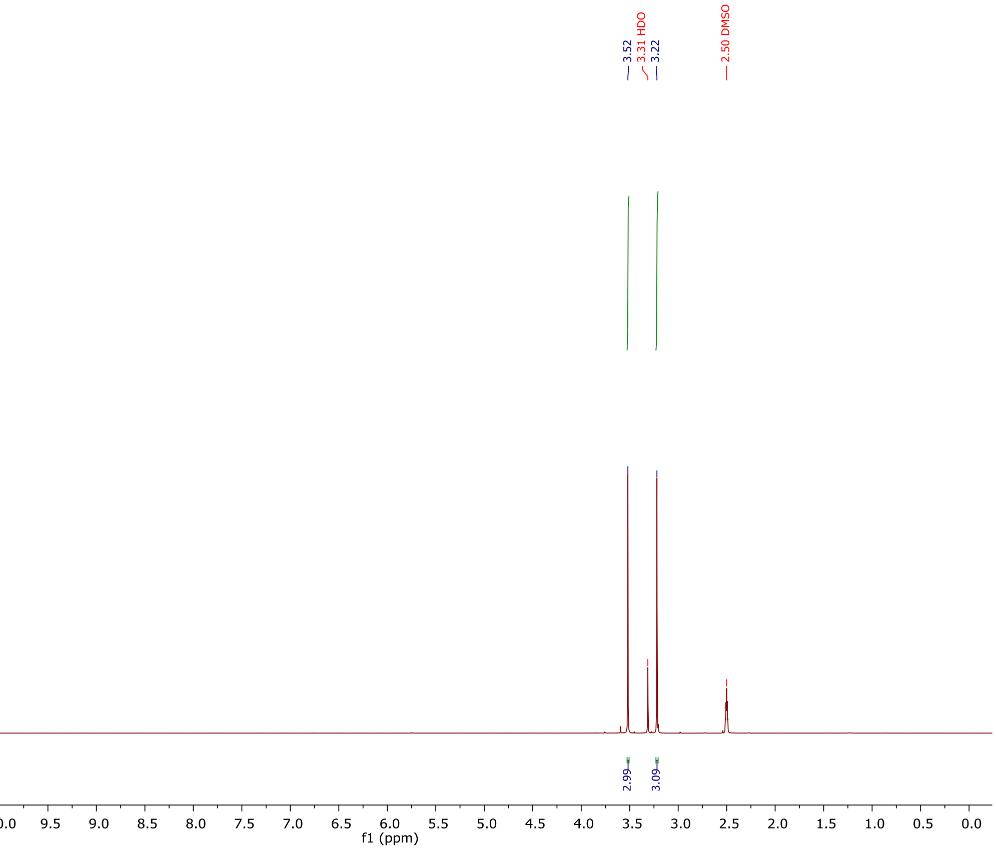

	Parameter	Value
1	Solvent	DMSO
2	Temperature	298.2
3	Pulse Sequence	zgpg30
4	Experiment	1D
5	Number of Scans	1024
6	Receiver Gain	2050.0
7	Relaxation Delay	2.0000
8	Pulse Width	10.0000
9	Spectrometer Frequency	75.48
10	Spectral Width	18028.8
11	Lowest Frequency	-1503.7
12	Nucleus	13C
13	Acquired Size	32768
14	Spectral Size	65536

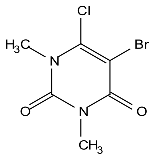

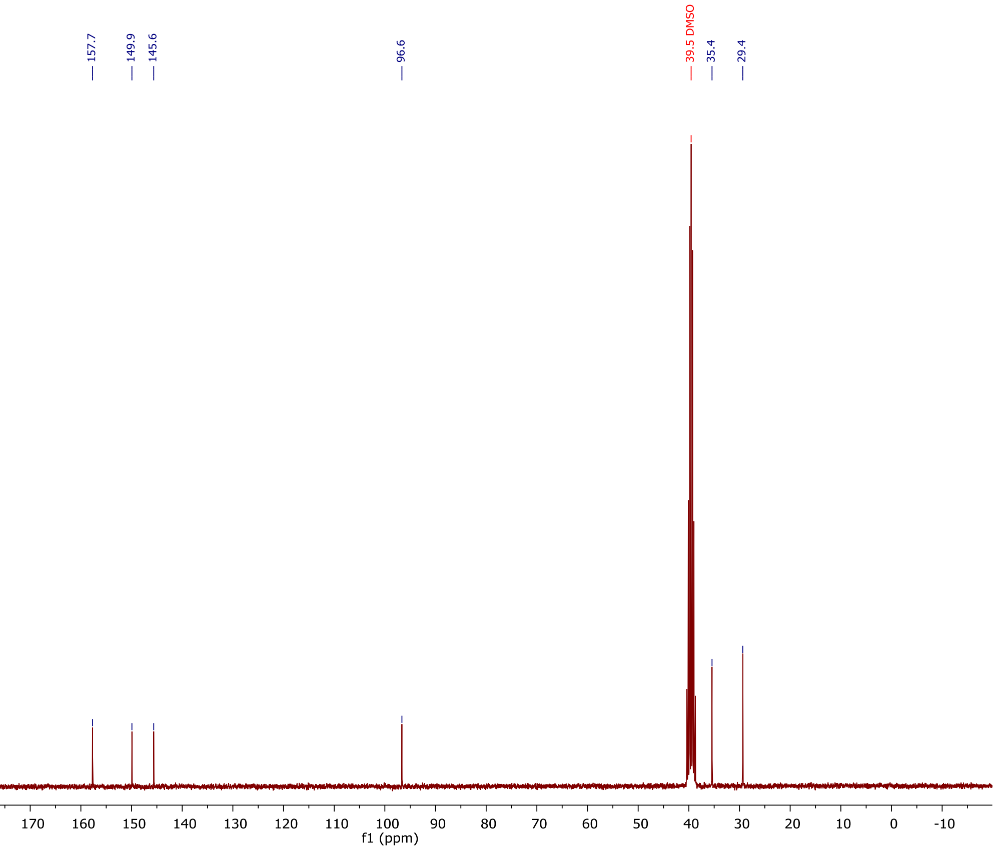

5-Bromo-1,3-dimethyl-6-[2-(4-methylphenyl)ethynyl]uracil (3a)

	Parameter	Value
1	Solvent	CDCl3
2	Temperature	298.2
3	Pulse Sequence	zg30
4	Experiment	1D
5	Number of Scans	16
6	Receiver Gain	406.0
7	Relaxation Delay	2.0000
8	Pulse Width	10.0000
9	Spectrometer Frequency	300.13
10	Spectral Width	6188.1
11	Lowest Frequency	-1267.0
12	Nucleus	1H
13	Acquired Size	32768
14	Spectral Size	65536

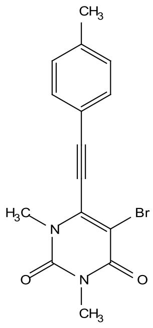

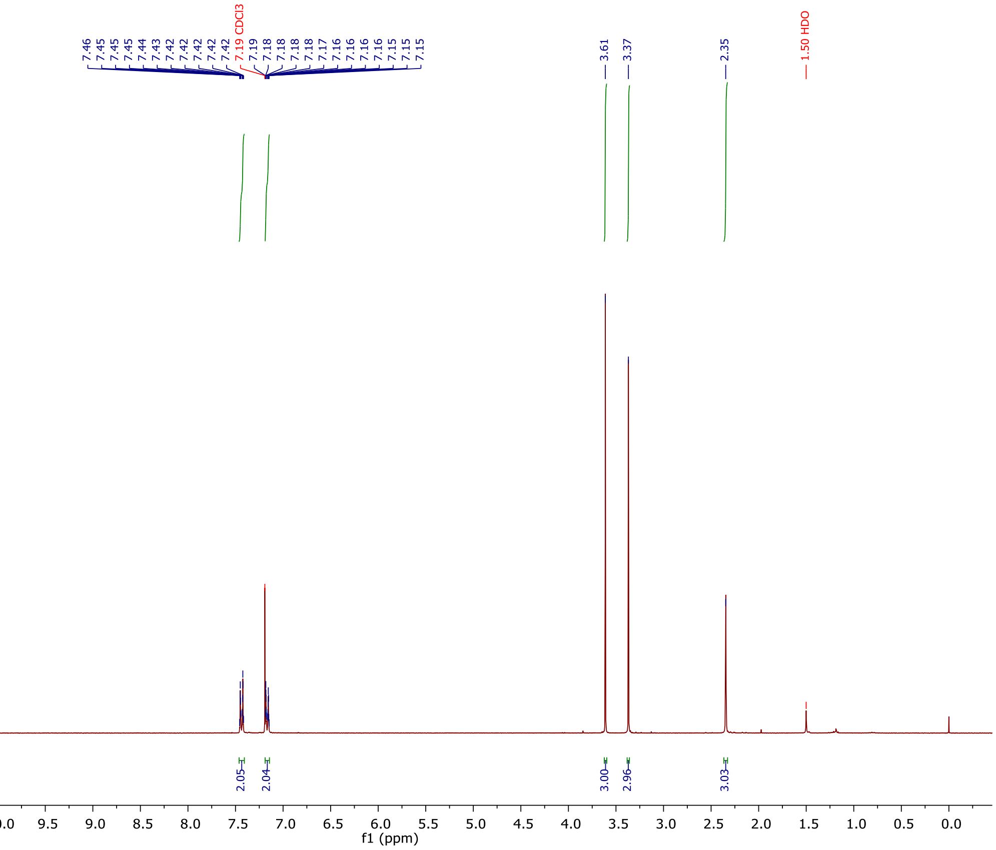

	Parameter	Value
1	Solvent	CDCl3
2	Temperature	298.4
3	Pulse Sequence	zgpg30
4	Experiment	1D
5	Number of Scans	1024
6	Receiver Gain	2050.0
7	Relaxation Delay	2.0000
8	Pulse Width	10.0000
9	Spectrometer Frequency	75.48
10	Spectral Width	18028.8
11	Lowest Frequency	-1468.1
12	Nucleus	13C
13	Acquired Size	32768
14	Spectral Size	65536

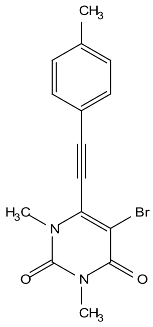

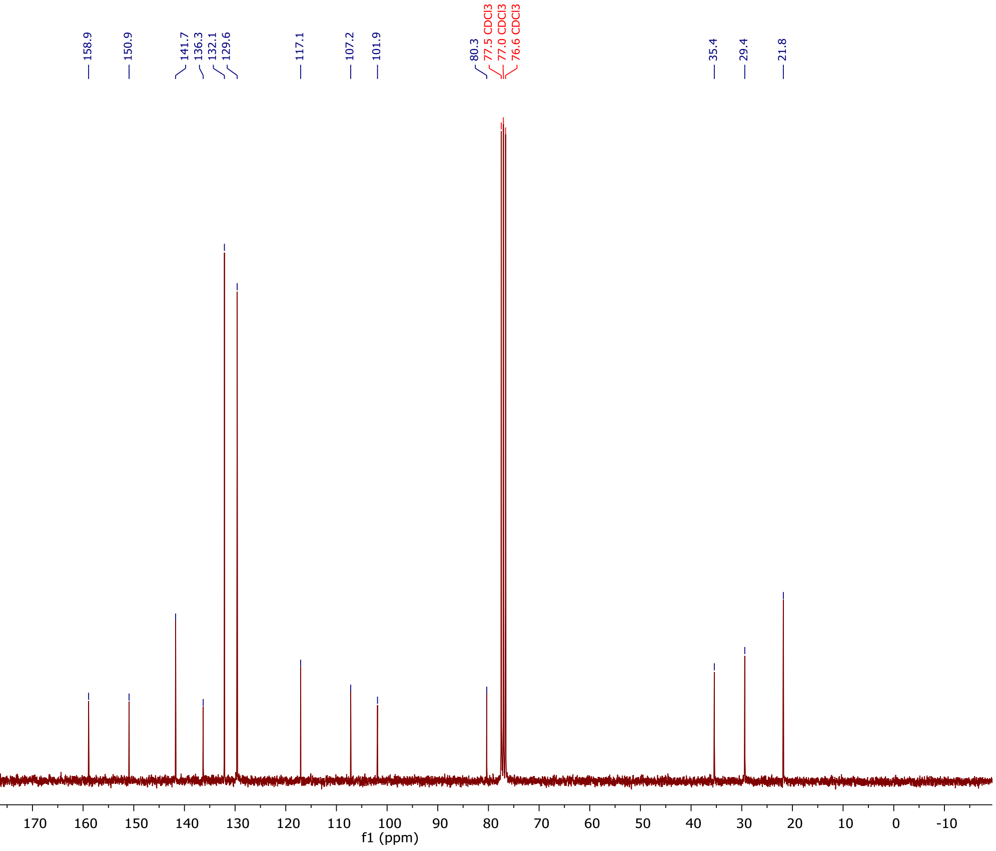

5-Bromo-1,3-dimethyl-6-[2-(phenyl)ethynyl]uracil (3b)

	Parameter	Value
1	Solvent	CDCl3
2	Temperature	298.2
3	Pulse Sequence	zg30
4	Experiment	1D
5	Number of Scans	16
6	Receiver Gain	322.0
7	Relaxation Delay	2.0000
8	Pulse Width	10.0000
9	Spectrometer Frequency	300.13
10	Spectral Width	6188.1
11	Lowest Frequency	-1246.5
12	Nucleus	1H
13	Acquired Size	32768
14	Spectral Size	65536

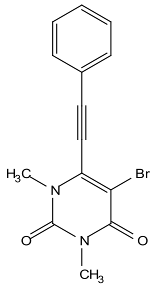

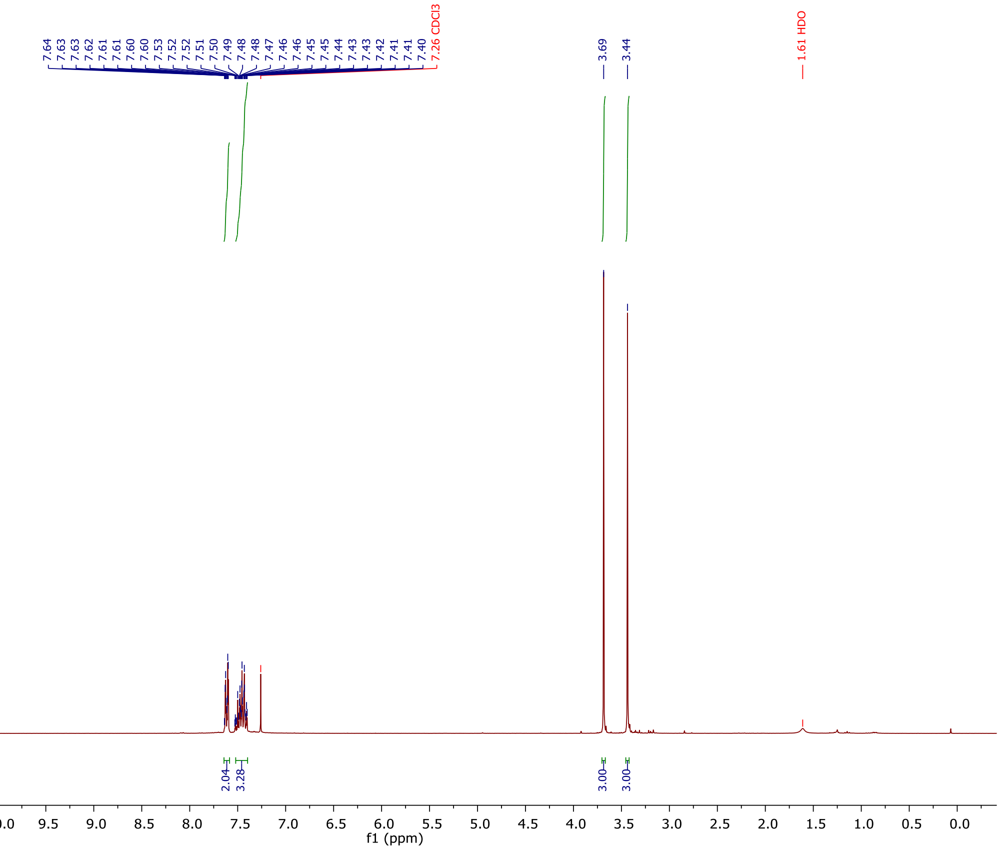

	Parameter	Value
1	Solvent	CDCl3
2	Temperature	298.9
3	Pulse Sequence	zgpg30
4	Experiment	1D
5	Number of Scans	1024
6	Receiver Gain	2050.0
7	Relaxation Delay	2.0000
8	Pulse Width	10.0000
9	Spectrometer Frequency	75.48
10	Spectral Width	18028.8
11	Lowest Frequency	-1457.9
12	Nucleus	13C
13	Acquired Size	32768
14	Spectral Size	65536

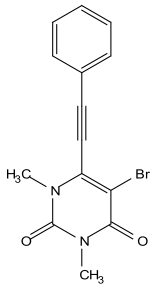

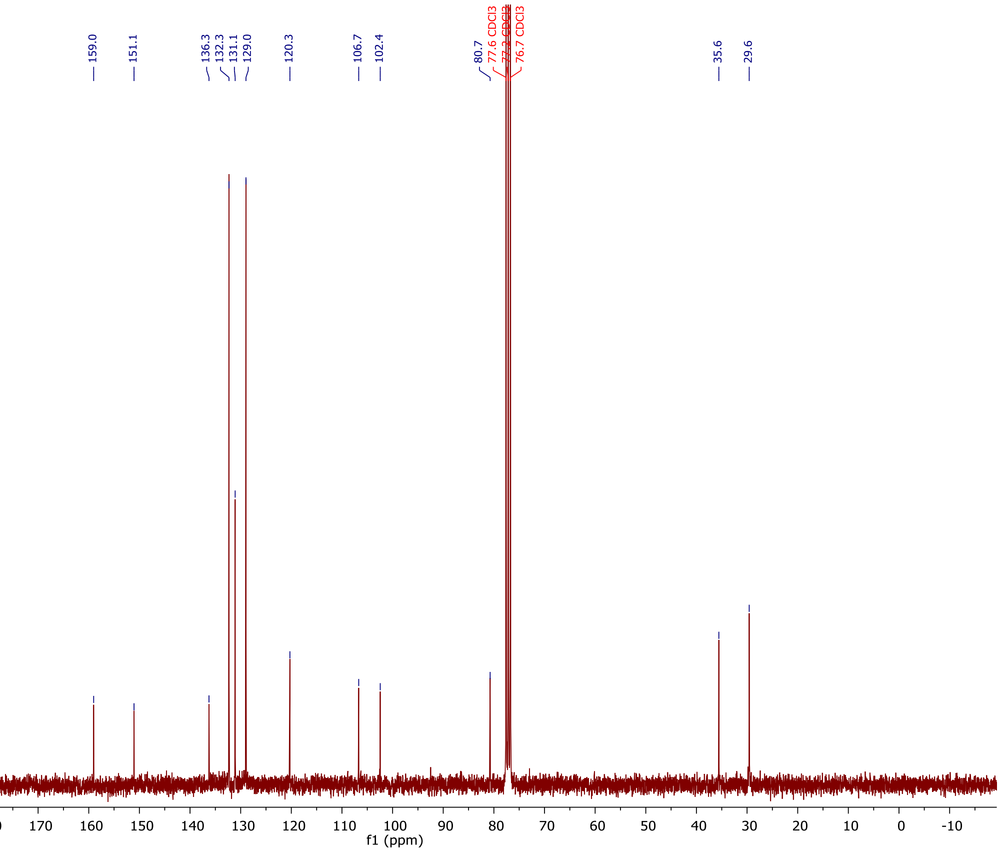

5-Bromo-1,3-dimethyl-6-[2-(4-fluorophenyl)ethynyl]uracil (3c)

	Parameter	Value
1	Solvent	CDCl3
2	Temperature	298.2
3	Pulse Sequence	zg30
4	Experiment	1D
5	Number of Scans	16
6	Receiver Gain	287.0
7	Relaxation Delay	2.0000
8	Pulse Width	10.0000
9	Spectrometer Frequency	300.13
10	Spectral Width	6188.1
11	Lowest Frequency	-1246.6
12	Nucleus	1H
13	Acquired Size	32768
14	Spectral Size	65536

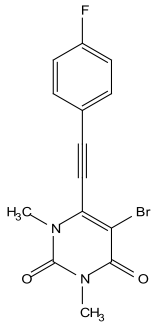

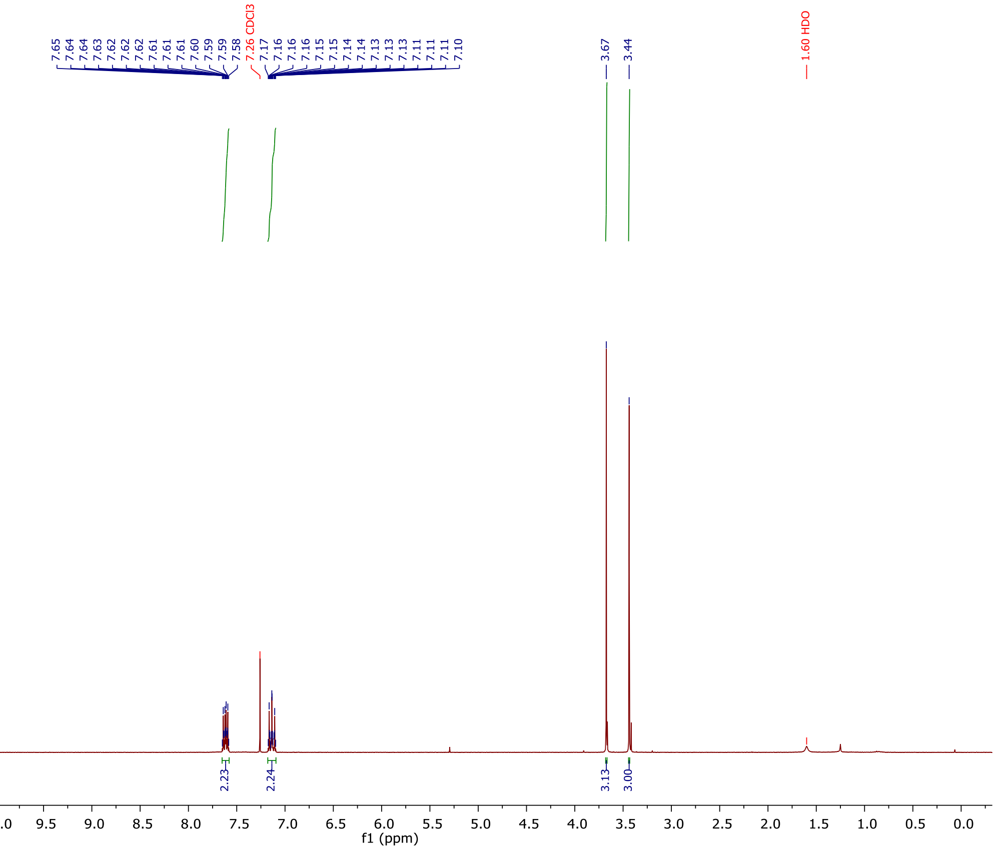

	Parameter	Value
1	Solvent	CDCl3
2	Temperature	298.2
3	Pulse Sequence	zgfhigqn
4	Experiment	1D
5	Number of Scans	64
6	Receiver Gain	2050.0
7	Relaxation Delay	1.0000
8	Pulse Width	10.0000
9	Spectrometer Frequency	282.38
10	Spectral Width	66964.3
11	Lowest Frequency	-61722.4
12	Nucleus	19F
13	Acquired Size	65536
14	Spectral Size	131072

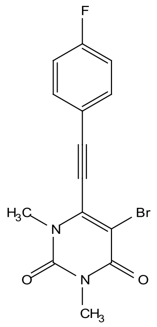

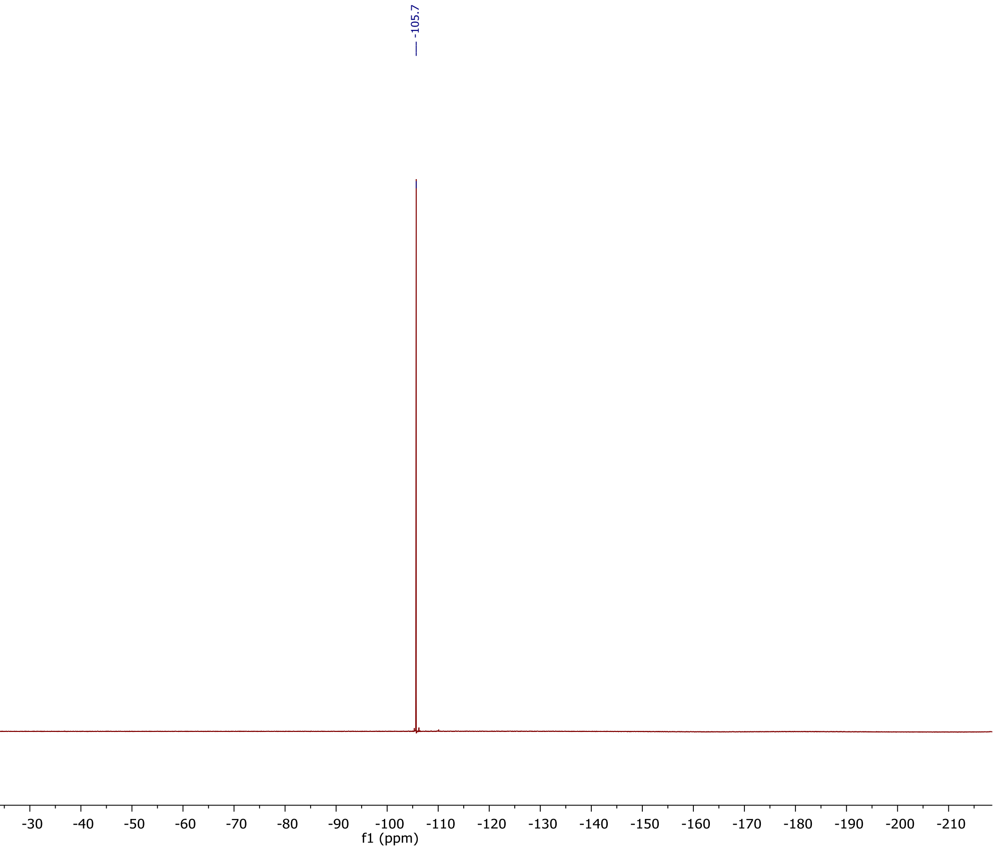

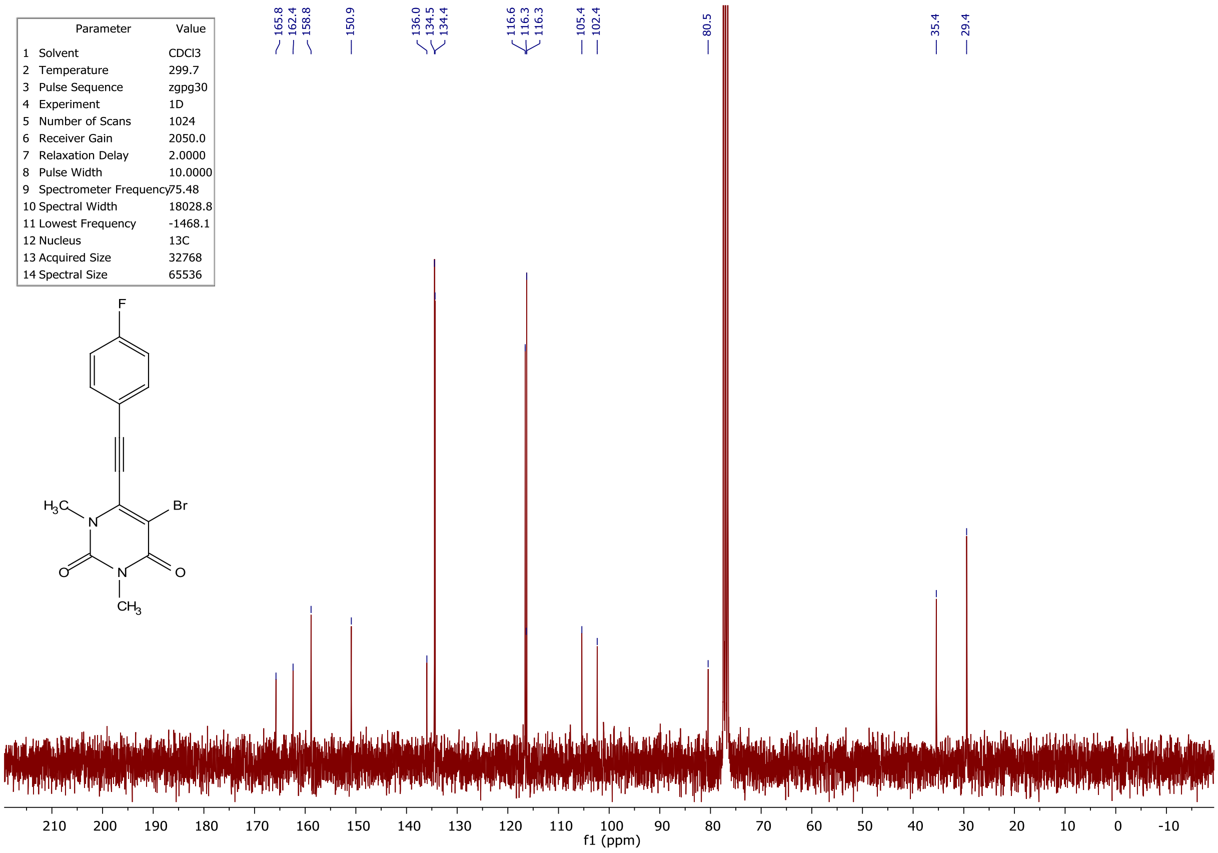

	Parameter	Value
1	Solvent	CDCl3
2	Temperature	299.7
3	Pulse Sequence	zgpg30
4	Experiment	1D
5	Number of Scans	1024
6	Receiver Gain	2050.0
7	Relaxation Delay	2.0000
8	Pulse Width	10.0000
9	Spectrometer Frequency	75.48
10	Spectral Width	18028.8
11	Lowest Frequency	-1468.1
12	Nucleus	13C
13	Acquired Size	32768
14	Spectral Size	65536

5-Bromo-1,3-dimethyl-6-[2-(4-*tert*-butylphenyl)ethynyl]uracil (3d)

Parameter		Value
1	Solvent	CDCl3
2	Temperature	298.2
3	Pulse Sequence	zg30
4	Experiment	1D
5	Number of Scans	16
6	Receiver Gain	90.5
7	Relaxation Delay	2.0000
8	Pulse Width	10.0000
9	Spectrometer Frequency	300.13
10	Spectral Width	6188.1
11	Lowest Frequency	-1246.3
12	Nucleus	1H
13	Acquired Size	32768
14	Spectral Size	65536

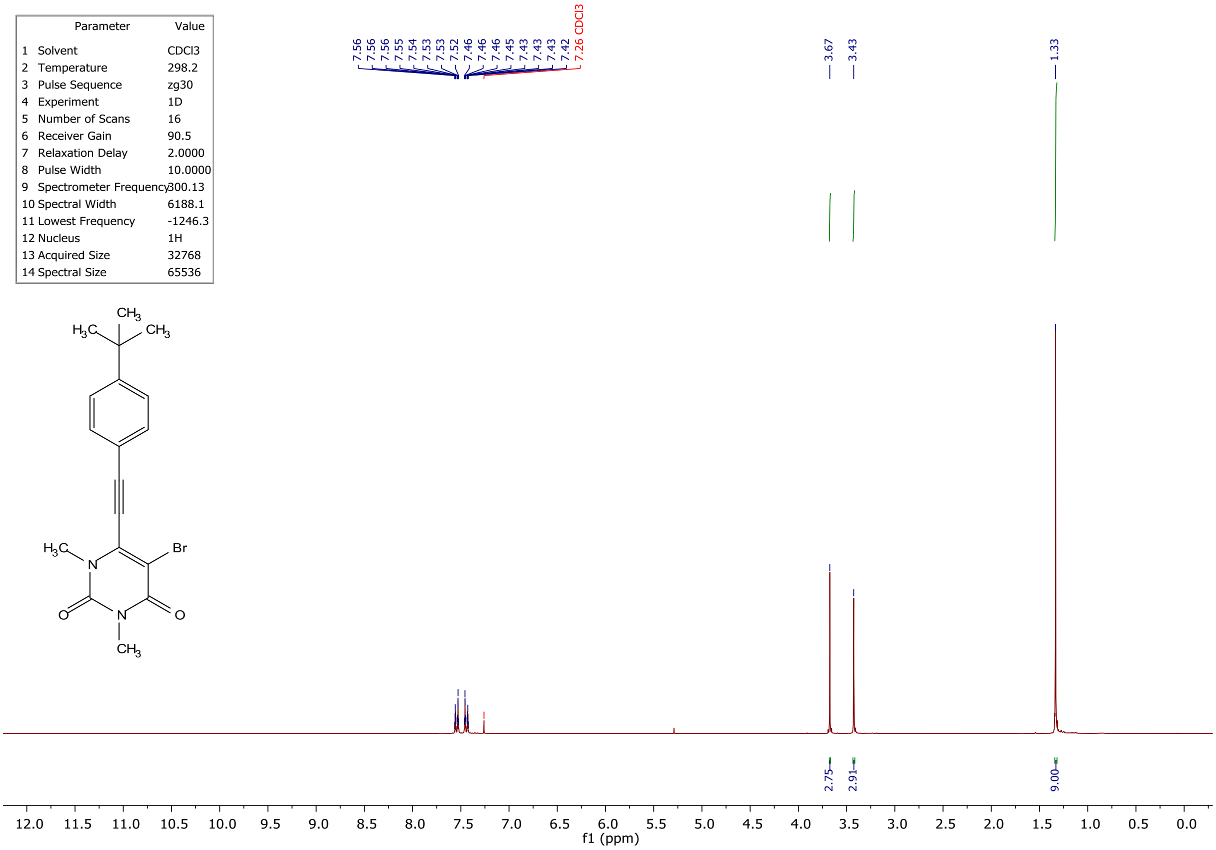

Parameter		Value
1	Solvent	CDCl3
2	Temperature	298.2
3	Pulse Sequence	zgpg30
4	Experiment	1D
5	Number of Scans	1024
6	Receiver Gain	2050.0
7	Relaxation Delay	2.0000
8	Pulse Width	10.0000
9	Spectrometer Frequency	75.48
10	Spectral Width	18028.8
11	Lowest Frequency	-1459.4
12	Nucleus	13C
13	Acquired Size	32768
14	Spectral Size	65536

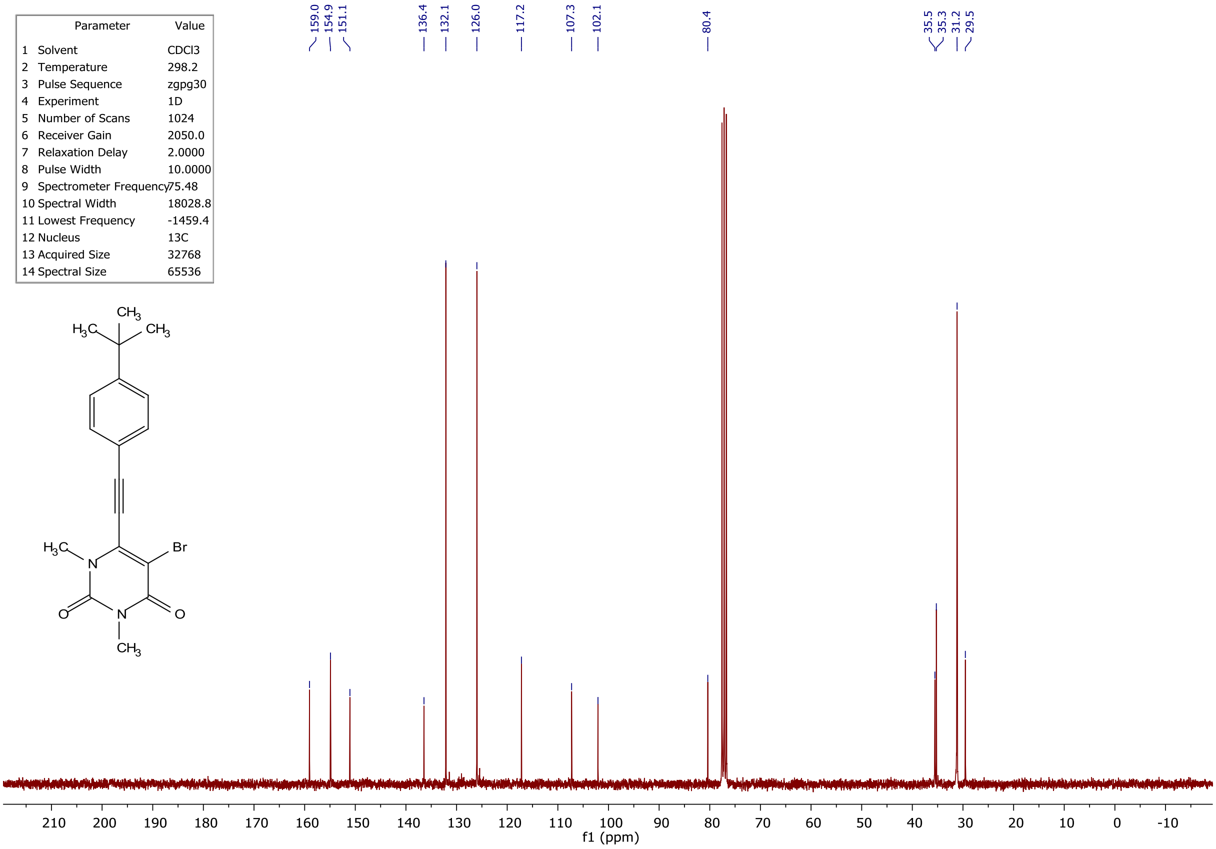

5-Bromo-1,3-dimethyl-6-[2-(4-trifluoromethylphenyl)ethynyl]uracil (3e)

	Parameter	Value
1	Solvent	CDCl3
2	Temperature	298.2
3	Pulse Sequence	zg30
4	Experiment	1D
5	Number of Scans	16
6	Receiver Gain	456.0
7	Relaxation Delay	2.0000
8	Pulse Width	10.0000
9	Spectrometer Frequency	300.13
10	Spectral Width	6188.1
11	Lowest Frequency	-1246.4
12	Nucleus	1H
13	Acquired Size	32768
14	Spectral Size	65536

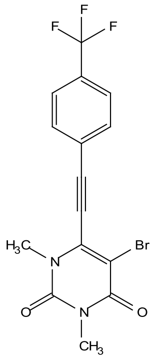

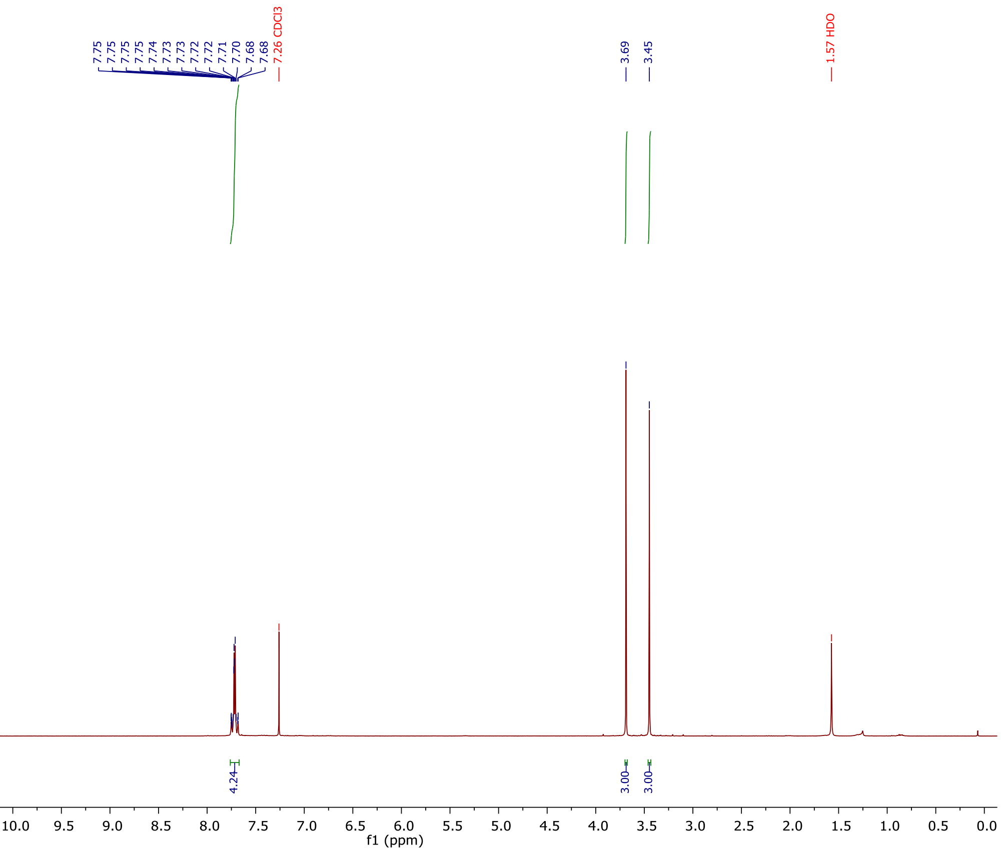

	Parameter	Value
1	Solvent	CDCl3
2	Temperature	298.2
3	Pulse Sequence	zgfhigqn
4	Experiment	1D
5	Number of Scans	64
6	Receiver Gain	2050.0
7	Relaxation Delay	1.0000
8	Pulse Width	10.0000
9	Spectrometer Frequency	282.38
10	Spectral Width	66964.3
11	Lowest Frequency	-61722.4
12	Nucleus	19F
13	Acquired Size	65536
14	Spectral Size	131072

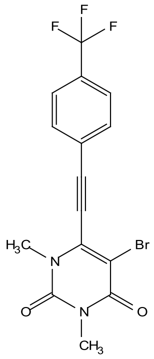

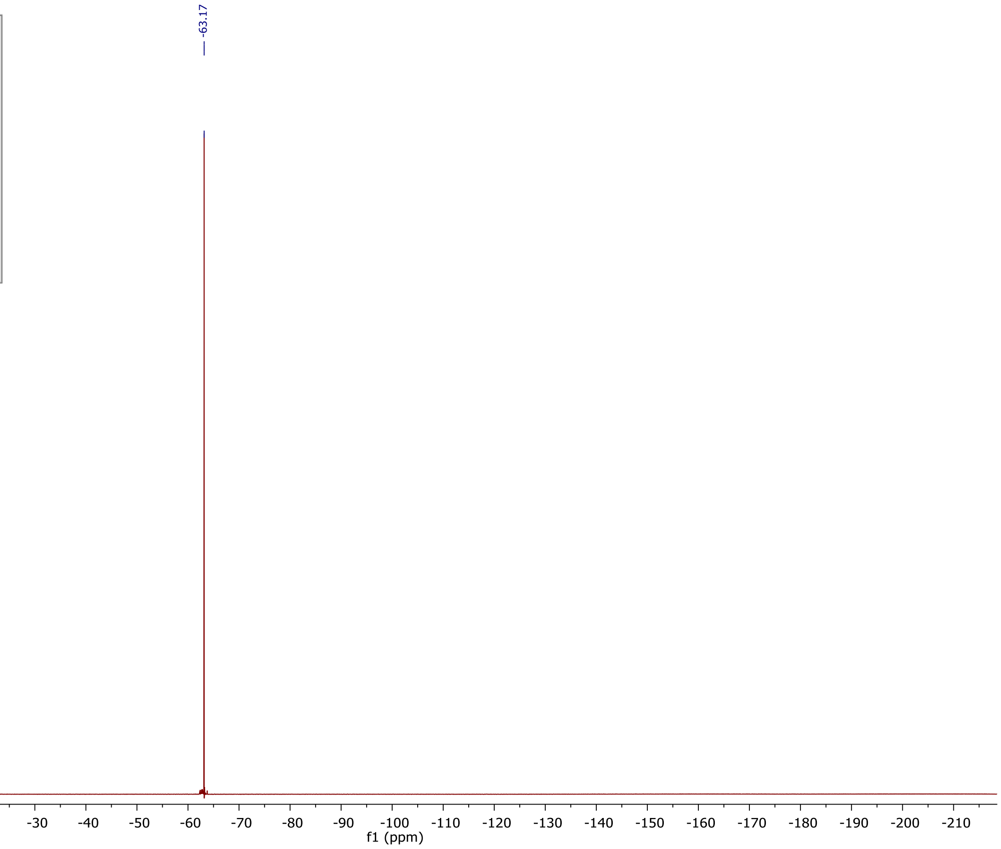

	Parameter	Value
1	Solvent	CDCl3
2	Temperature	298.1
3	Pulse Sequence	zgpg30
4	Experiment	1D
5	Number of Scans	5120
6	Receiver Gain	101.0
7	Relaxation Delay	2.0000
8	Pulse Width	10.0000
9	Spectrometer Frequency	125.77
10	Spectral Width	30120.5
11	Lowest Frequency	-2468.1
12	Nucleus	13C
13	Acquired Size	32768
14	Spectral Size	65536

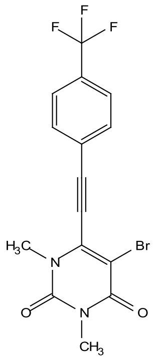

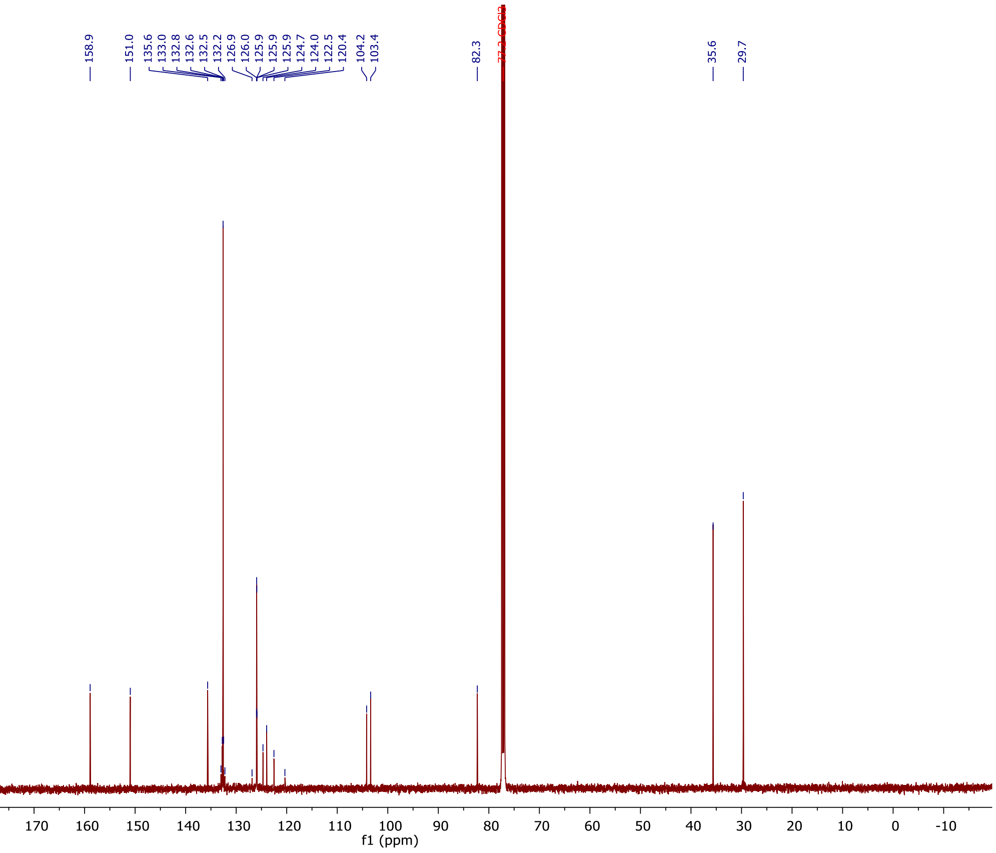

5-Bromo-1,3-dimethyl-6-[2-(4-methoxyphenyl)ethynyl]uracil (3f)

	Parameter	Value
1	Solvent	CDCl3
2	Temperature	298.7
3	Pulse Sequence	zg30
4	Experiment	1D
5	Number of Scans	16
6	Receiver Gain	287.0
7	Relaxation Delay	2.0000
8	Pulse Width	10.0000
9	Spectrometer Frequency	300.13
10	Spectral Width	6188.1
11	Lowest Frequency	-1246.6
12	Nucleus	1H
13	Acquired Size	32768
14	Spectral Size	65536

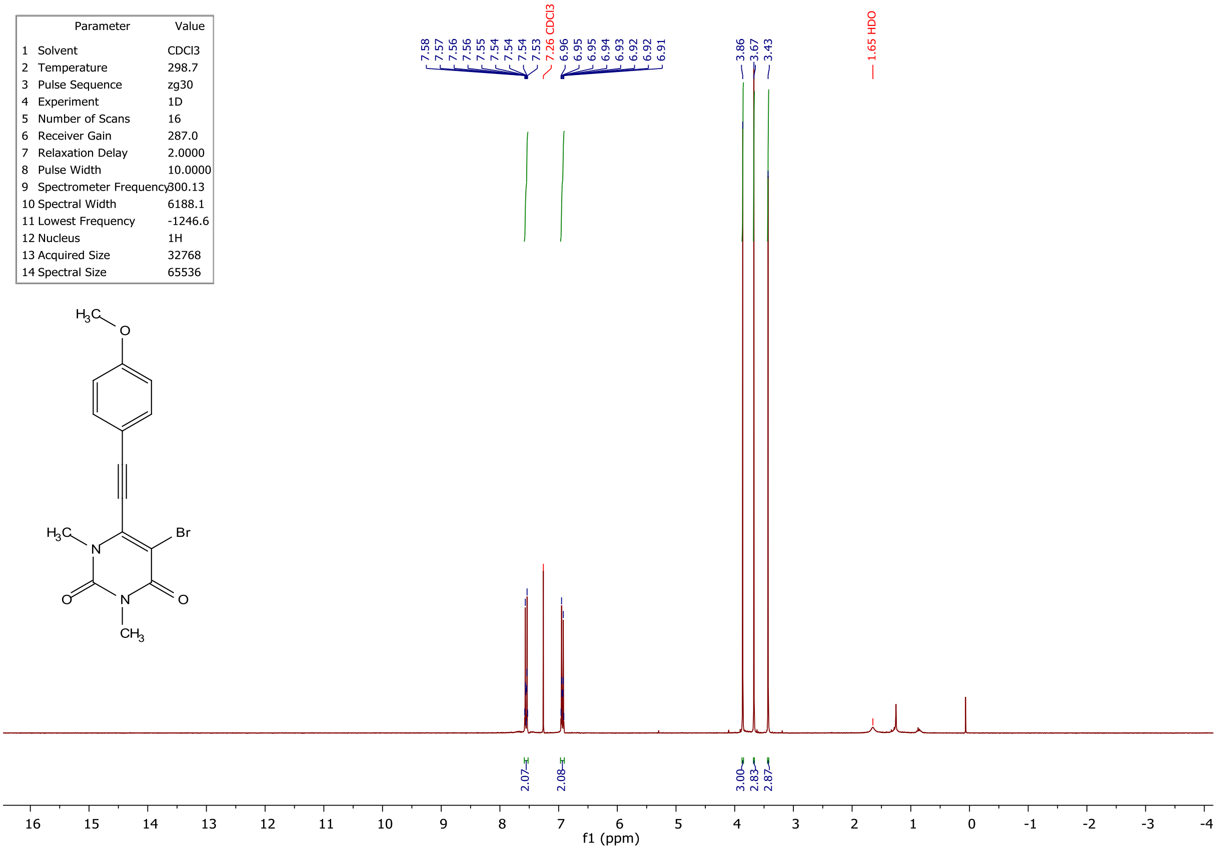

	Parameter	Value
1	Solvent	CDCl3
2	Temperature	299.3
3	Pulse Sequence	zgpg30
4	Experiment	1D
5	Number of Scans	1024
6	Receiver Gain	2050.0
7	Relaxation Delay	2.0000
8	Pulse Width	10.0000
9	Spectrometer Frequency	75.48
10	Spectral Width	18028.8
11	Lowest Frequency	-1457.2
12	Nucleus	13C
13	Acquired Size	32768
14	Spectral Size	65536

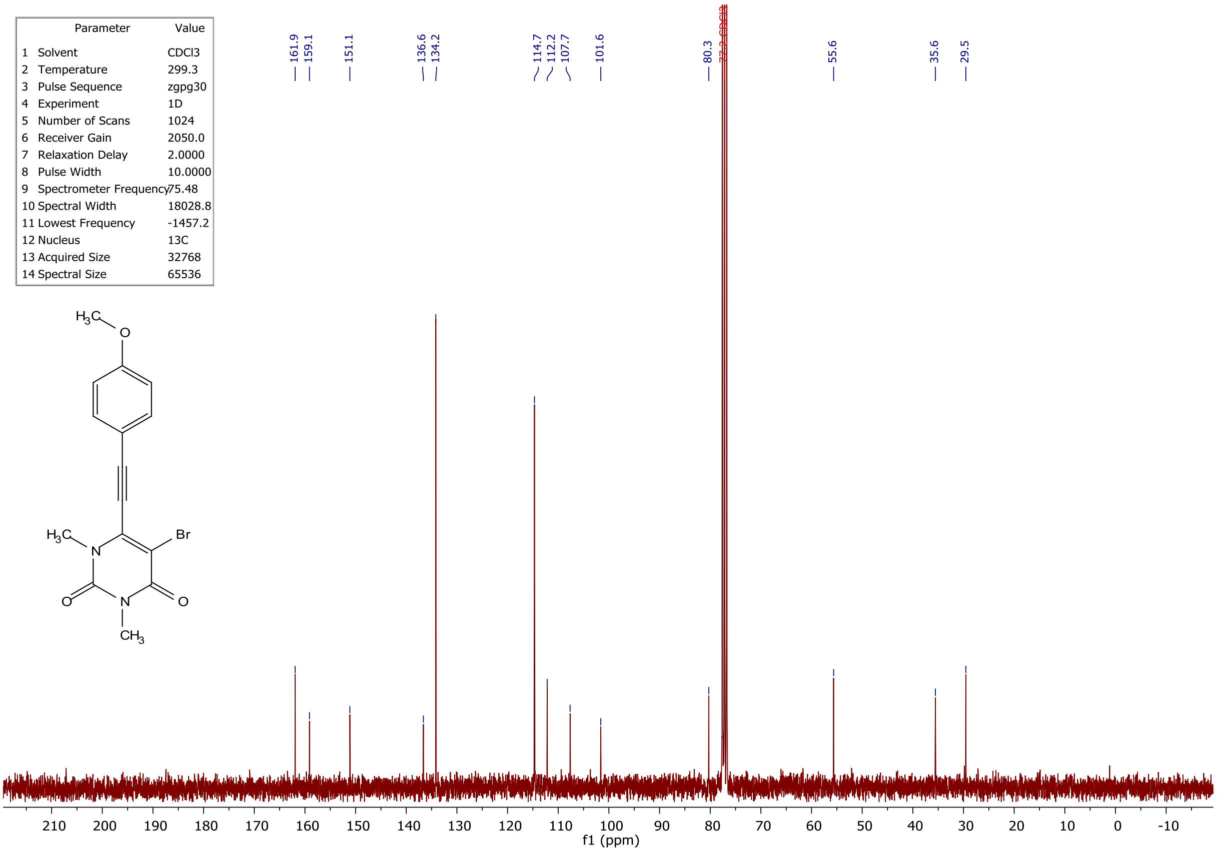

5-Bromo-1,3-dimethyl-6-[2-(thiophene)ethynyl]uracil (3h)

	Parameter	Value
1	Solvent	CDCl3
2	Temperature	298.2
3	Pulse Sequence	zg30
4	Experiment	1D
5	Number of Scans	16
6	Receiver Gain	362.0
7	Relaxation Delay	2.0000
8	Pulse Width	10.0000
9	Spectrometer Frequency	300.13
10	Spectral Width	6188.1
11	Lowest Frequency	-1246.4
12	Nucleus	1H
13	Acquired Size	32768
14	Spectral Size	65536

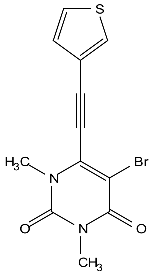

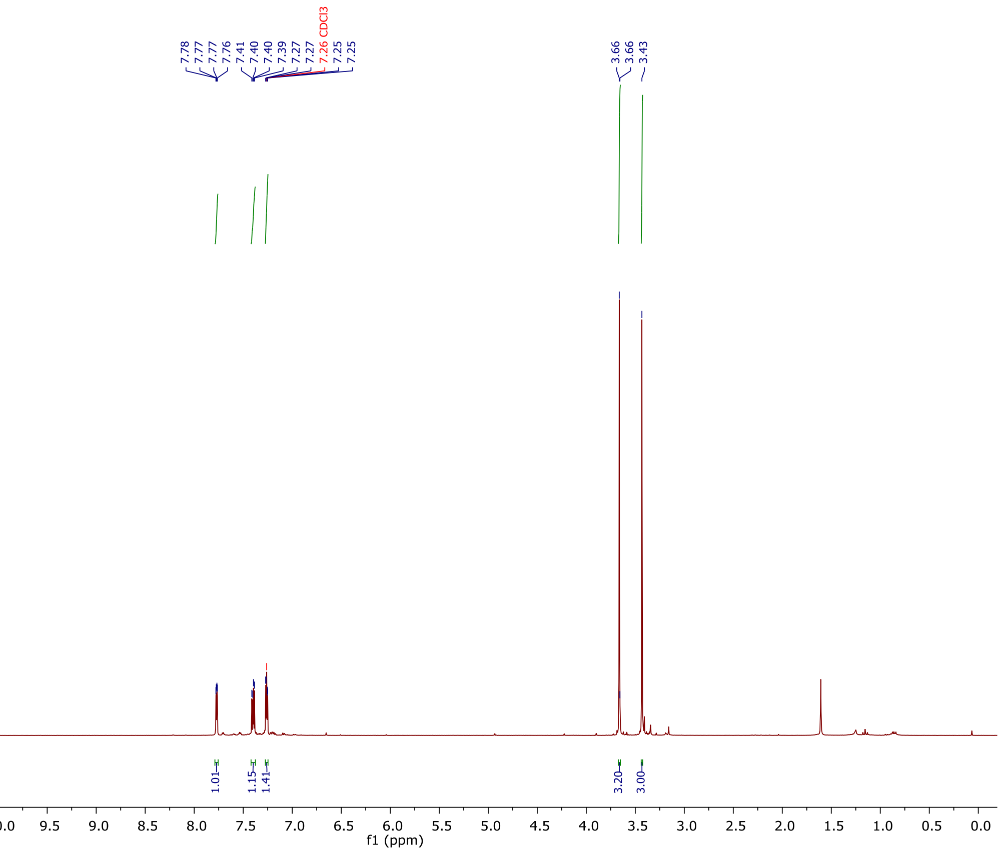

	Parameter	Value
1	Solvent	CDCl3
2	Temperature	298.9
3	Pulse Sequence	zgpg30
4	Experiment	1D
5	Number of Scans	1024
6	Receiver Gain	2050.0
7	Relaxation Delay	2.0000
8	Pulse Width	10.0000
9	Spectrometer Frequency	75.48
10	Spectral Width	18028.8
11	Lowest Frequency	-1457.9
12	Nucleus	13C
13	Acquired Size	32768
14	Spectral Size	65536

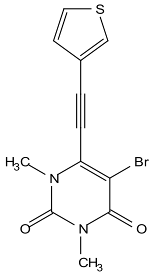

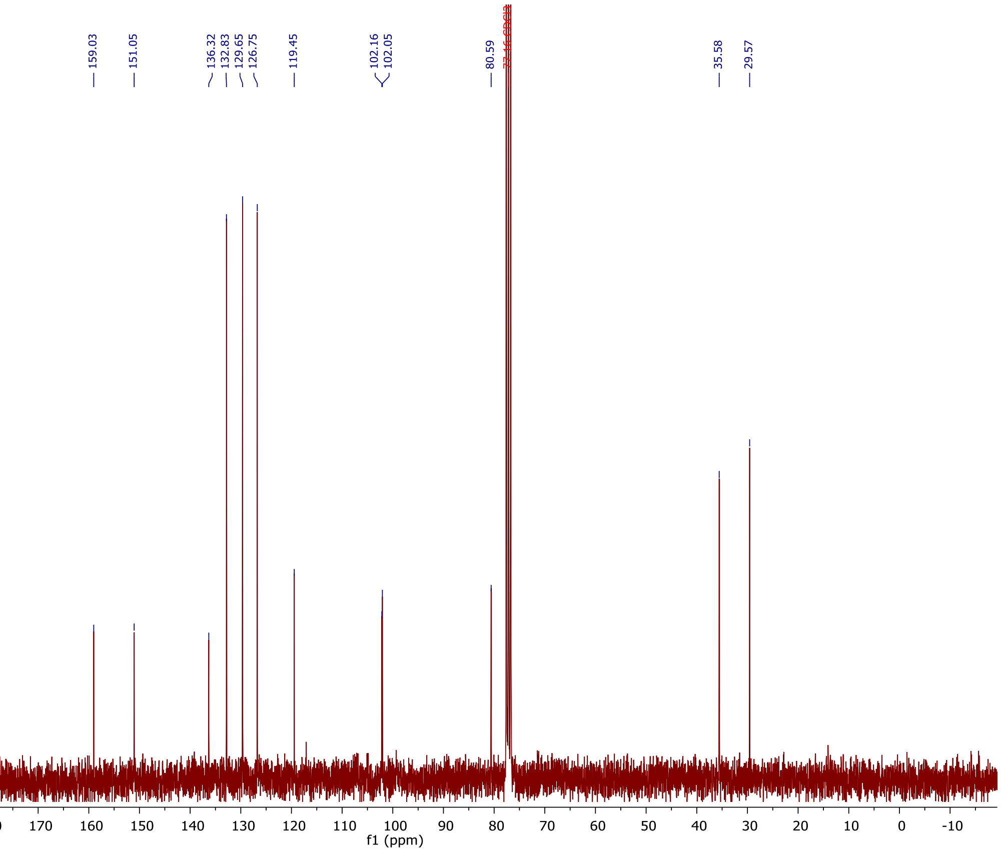

5-Bromo-1,3-dimethyl-6-[2-(4-dimehtylamino)ethynyl]uracil (3i)

	Parameter	Value
1	Solvent	CDCl3
2	Temperature	298.2
3	Pulse Sequence	zgpg30
4	Experiment	1D
5	Number of Scans	1024
6	Receiver Gain	101.0
7	Relaxation Delay	2.0000
8	Pulse Width	10.0000
9	Spectrometer Frequency	125.77
10	Spectral Width	30120.5
11	Lowest Frequency	-2472.3
12	Nucleus	13C
13	Acquired Size	32768
14	Spectral Size	65536

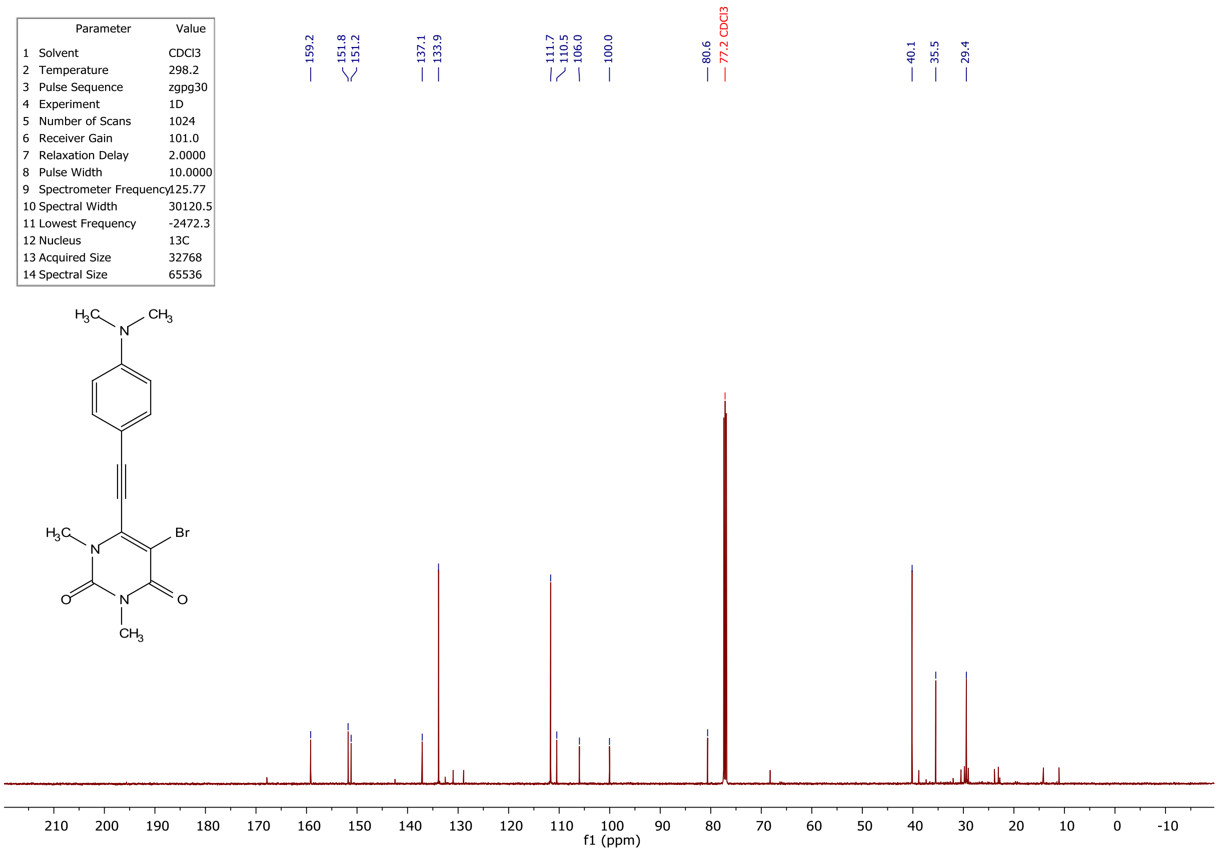

	Parameter	Value
1	Solvent	CDCl3
2	Temperature	298.1
3	Pulse Sequence	zg30
4	Experiment	1D
5	Number of Scans	16
6	Receiver Gain	101.0
7	Relaxation Delay	1.0000
8	Pulse Width	8.0000
9	Spectrometer Frequency	500.13
10	Spectral Width	10000.0
11	Lowest Frequency	-1923.8
12	Nucleus	1H
13	Acquired Size	32768
14	Spectral Size	65536

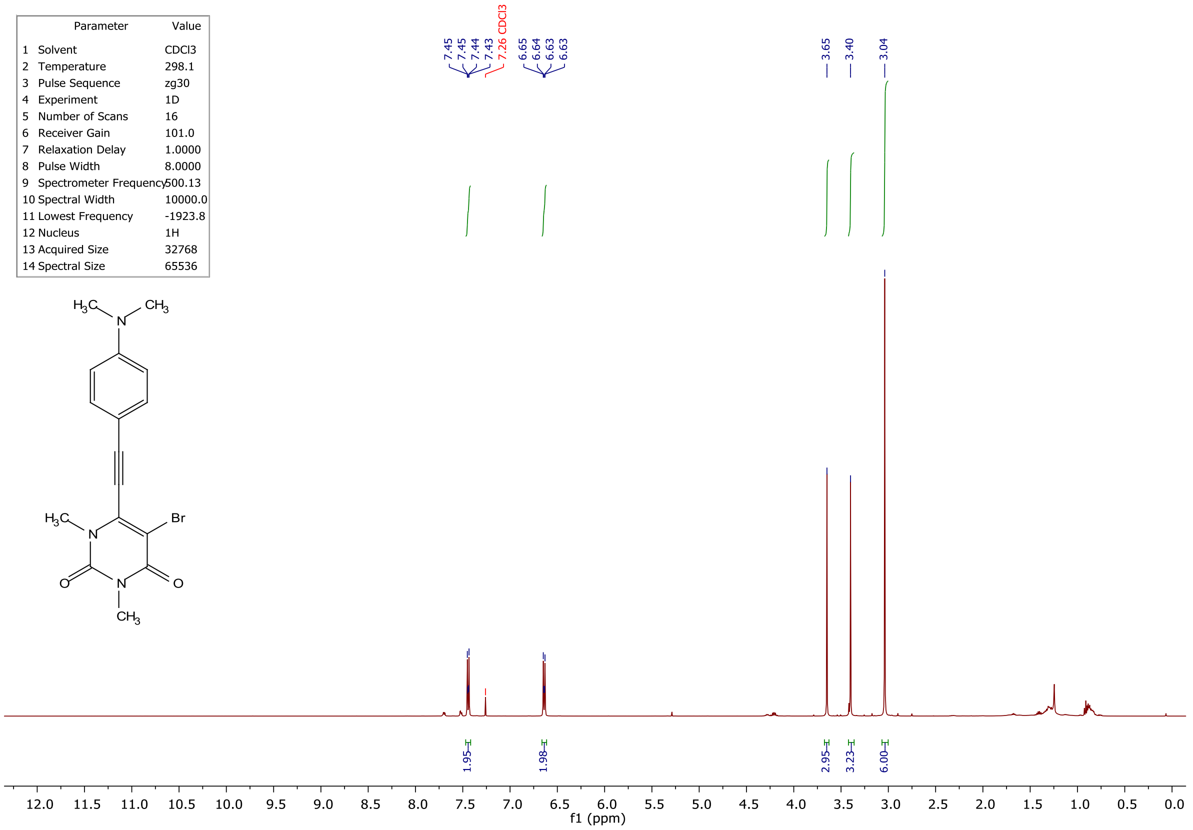

5-Bromo-1,3-dimethyl-6-[2-(biphenyl)ethynyl]uracil (3j)

Parameter	Value
1 Solvent	CDCl3
2 Temperature	298.2
3 Pulse Sequence	zg30
4 Experiment	1D
5 Number of Scans	16
6 Receiver Gain	144.0
7 Relaxation Delay	2.0000
8 Pulse Width	10.0000
9 Spectrometer Frequency	300.13
10 Spectral Width	6188.1
11 Lowest Frequency	-1246.3
12 Nucleus	1H
13 Acquired Size	32768
14 Spectral Size	65536

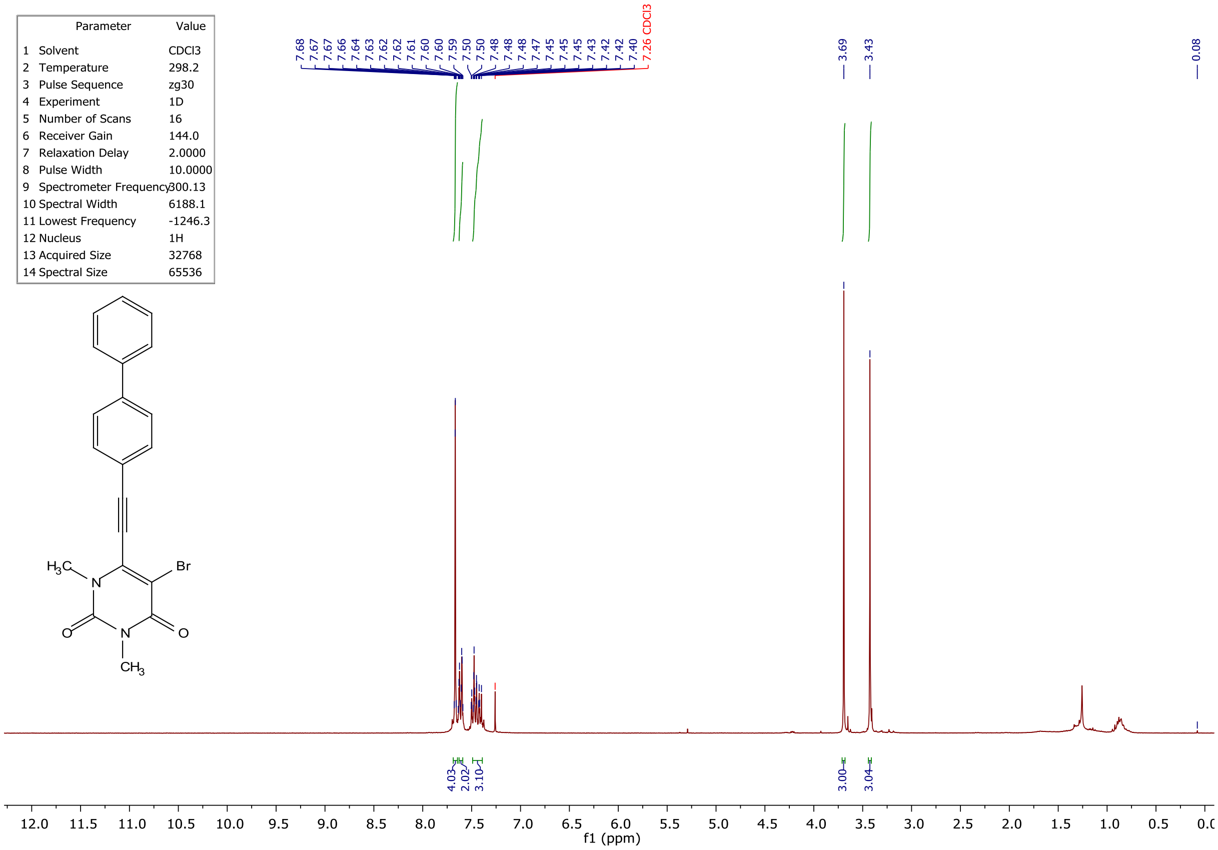

Parameter	Value
1 Solvent	CDCl3
2 Temperature	298.2
3 Pulse Sequence	zgpg30
4 Experiment	1D
5 Number of Scans	1024
6 Receiver Gain	2050.0
7 Relaxation Delay	2.0000
8 Pulse Width	10.0000
9 Spectrometer Frequency	75.48
10 Spectral Width	18028.8
11 Lowest Frequency	-1459.7
12 Nucleus	13C
13 Acquired Size	32768
14 Spectral Size	65536

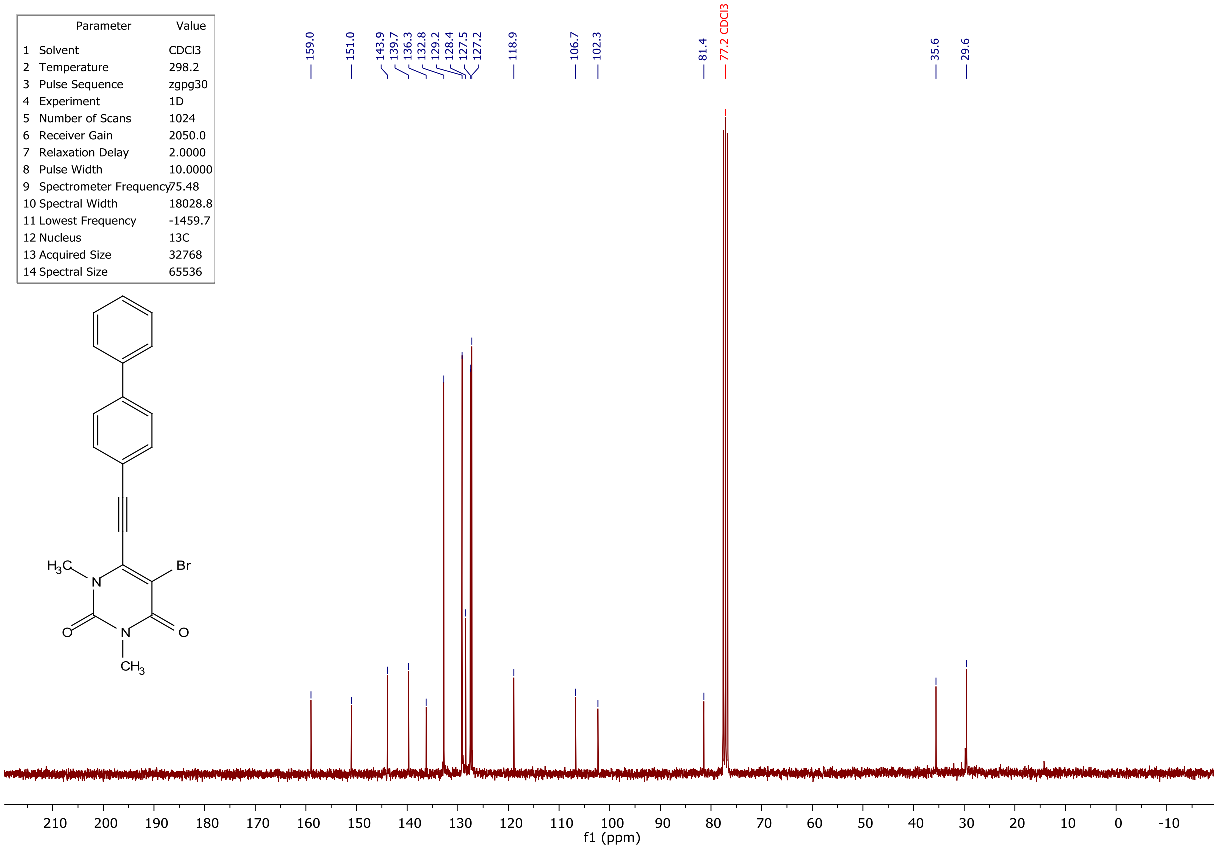

1,3-Dimethyl-5,6-[2-(4-methylphenyl)ethynyl]uracil (4a)

	Parameter	Value
1	Solvent	CDCl3
2	Temperature	298.2
3	Pulse Sequence	zg30
4	Experiment	1D
5	Number of Scans	16
6	Receiver Gain	161.0
7	Relaxation Delay	2.0000
8	Pulse Width	10.0000
9	Spectrometer Frequency	300.13
10	Spectral Width	6188.1
11	Lowest Frequency	-1246.3
12	Nucleus	1H
13	Acquired Size	32768
14	Spectral Size	65536

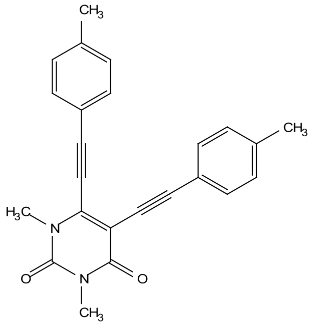

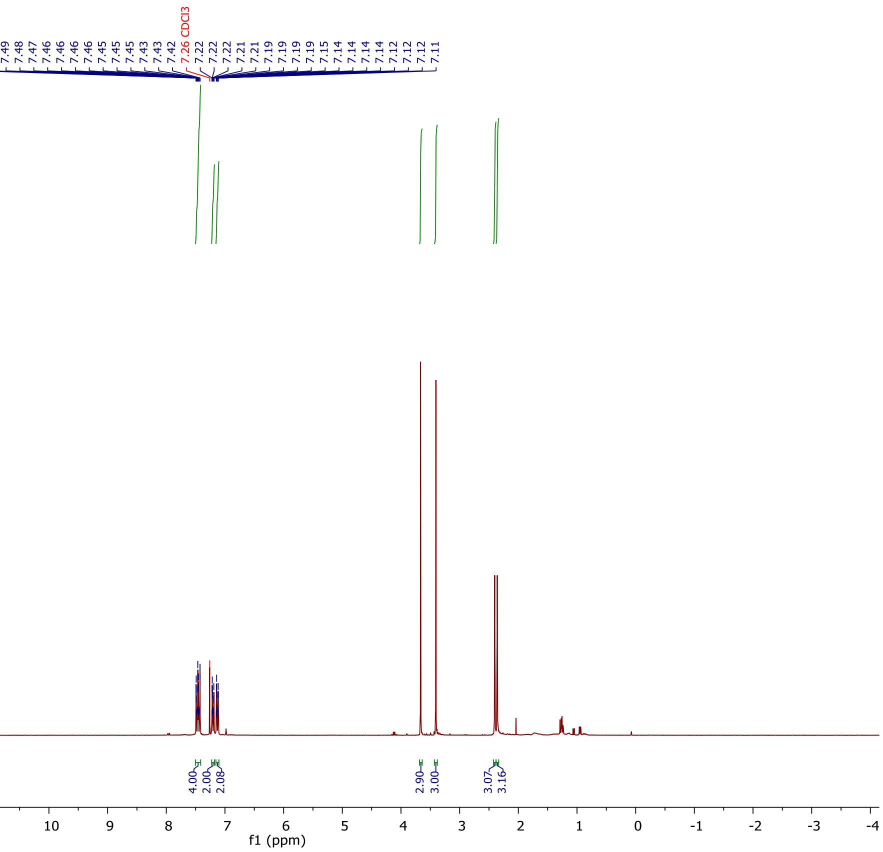

	Parameter	Value
1	Solvent	CDCl3
2	Temperature	298.5
3	Pulse Sequence	zgpg30
4	Experiment	1D
5	Number of Scans	1024
6	Receiver Gain	2050.0
7	Relaxation Delay	2.0000
8	Pulse Width	10.0000
9	Spectrometer Frequency	75.48
10	Spectral Width	18028.8
11	Lowest Frequency	-1457.4
12	Nucleus	13C
13	Acquired Size	32768
14	Spectral Size	65536

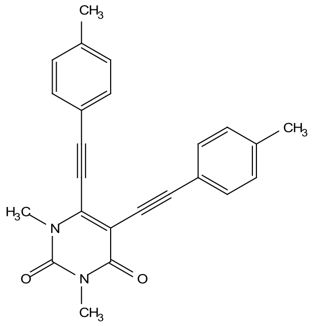

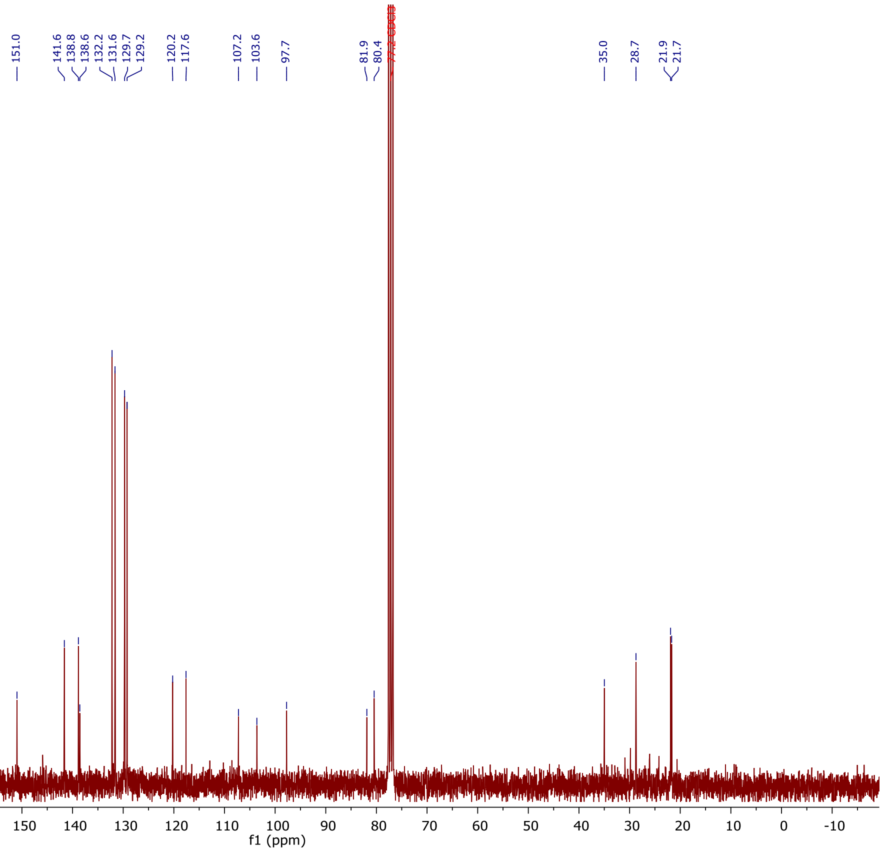

1,3-Dimethyl-5,6-[2-(4-*tert*-butylphenyl)ethynyl]uracil (4b)

	Parameter	Value
1	Solvent	CDCl3
2	Temperature	298.2
3	Pulse Sequence	zg30
4	Experiment	1D
5	Number of Scans	16
6	Receiver Gain	90.5
7	Relaxation Delay	1.0000
8	Pulse Width	8.0000
9	Spectrometer Frequency	500.13
10	Spectral Width	10000.0
11	Lowest Frequency	-1924.7
12	Nucleus	1H
13	Acquired Size	32768
14	Spectral Size	65536

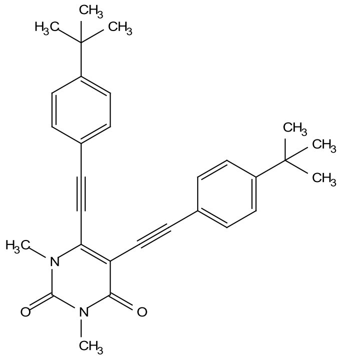

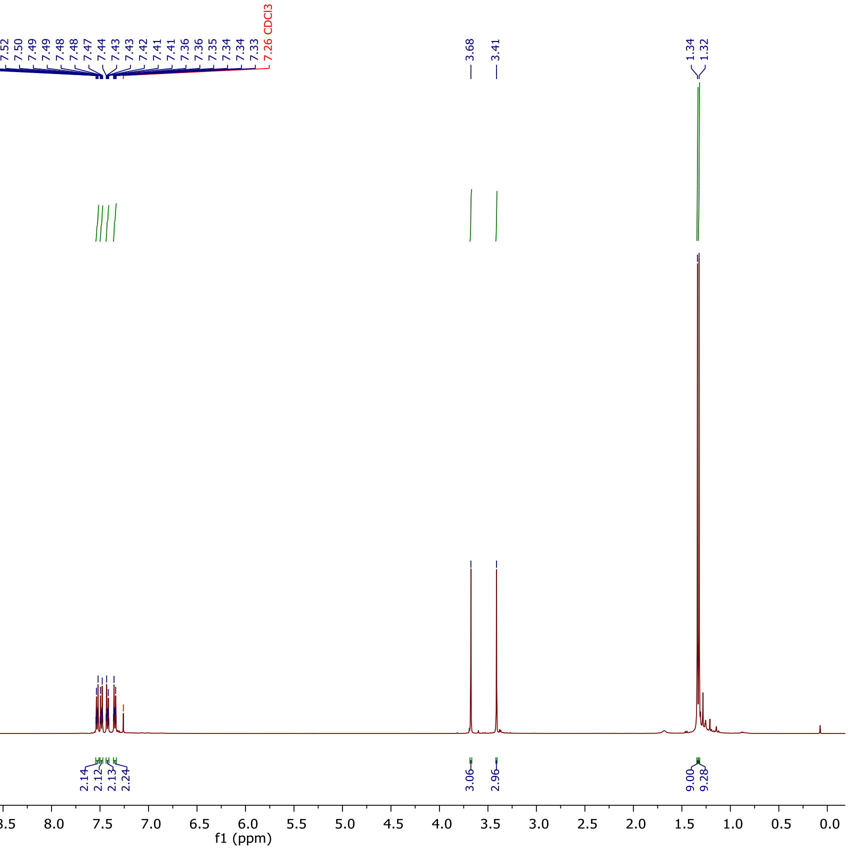

	Parameter	Value
1	Solvent	CDCl3
2	Temperature	298.1
3	Pulse Sequence	zgpg30
4	Experiment	1D
5	Number of Scans	1024
6	Receiver Gain	101.0
7	Relaxation Delay	2.0000
8	Pulse Width	10.0000
9	Spectrometer Frequency	125.77
10	Spectral Width	30120.5
11	Lowest Frequency	-2471.0
12	Nucleus	13C
13	Acquired Size	32768
14	Spectral Size	65536

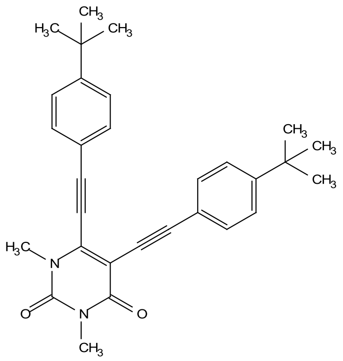

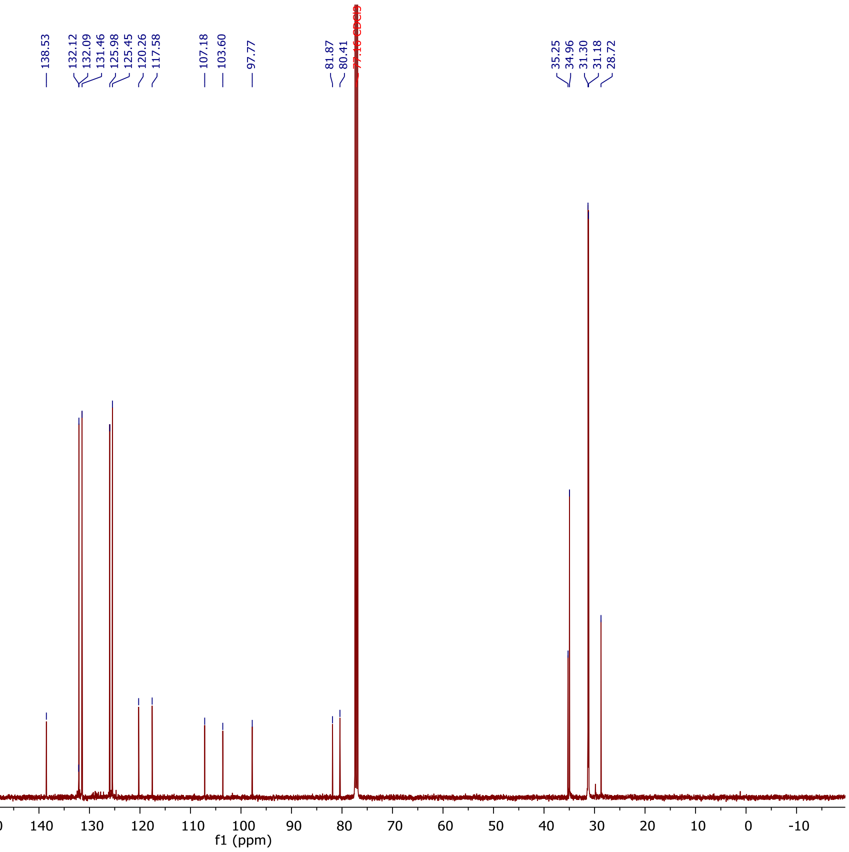

1,3-Dimethyl-5,6-[2-(phenyl)ethynyl]uracil (4c)

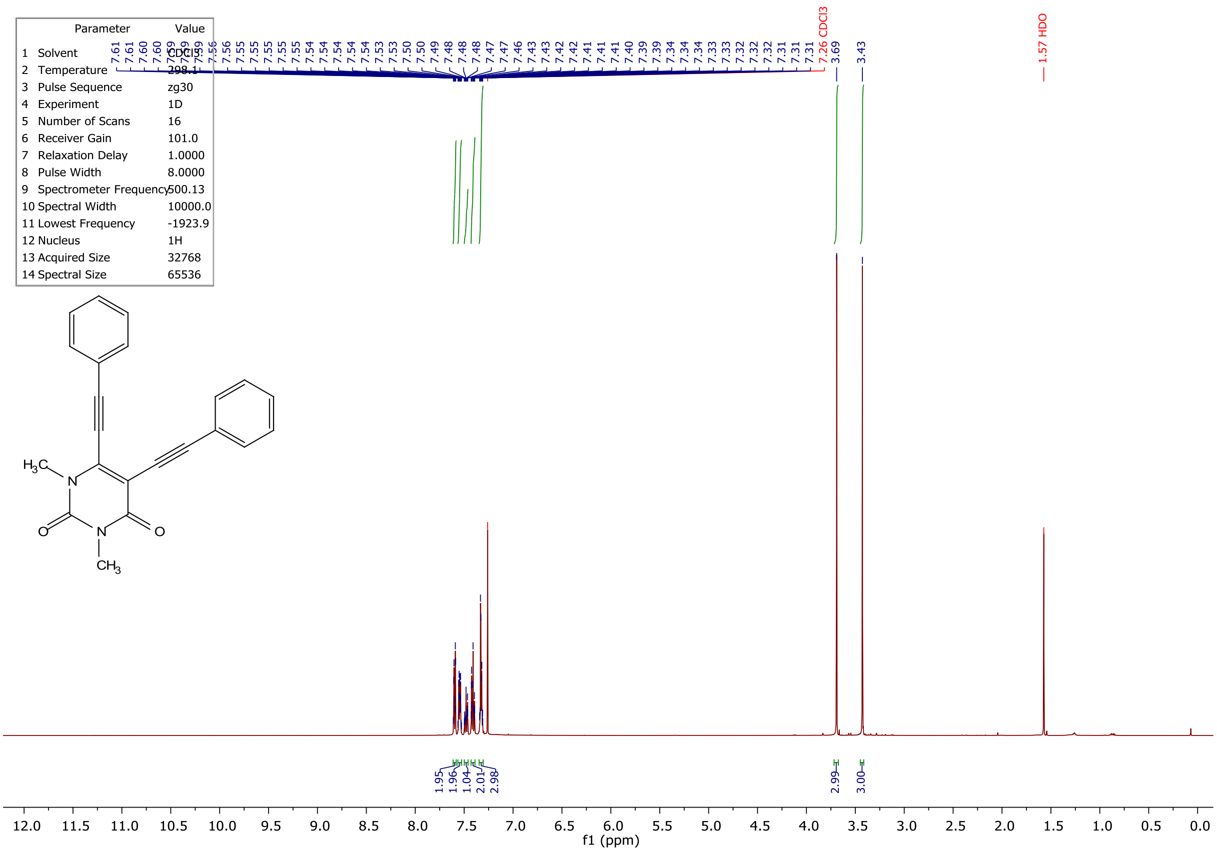

	Parameter	Value
1	Solvent	CDCl3
2	Temperature	298.1
3	Pulse Sequence	zg30
4	Experiment	1D
5	Number of Scans	16
6	Receiver Gain	101.0
7	Relaxation Delay	1.0000
8	Pulse Width	8.0000
9	Spectrometer Frequency	500.13
10	Spectral Width	10000.0
11	Lowest Frequency	-1923.9
12	Nucleus	1H
13	Acquired Size	32768
14	Spectral Size	65536

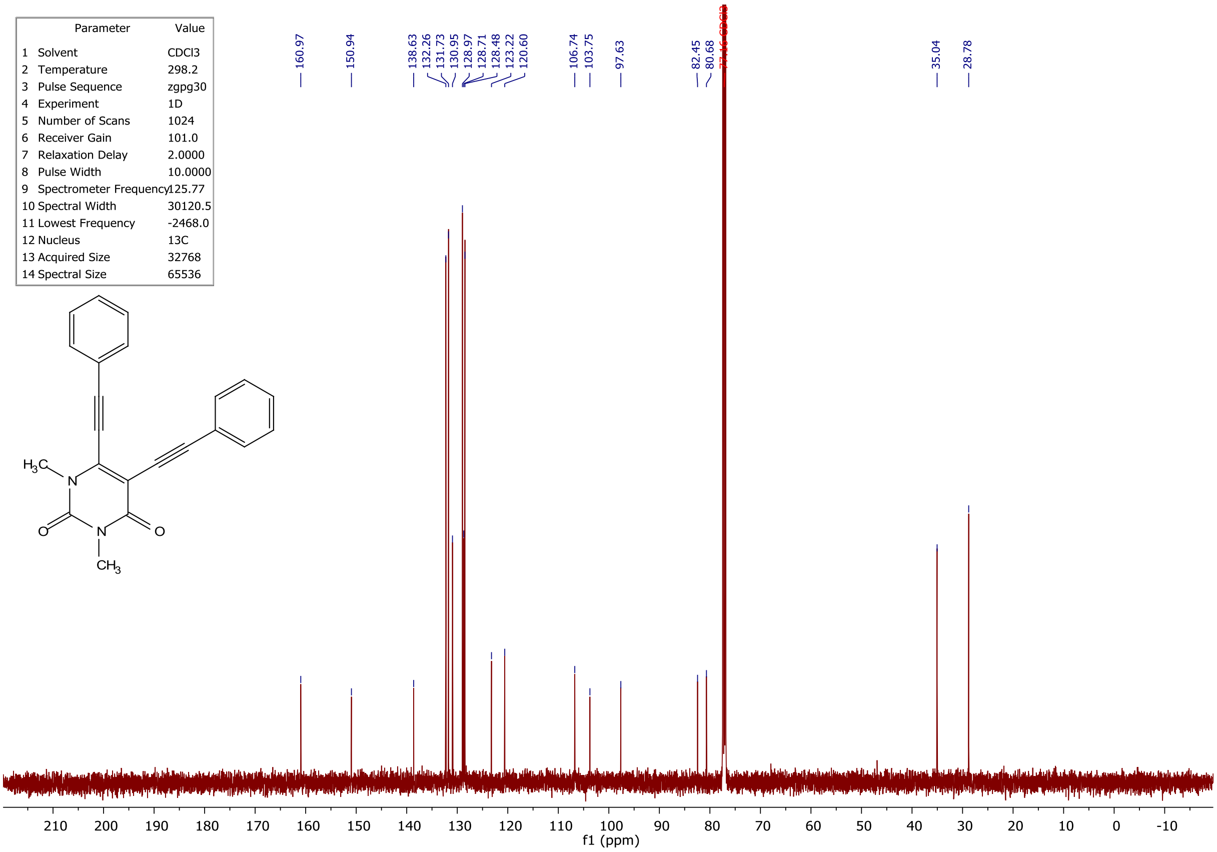

	Parameter	Value
1	Solvent	CDCl3
2	Temperature	298.2
3	Pulse Sequence	zgpg30
4	Experiment	1D
5	Number of Scans	1024
6	Receiver Gain	101.0
7	Relaxation Delay	2.0000
8	Pulse Width	10.0000
9	Spectrometer Frequency	125.77
10	Spectral Width	30120.5
11	Lowest Frequency	-2468.0
12	Nucleus	13C
13	Acquired Size	32768
14	Spectral Size	65536

1,3-Dimethyl-5,6-[2-(4-methoxyphenyl)ethynyl]uracil (4d)

	Parameter	Value
1	Solvent	CDCl3
2	Temperature	298.2
3	Pulse Sequence	zg30
4	Experiment	1D
5	Number of Scans	16
6	Receiver Gain	101.0
7	Relaxation Delay	1.0000
8	Pulse Width	8.0000
9	Spectrometer Frequency	500.13
10	Spectral Width	10000.0
11	Lowest Frequency	-1923.8
12	Nucleus	1H
13	Acquired Size	32768
14	Spectral Size	65536

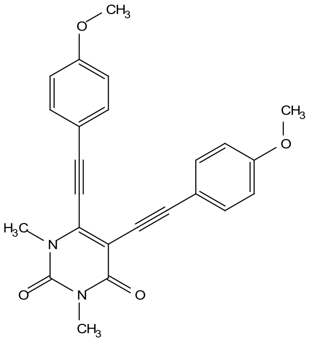

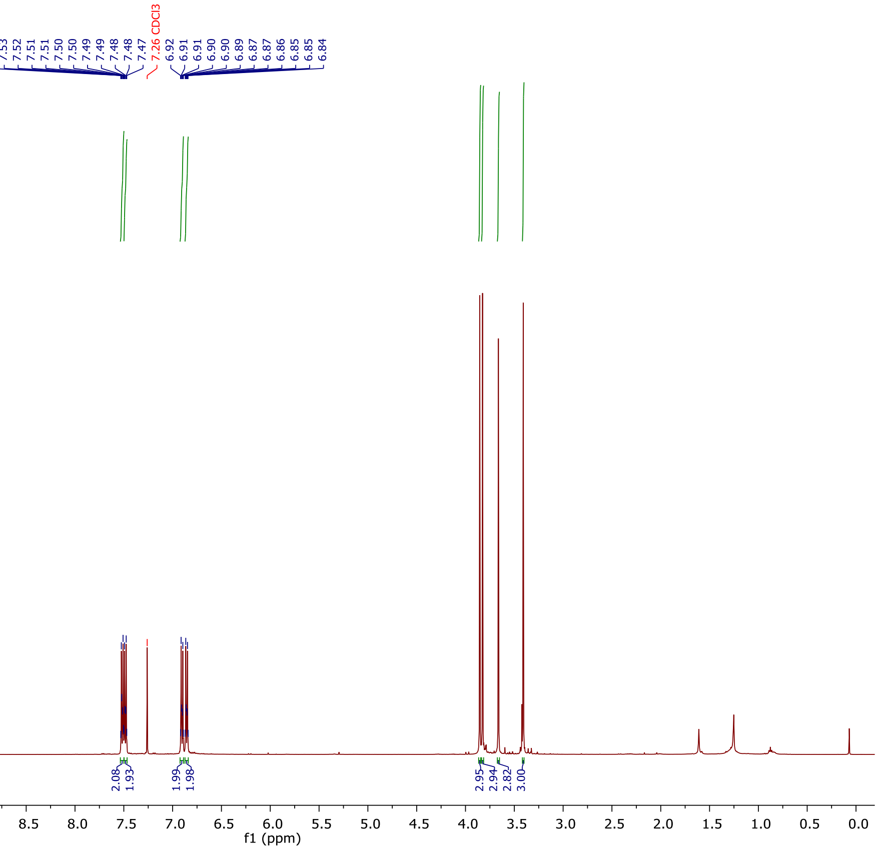

1,3-Dimethyl-5,6-[2-(4-dimethylaminophenyl)ethynyl]uracil (4e)

Parameter	Value
1 Solvent	CDCl3
2 Temperature	298.2
3 Pulse Sequence	zg30
4 Experiment	1D
5 Number of Scans	16
6 Receiver Gain	362.0
7 Relaxation Delay	1.0000
8 Pulse Width	10.0000
9 Spectrometer Frequency	250.13
10 Spectral Width	5165.3
11 Lowest Frequency	-1038.6
12 Nucleus	1H
13 Acquired Size	32768
14 Spectral Size	65536

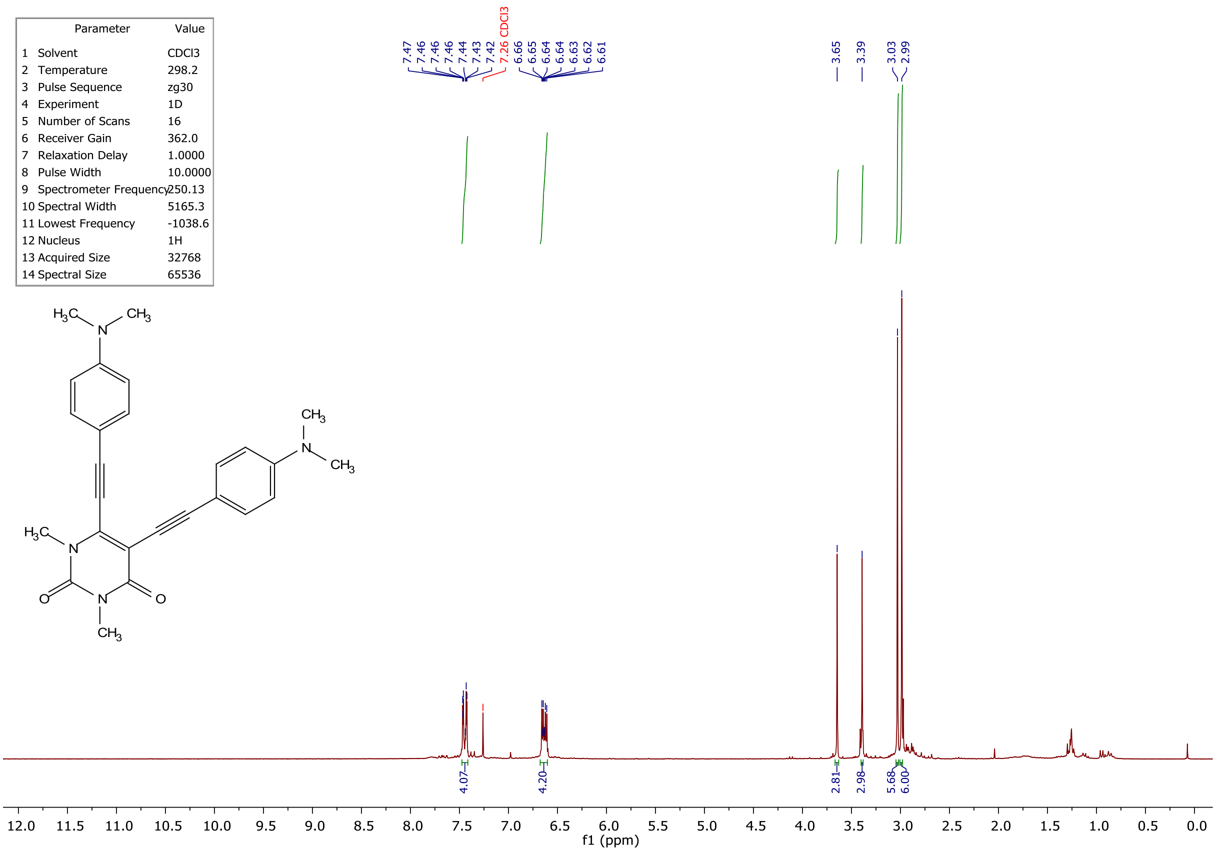

Parameter	Value
1 Solvent	CDCl3
2 Temperature	298.1
3 Pulse Sequence	zgpg30
4 Experiment	1D
5 Number of Scans	1024
6 Receiver Gain	2050.0
7 Relaxation Delay	2.0000
8 Pulse Width	10.0000
9 Spectrometer Frequency	62.90
10 Spectral Width	15000.0
11 Lowest Frequency	-1202.8
12 Nucleus	13C
13 Acquired Size	32768
14 Spectral Size	65536

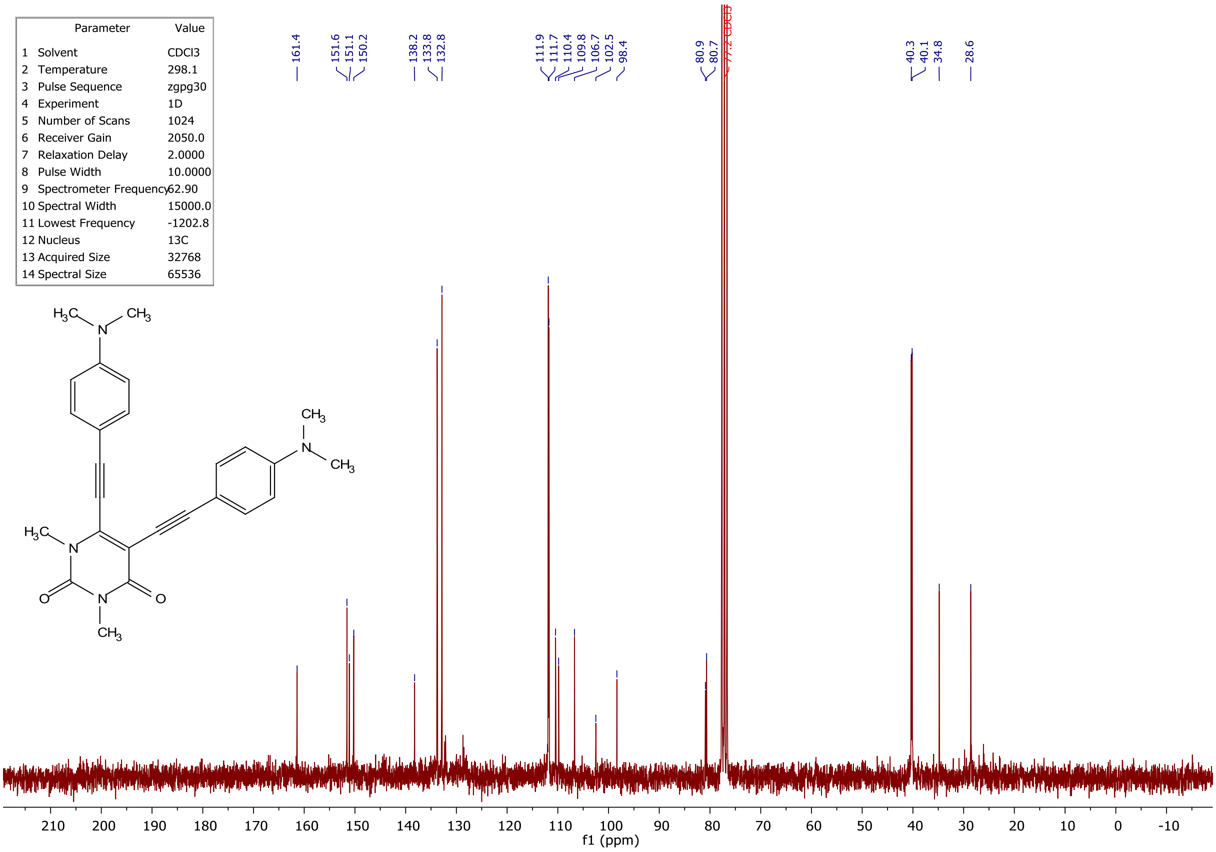

1,3-Dimethyl-5-[2-(4-fluorophenyl)ethynyl]-6-[2-(4-methylphenyl)-ethynyl]uracil (4h)

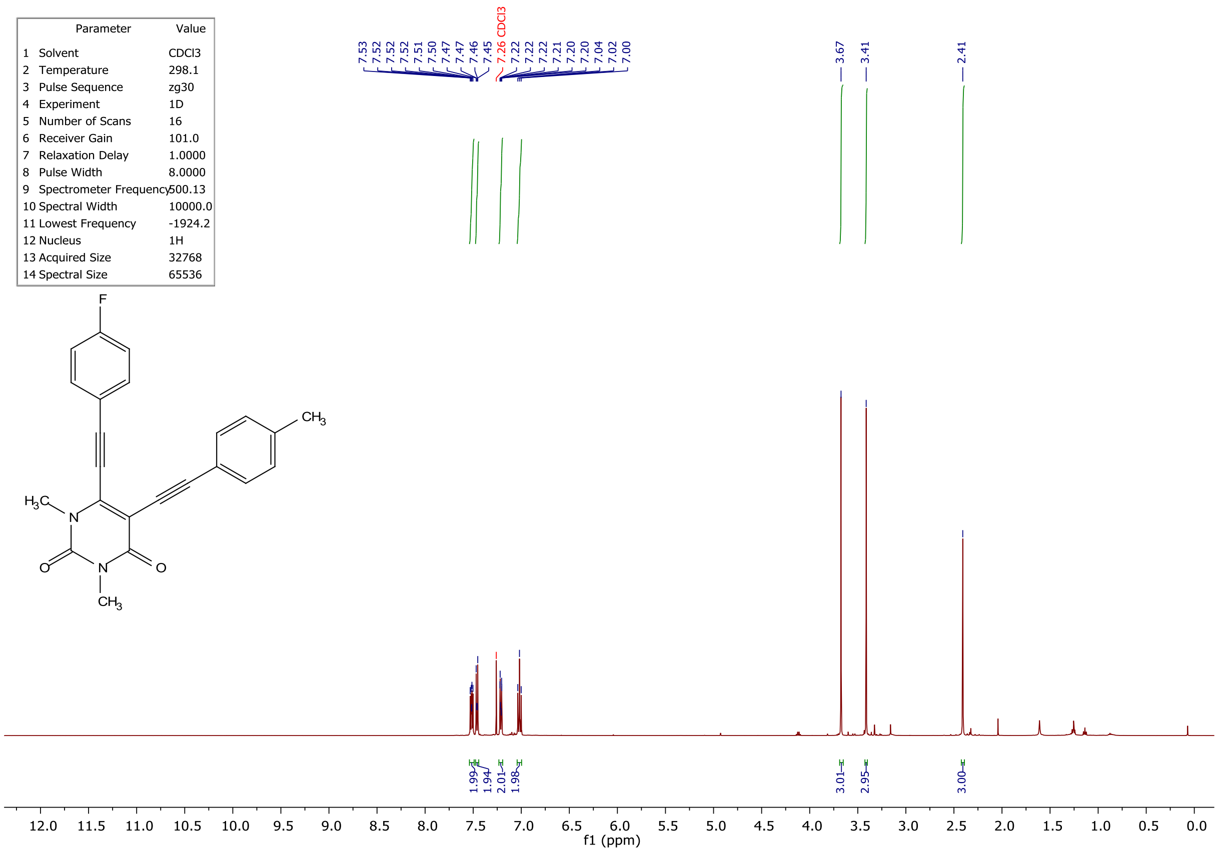

	Parameter	Value
1	Solvent	CDCl3
2	Temperature	298.1
3	Pulse Sequence	zg30
4	Experiment	1D
5	Number of Scans	16
6	Receiver Gain	101.0
7	Relaxation Delay	1.0000
8	Pulse Width	8.0000
9	Spectrometer Frequency	500.13
10	Spectral Width	10000.0
11	Lowest Frequency	-1924.2
12	Nucleus	1H
13	Acquired Size	32768
14	Spectral Size	65536

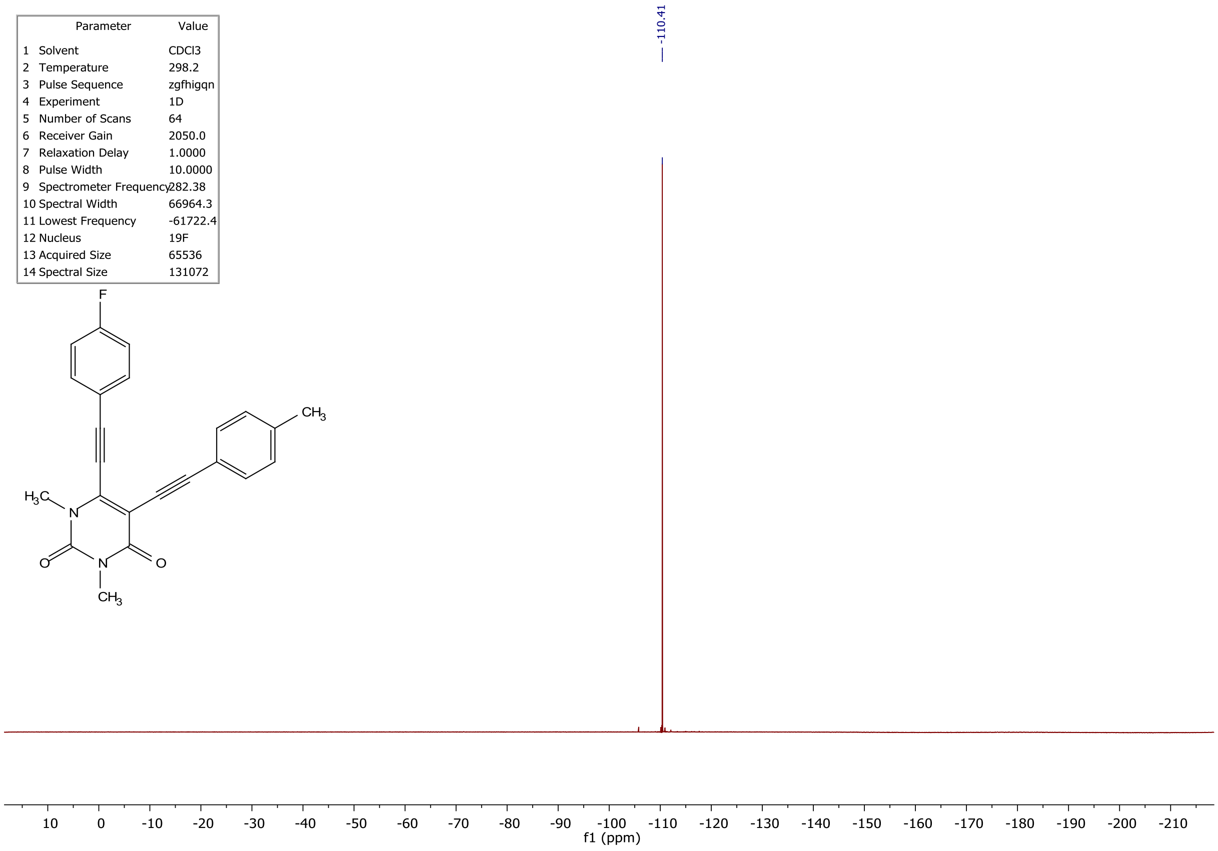

	Parameter	Value
1	Solvent	CDCl3
2	Temperature	298.2
3	Pulse Sequence	zgfhigqn
4	Experiment	1D
5	Number of Scans	64
6	Receiver Gain	2050.0
7	Relaxation Delay	1.0000
8	Pulse Width	10.0000
9	Spectrometer Frequency	282.38
10	Spectral Width	66964.3
11	Lowest Frequency	-61722.4
12	Nucleus	19F
13	Acquired Size	65536
14	Spectral Size	131072

	Parameter	Value
1	Solvent	CDCl3
2	Temperature	298.2
3	Pulse Sequence	zgpg30
4	Experiment	1D
5	Number of Scans	1024
6	Receiver Gain	101.0
7	Relaxation Delay	2.0000
8	Pulse Width	10.0000
9	Spectrometer Frequency	125.77
10	Spectral Width	30120.5
11	Lowest Frequency	-2468.7
12	Nucleus	13C
13	Acquired Size	32768
14	Spectral Size	65536

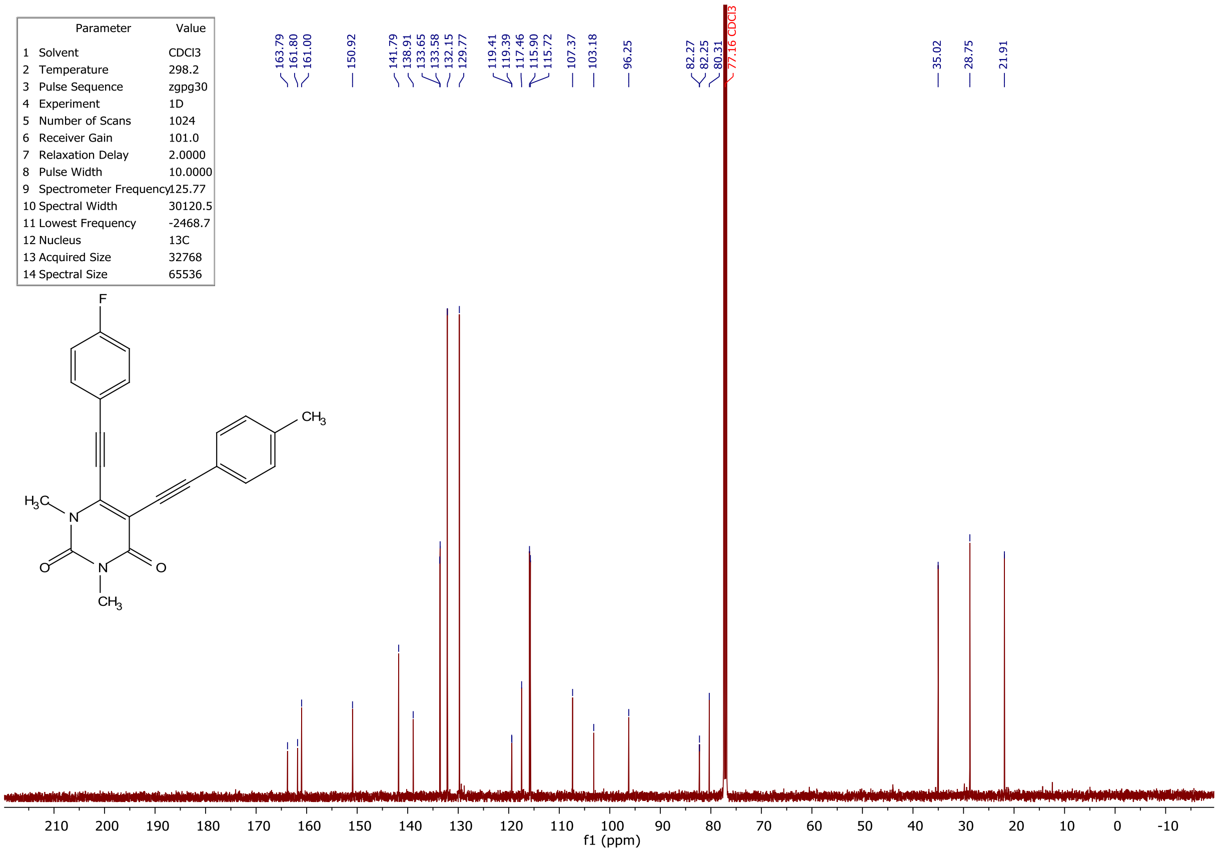

1,3-Dimethyl-5-(4-methylphenyl)-6-[2-(4-methylphenyl)ethynyl]uracil (5a)

	Parameter	Value
1	Solvent	CDCl3
2	Temperature	298.2
3	Pulse Sequence	zg30
4	Experiment	1D
5	Number of Scans	16
6	Receiver Gain	161.0
7	Relaxation Delay	2.0000
8	Pulse Width	10.0000
9	Spectrometer Frequency	300.13
10	Spectral Width	6188.1
11	Lowest Frequency	-1246.6
12	Nucleus	1H
13	Acquired Size	32768
14	Spectral Size	65536

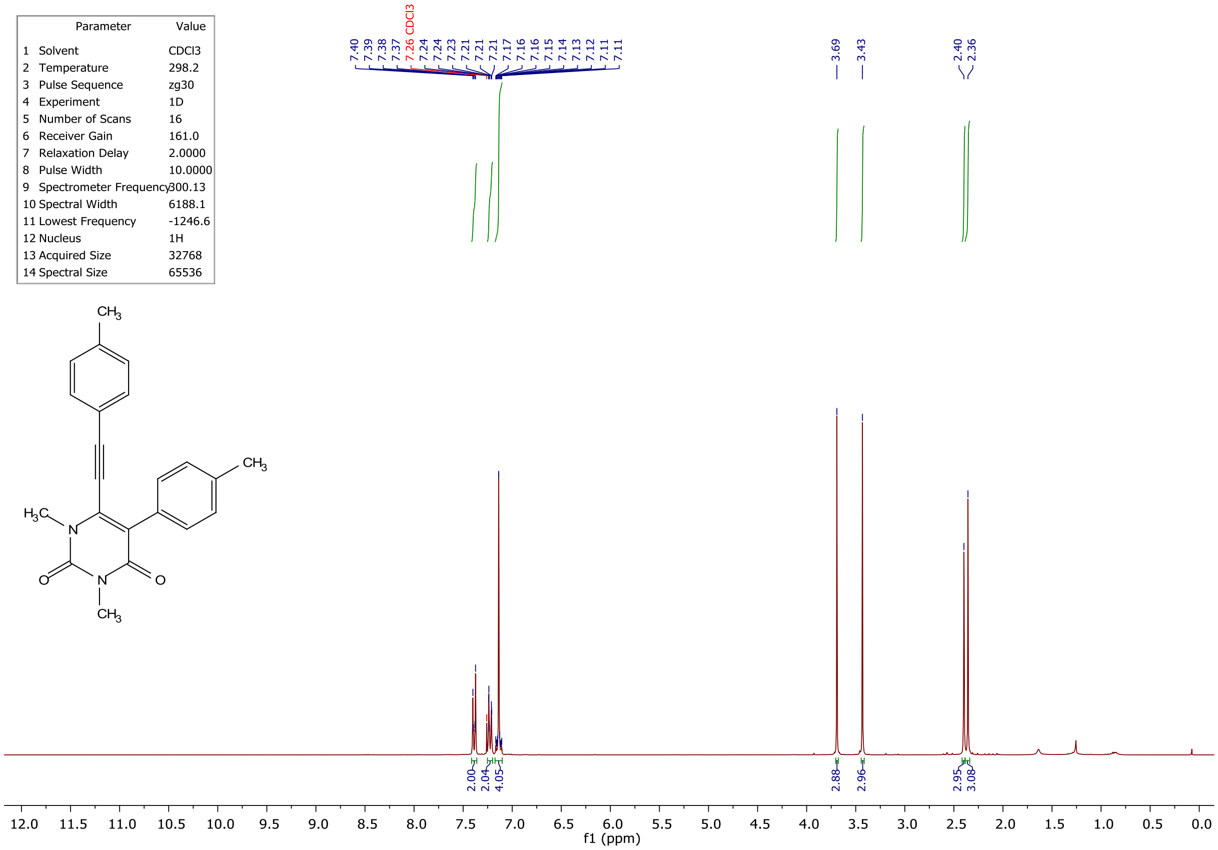

	Parameter	Value
1	Solvent	CDCl3
2	Temperature	298.8
3	Pulse Sequence	zgpg30
4	Experiment	1D
5	Number of Scans	1024
6	Receiver Gain	2050.0
7	Relaxation Delay	2.0000
8	Pulse Width	10.0000
9	Spectrometer Frequency	75.48
10	Spectral Width	18028.8
11	Lowest Frequency	-1459.2
12	Nucleus	13C
13	Acquired Size	32768
14	Spectral Size	65536

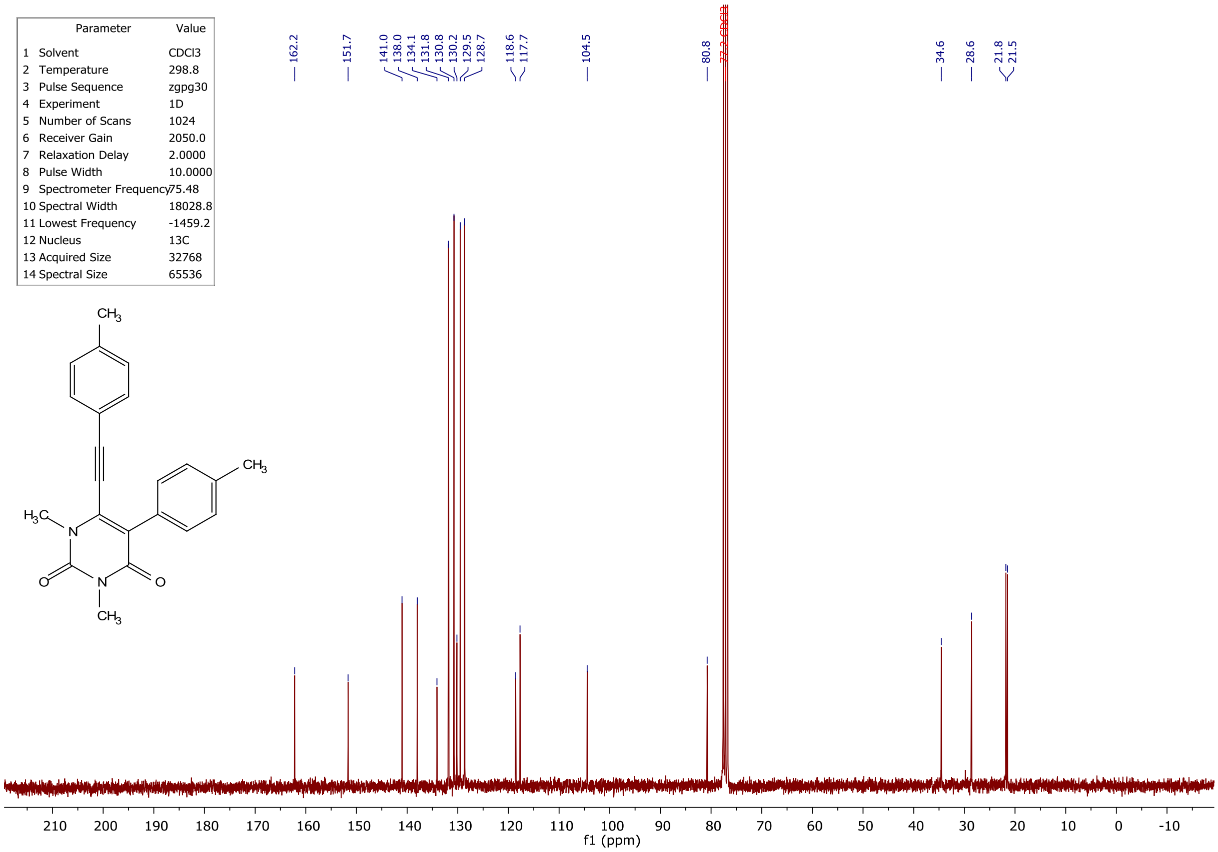

1,3-Dimethyl-5-(4-dimethylaminophenyl)-6-[2-phenylethynyl]uracil (5b)

	Parameter	Value
1	Solvent	CDCl3
2	Temperature	298.2
3	Pulse Sequence	zg30
4	Experiment	1D
5	Number of Scans	16
6	Receiver Gain	101.0
7	Relaxation Delay	1.0000
8	Pulse Width	8.0000
9	Spectrometer Frequency	500.13
10	Spectral Width	10000.0
11	Lowest Frequency	-1923.6
12	Nucleus	1H
13	Acquired Size	32768
14	Spectral Size	65536

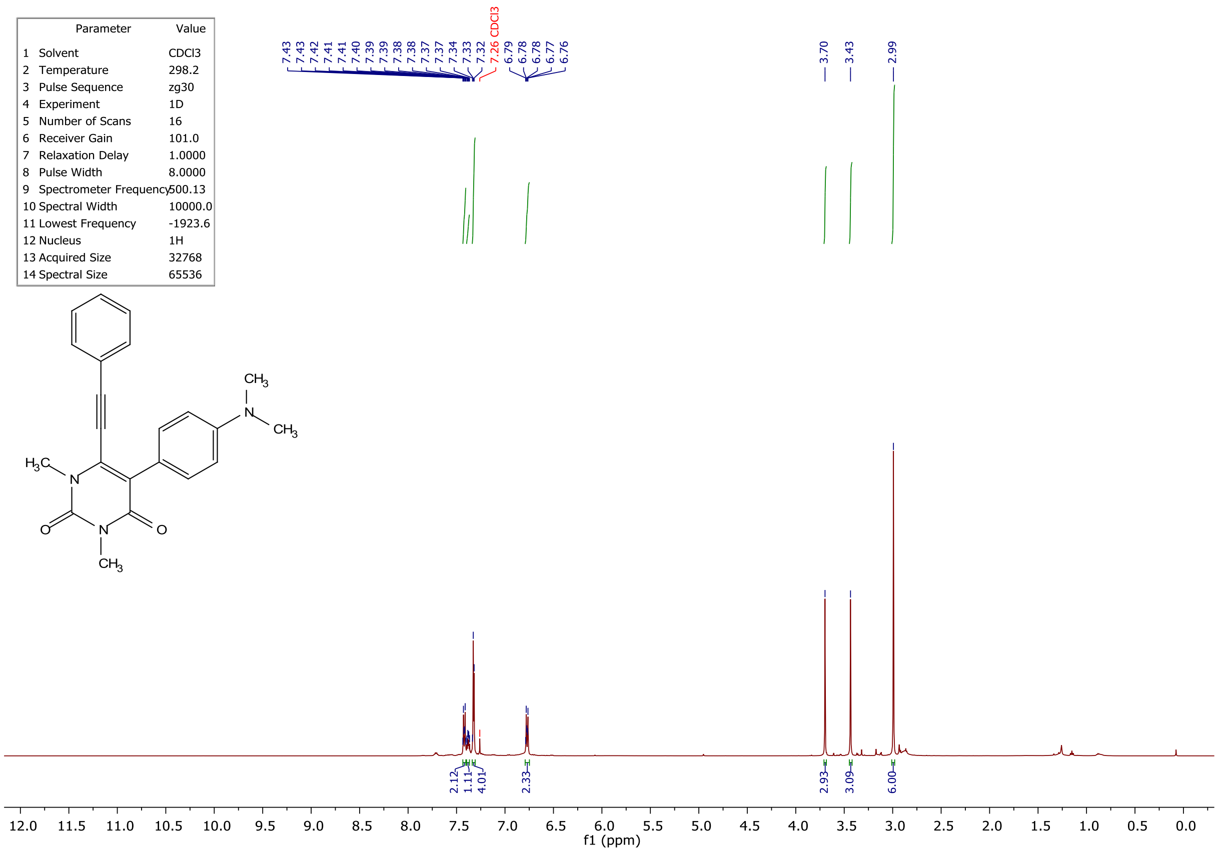

	Parameter	Value
1	Solvent	CDCl3
2	Temperature	298.1
3	Pulse Sequence	zgpg30
4	Experiment	1D
5	Number of Scans	1024
6	Receiver Gain	101.0
7	Relaxation Delay	2.0000
8	Pulse Width	10.0000
9	Spectrometer Frequency	125.77
10	Spectral Width	30120.5
11	Lowest Frequency	-2472.9
12	Nucleus	13C
13	Acquired Size	32768
14	Spectral Size	65536

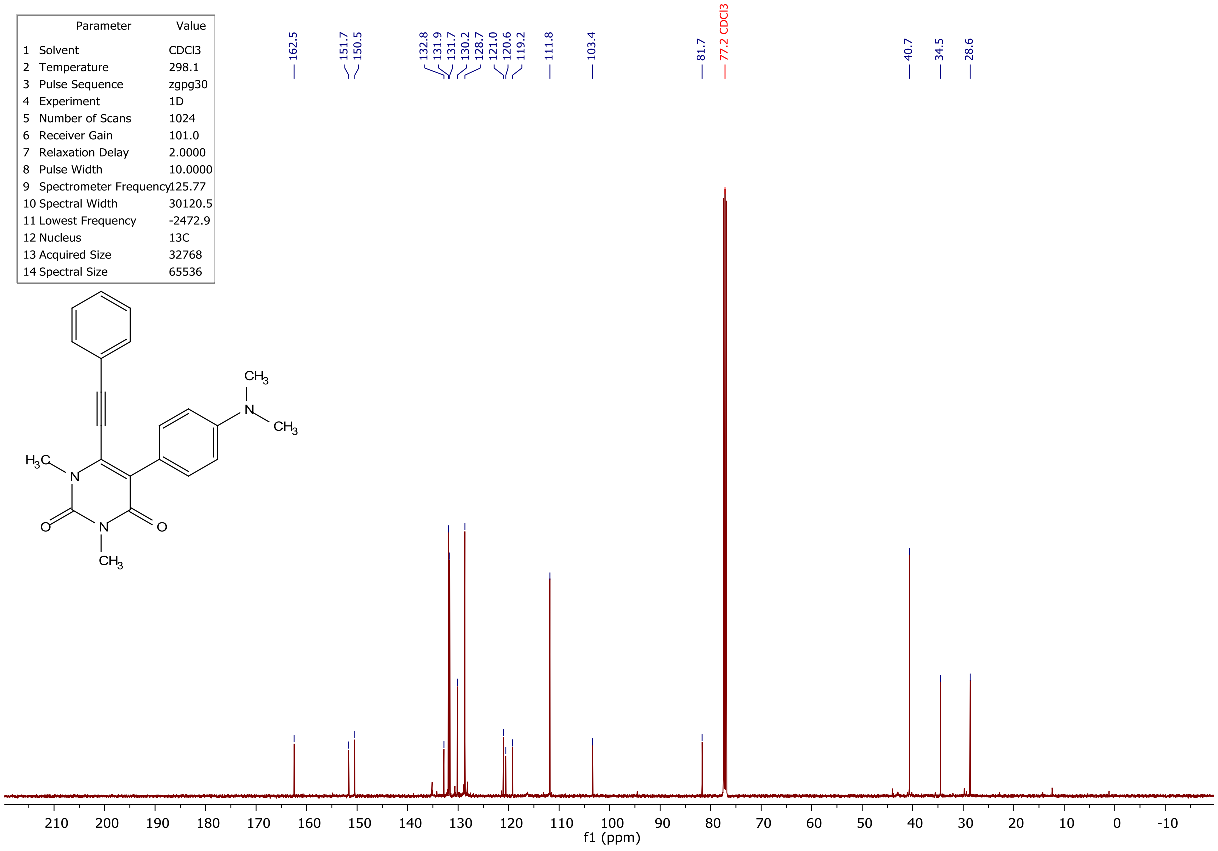

1,3-Dimethyl-5-(4-trifluoromethylphenyl)-6-[2-phenylethynyl]uracil (5c)

	Parameter	Value
1	Solvent	CDCl3
2	Temperature	298.2
3	Pulse Sequence	zg30
4	Experiment	1D
5	Number of Scans	16
6	Receiver Gain	101.0
7	Relaxation Delay	1.0000
8	Pulse Width	8.0000
9	Spectrometer Frequency	500.13
10	Spectral Width	10000.0
11	Lowest Frequency	-1961.2
12	Nucleus	1H
13	Acquired Size	32768
14	Spectral Size	65536

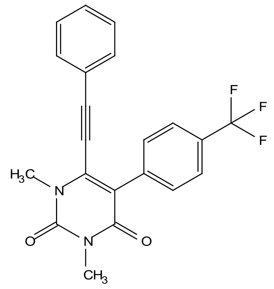

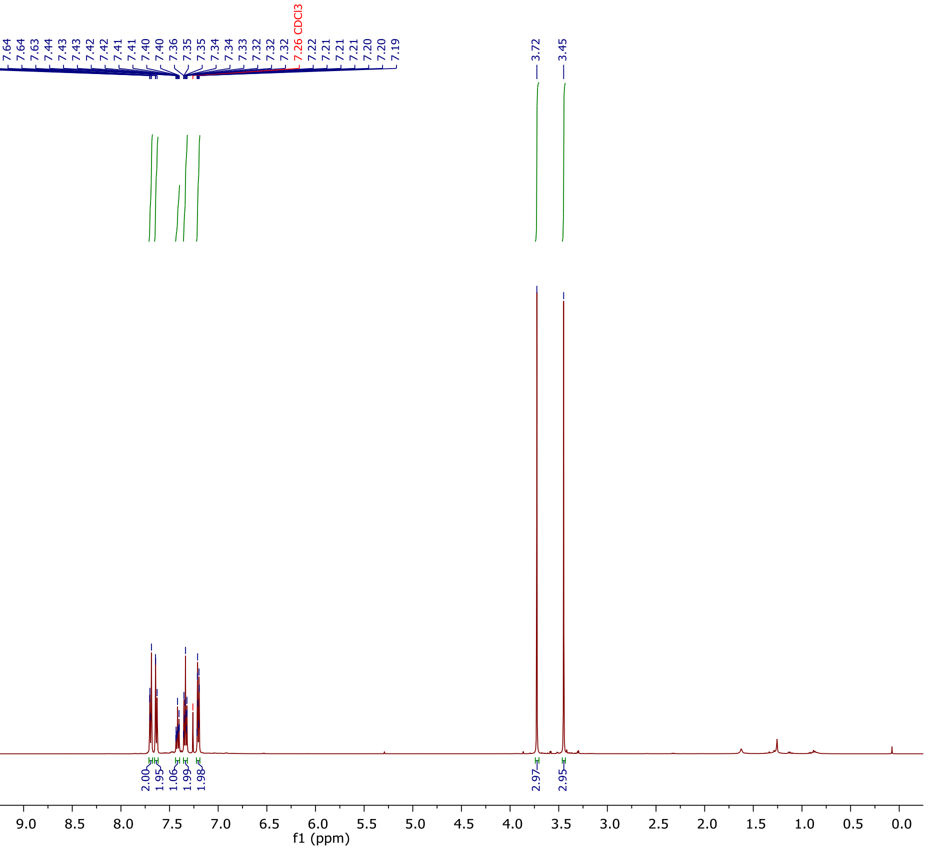

	Parameter	Value
1	Solvent	CDCl3
2	Temperature	298.3
3	Pulse Sequence	zgig
4	Experiment	1D
5	Number of Scans	16
6	Receiver Gain	101.0
7	Relaxation Delay	1.0000
8	Pulse Width	12.0000
9	Spectrometer Frequency	470.55
10	Spectral Width	113636.4
11	Lowest Frequency	-103877.4
12	Nucleus	19F
13	Acquired Size	65536
14	Spectral Size	131072

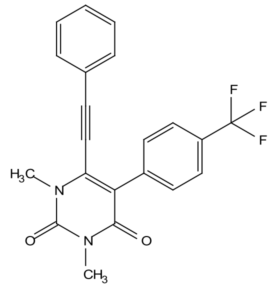

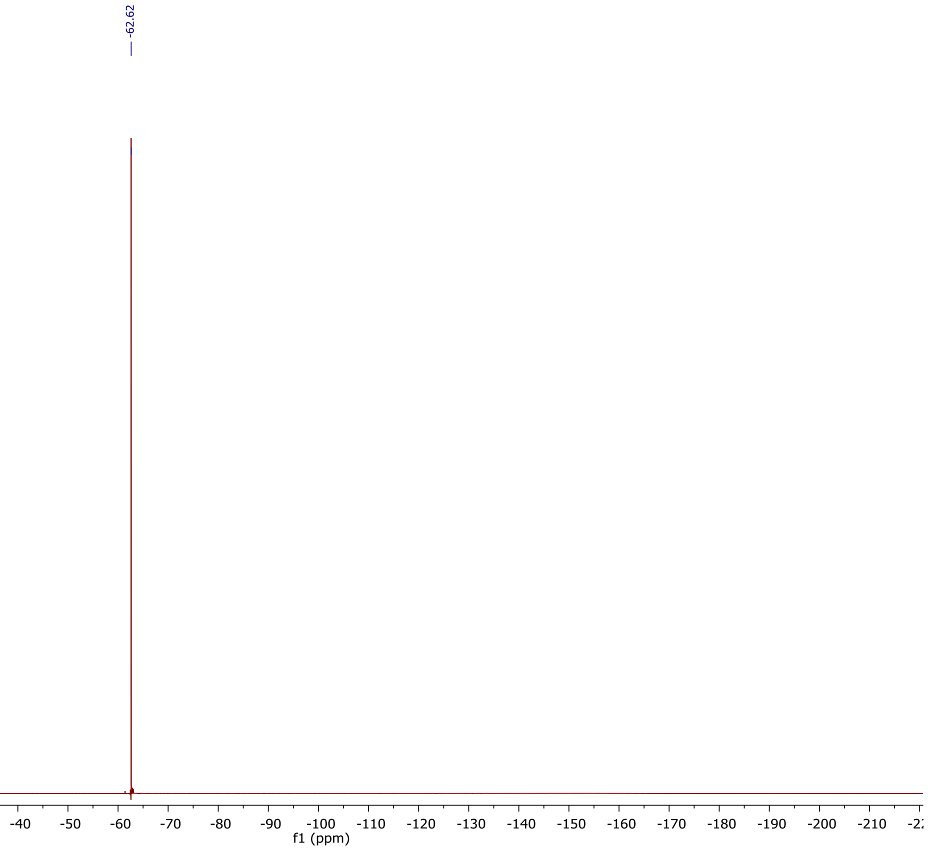

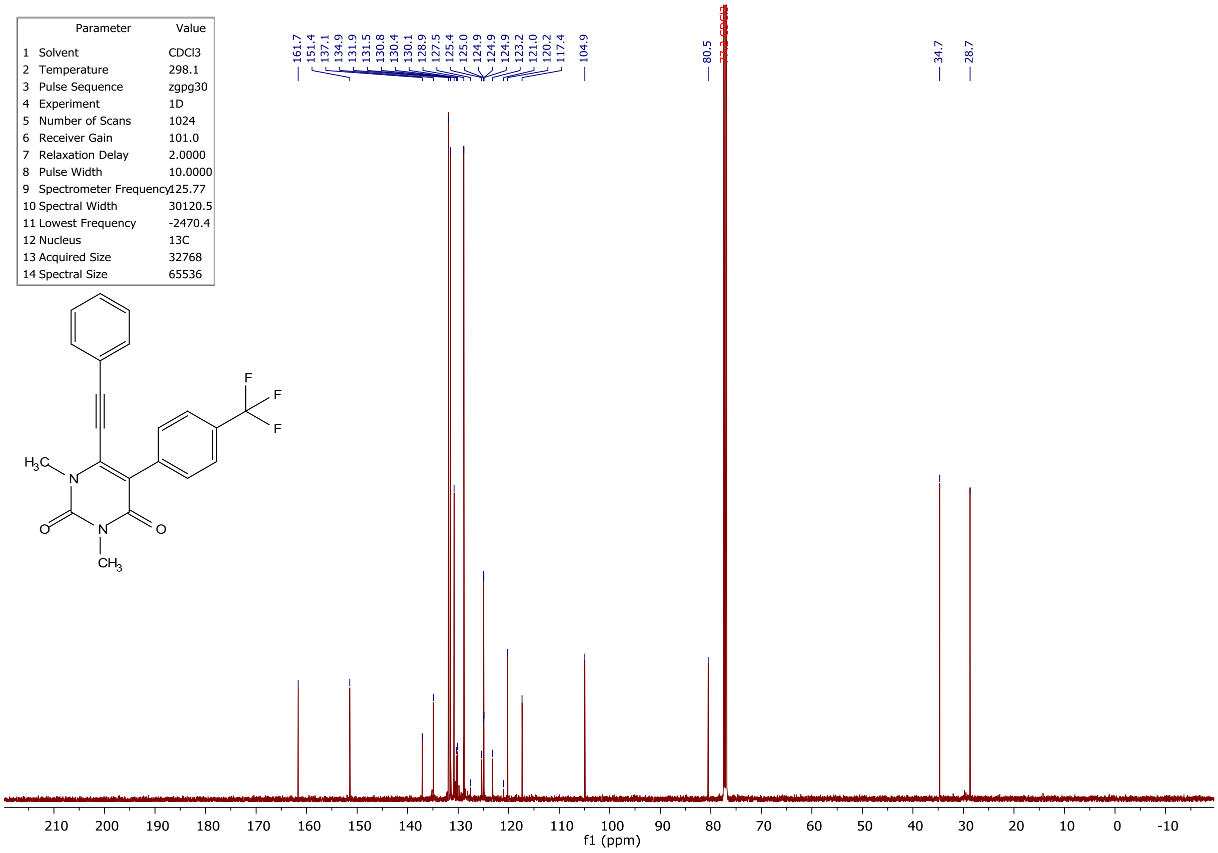

Parameter Value
1 Solvent CDCl3
2 Temperature 298.1
3 Pulse Sequence zgpg30
4 Experiment 1D
5 Number of Scans 1024
6 Receiver Gain 101.0
7 Relaxation Delay 2.0000
8 Pulse Width 10.0000
9 Spectrometer Frequency 125.77
10 Spectral Width 30120.5
11 Lowest Frequency -2470.4
12 Nucleus 13C
13 Acquired Size 32768
14 Spectral Size 65536
161.7
151.4
137.1
134.9
131.9
131.5
130.8
130.4
130.1
128.9
127.5
125.4
125.0
124.9
124.9
124.9
123.2
121.0
120.2
117.4
104.9
80.5
34.7
28.7
F
F
F
H3C
N
N
O
O
CH3
210 200 190 180 170 160 150 140 130 120 110 100 90 80 70 60 50 40 30 20 10 0 -10
f1 (ppm)

1,3-Dimethyl-5-(4-dimethylaminophenyl)-6-[2-(4-methylphenyl)ethynyl]uracil (5d)

	Parameter	Value
1	Solvent	CDCl3
2	Temperature	298.7
3	Pulse Sequence	zg30
4	Experiment	1D
5	Number of Scans	16
6	Receiver Gain	71.8
7	Relaxation Delay	2.0000
8	Pulse Width	10.0000
9	Spectrometer Frequency	300.13
10	Spectral Width	6188.1
11	Lowest Frequency	-1246.3
12	Nucleus	1H
13	Acquired Size	32768
14	Spectral Size	65536

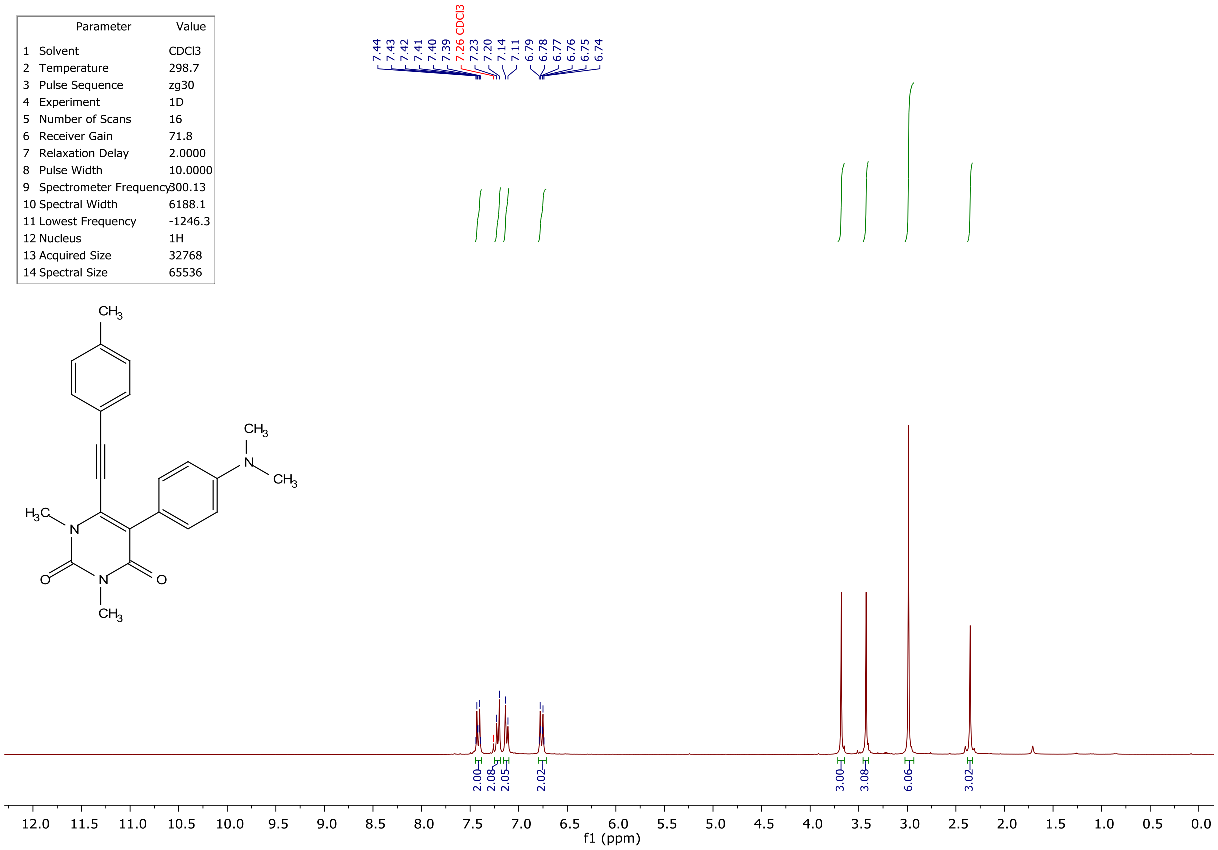

	Parameter	Value
1	Solvent	CDCl3
2	Temperature	299.5
3	Pulse Sequence	zgpg30
4	Experiment	1D
5	Number of Scans	1024
6	Receiver Gain	2050.0
7	Relaxation Delay	2.0000
8	Pulse Width	10.0000
9	Spectrometer Frequency	75.48
10	Spectral Width	18028.8
11	Lowest Frequency	-1461.5
12	Nucleus	13C
13	Acquired Size	32768
14	Spectral Size	65536

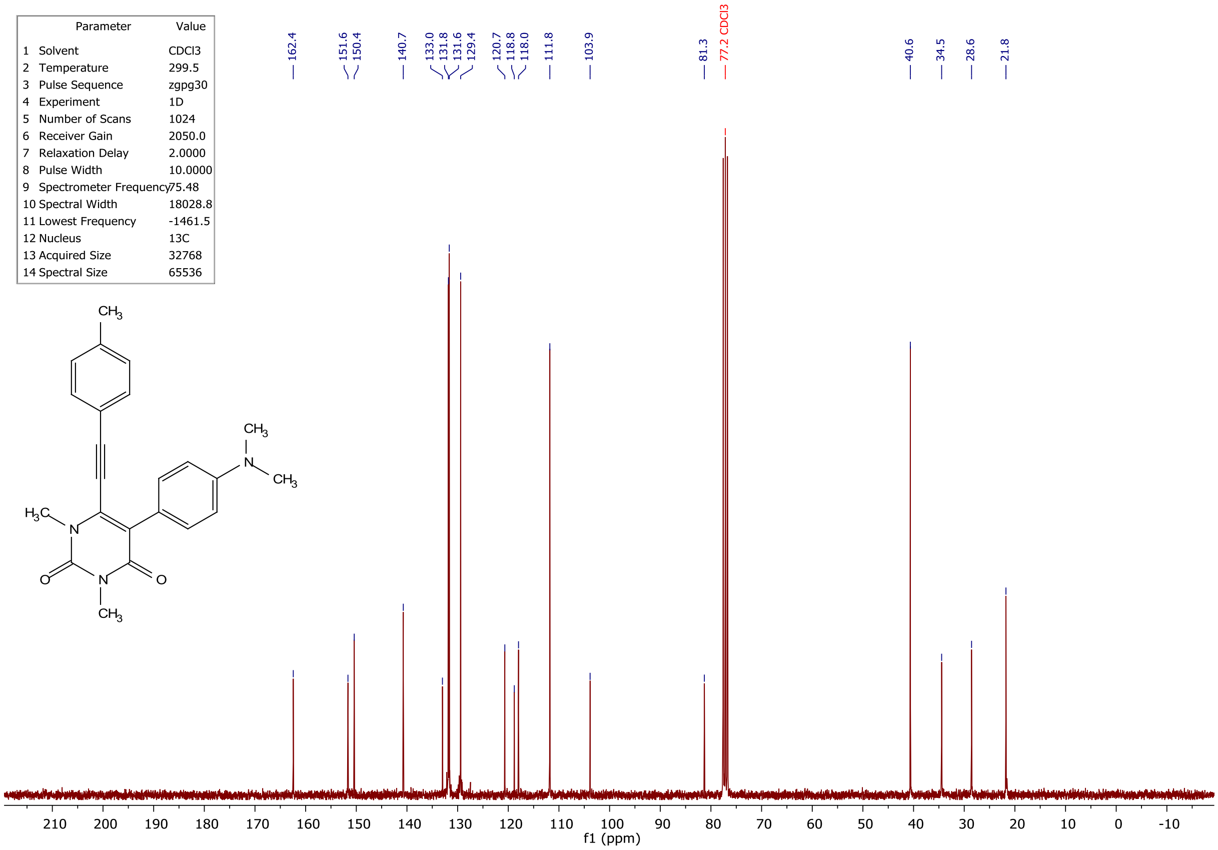

1,3-Dimethyl-5-(4-trifluoromethylphenyl)-6-[2-(4-methylphenyl)ethynyl]uracil (5e)

	Parameter	Value
1	Solvent	CDCl3
2	Temperature	298.2
3	Pulse Sequence	zg30
4	Experiment	1D
5	Number of Scans	16
6	Receiver Gain	228.0
7	Relaxation Delay	2.0000
8	Pulse Width	10.0000
9	Spectrometer Frequency	300.13
10	Spectral Width	6188.1
11	Lowest Frequency	-1247.0
12	Nucleus	1H
13	Acquired Size	32768
14	Spectral Size	65536

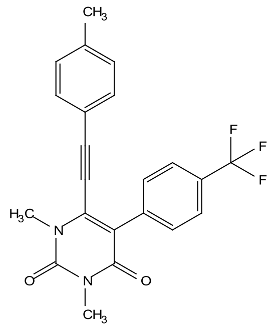

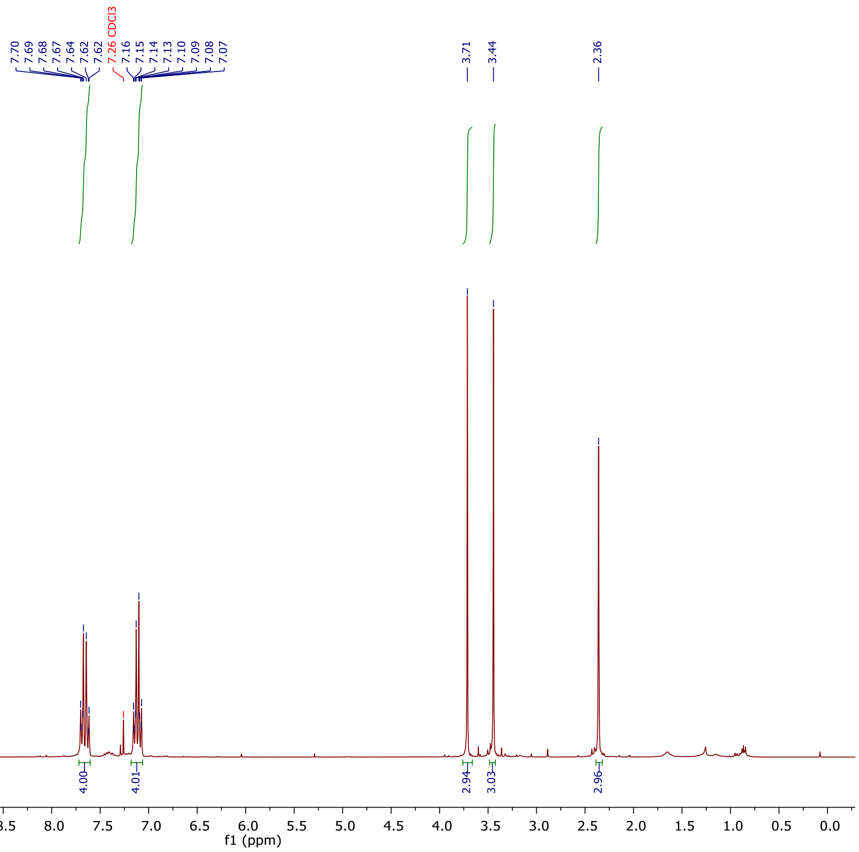

	Parameter	Value
1	Solvent	CDCl3
2	Temperature	298.2
3	Pulse Sequence	zgig
4	Experiment	1D
5	Number of Scans	16
6	Receiver Gain	101.0
7	Relaxation Delay	1.0000
8	Pulse Width	12.0000
9	Spectrometer Frequency	470.55
10	Spectral Width	113636.4
11	Lowest Frequency	-103877.4
12	Nucleus	19F
13	Acquired Size	65536
14	Spectral Size	131072

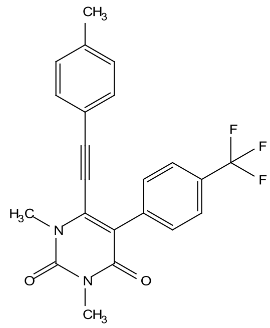

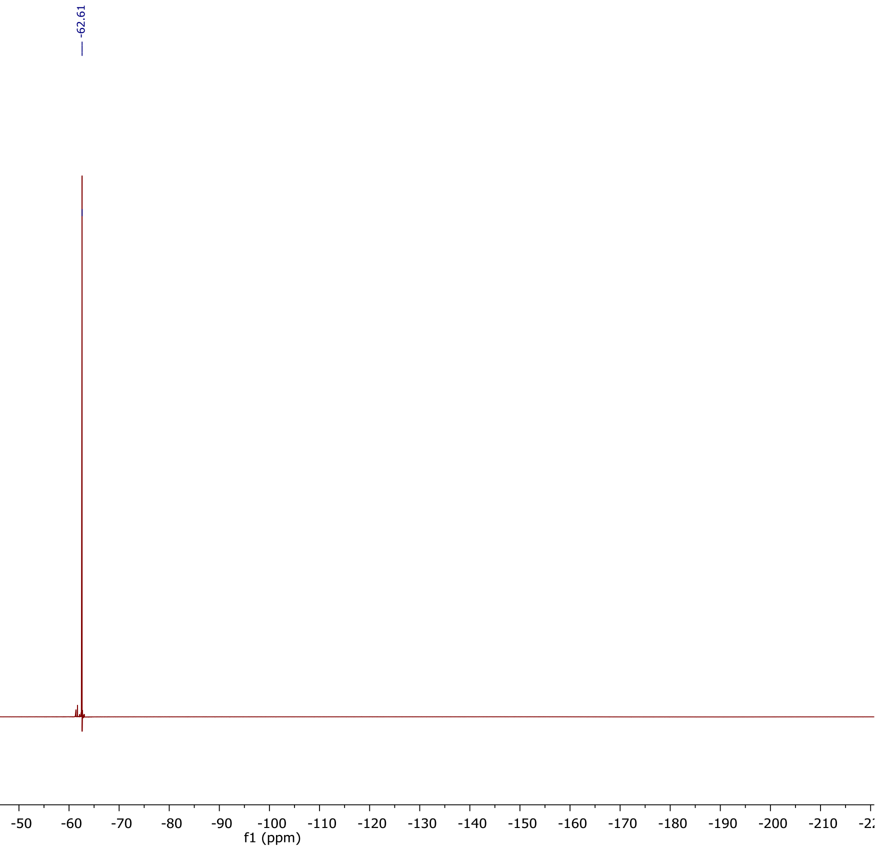

	Parameter	Value
1	Solvent	CDCl3
2	Temperature	298.2
3	Pulse Sequence	zgpg30
4	Experiment	1D
5	Number of Scans	2048
6	Receiver Gain	2050.0
7	Relaxation Delay	2.0000
8	Pulse Width	9.6000
9	Spectrometer Frequency	75.48
10	Spectral Width	18028.8
11	Lowest Frequency	-1459.2
12	Nucleus	13C
13	Acquired Size	32768
14	Spectral Size	65536

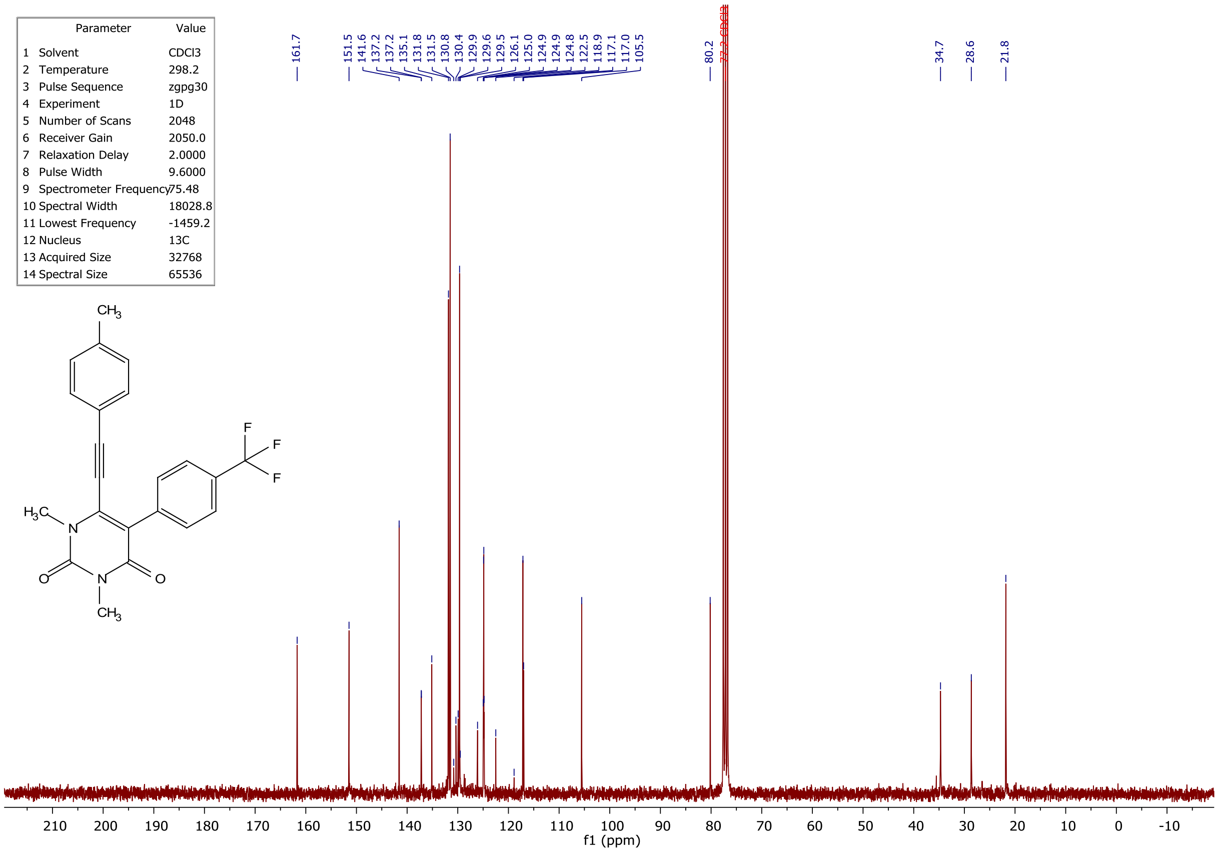

1,3-Dimethyl-5-[2-(4-methylphenyl)ethenyl]-6-[2-(4-methylphenyl)ethynyl]uracil (5f)

Parameter	Value
1 Solvent	CDCl3
2 Temperature	298.2
3 Pulse Sequence	zg30
4 Experiment	1D
5 Number of Scans	16
6 Receiver Gain	80.6
7 Relaxation Delay	2.0000
8 Pulse Width	10.0000
9 Spectrometer Frequency	300.13
10 Spectral Width	6188.1
11 Lowest Frequency	-1257.2
12 Nucleus	1H
13 Acquired Size	32768
14 Spectral Size	65536

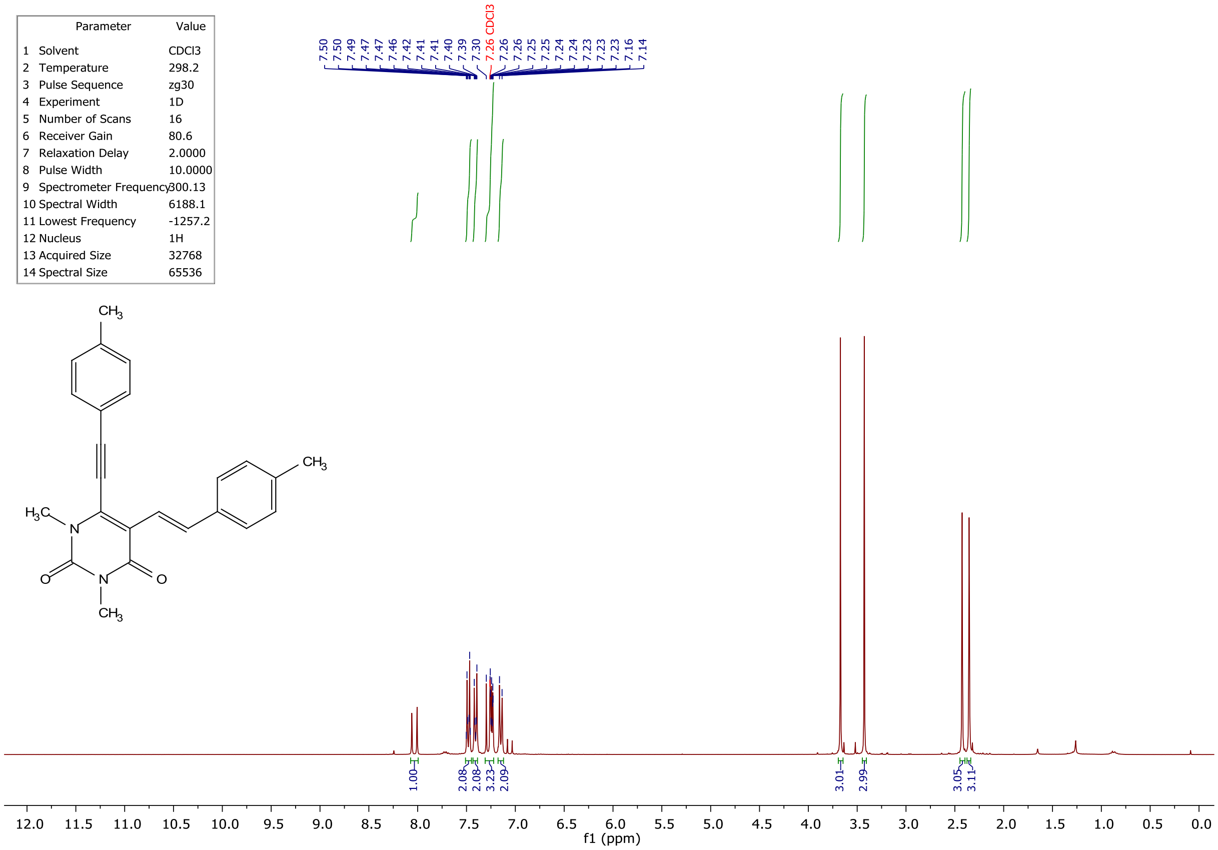

Parameter	Value
1 Solvent	CDCl3
2 Temperature	298.2
3 Pulse Sequence	zgpg30
4 Experiment	1D
5 Number of Scans	1024
6 Receiver Gain	2050.0
7 Relaxation Delay	2.0000
8 Pulse Width	10.0000
9 Spectrometer Frequency	75.48
10 Spectral Width	18028.8
11 Lowest Frequency	-1460.3
12 Nucleus	13C
13 Acquired Size	32768
14 Spectral Size	65536

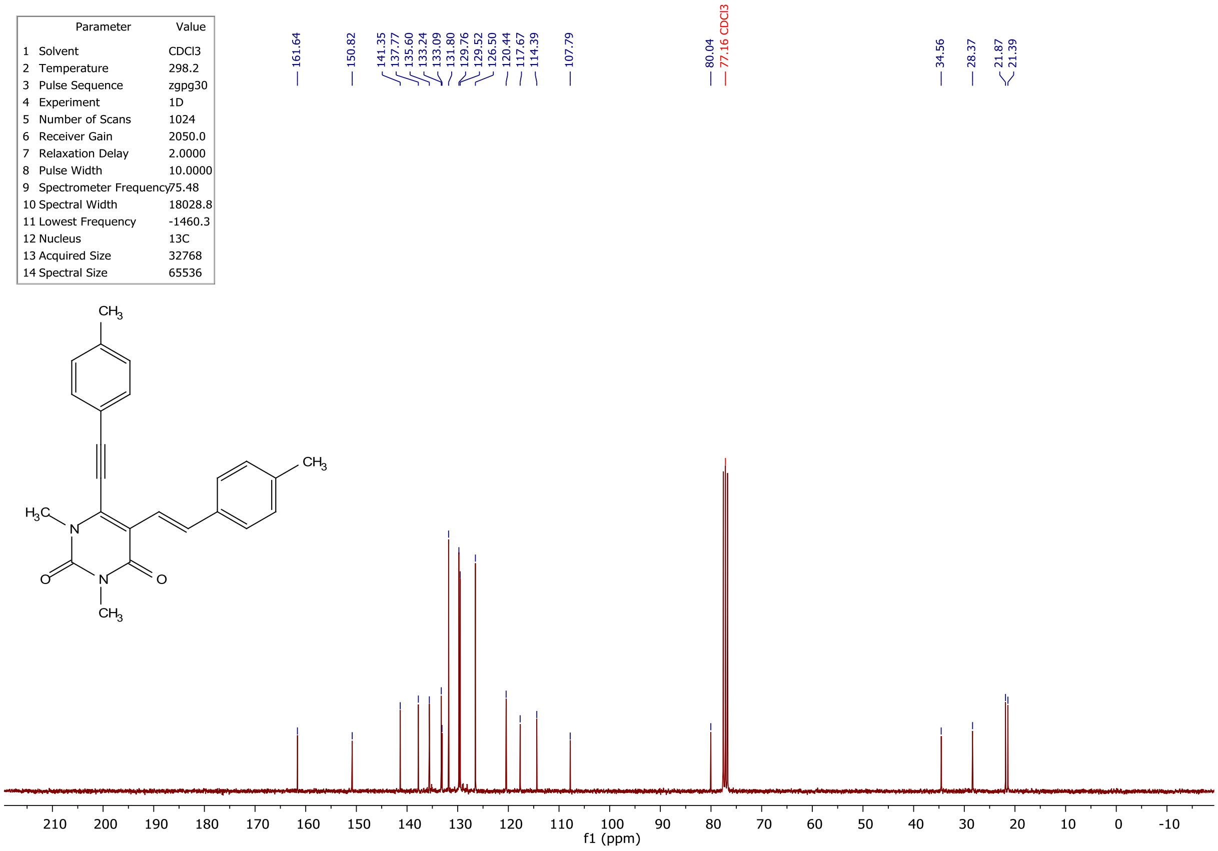

(*E*)-1,3-Dimethyl-6-(*p*-tolylethynyl)-5-(4-(trifluoromethyl)styryl)pyrimidine-2,4(1*H*,3*H*)-dione (5g)

	Parameter	Value
1	Solvent	CDCl3
2	Temperature	298.2
3	Pulse Sequence	zg30
4	Experiment	1D
5	Number of Scans	16
6	Receiver Gain	161.0
7	Relaxation Delay	2.0000
8	Pulse Width	10.0000
9	Spectrometer Frequency	300.13
10	Spectral Width	6188.1
11	Lowest Frequency	-1246.3
12	Nucleus	1H
13	Acquired Size	32768
14	Spectral Size	65536

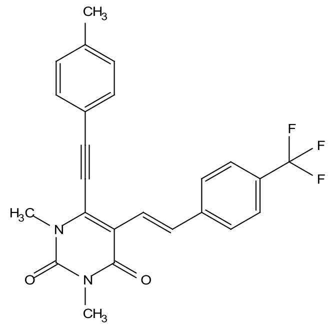

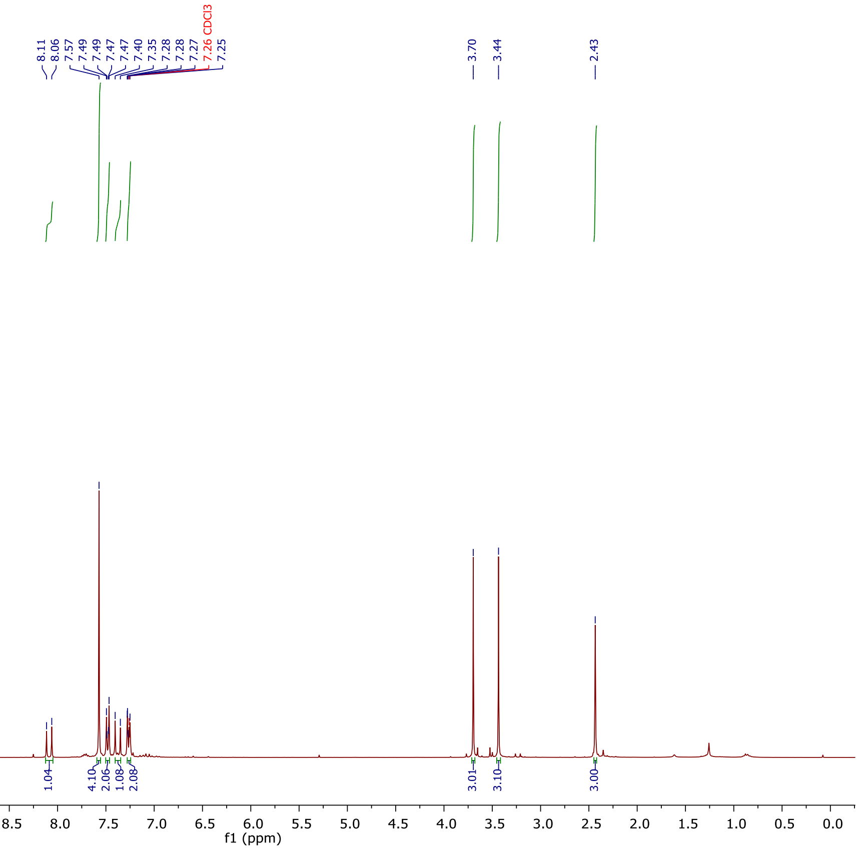

	Parameter	Value
1	Solvent	CDCl3
2	Temperature	298.2
3	Pulse Sequence	zgfhigqn
4	Experiment	1D
5	Number of Scans	64
6	Receiver Gain	2050.0
7	Relaxation Delay	1.0000
8	Pulse Width	10.0000
9	Spectrometer Frequency	282.38
10	Spectral Width	66964.3
11	Lowest Frequency	-61722.4
12	Nucleus	19F
13	Acquired Size	65536
14	Spectral Size	131072

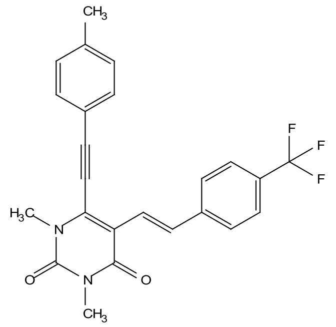

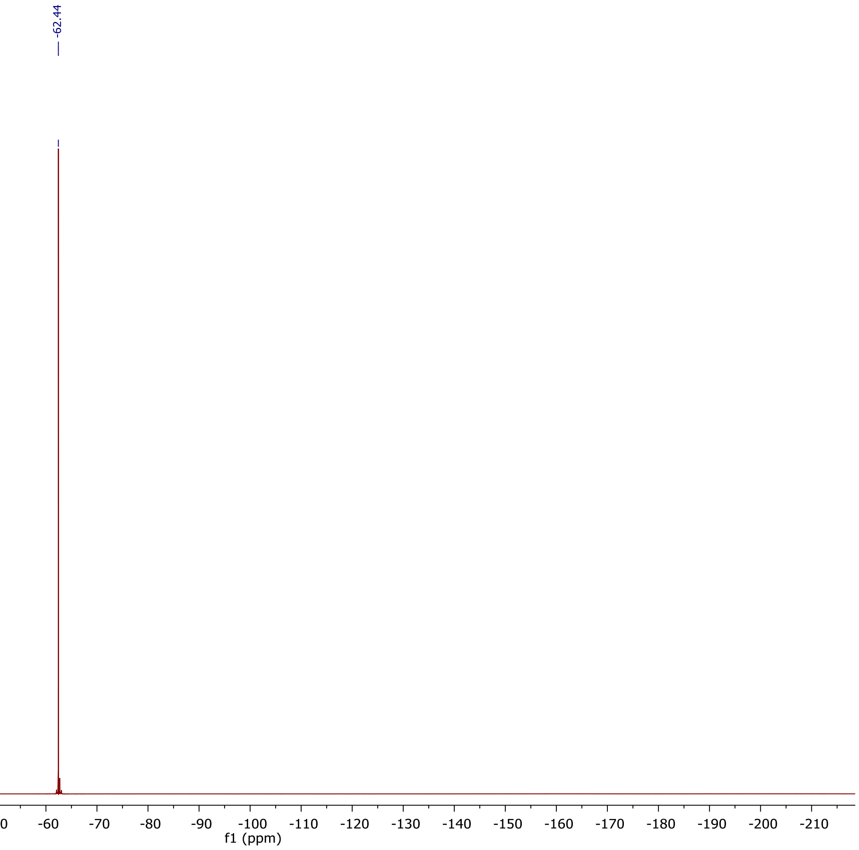

	Parameter	Value
1	Solvent	CDCl3
2	Temperature	298.2
3	Pulse Sequence	zgpg30
4	Experiment	1D
5	Number of Scans	2048
6	Receiver Gain	2050.0
7	Relaxation Delay	2.0000
8	Pulse Width	10.0000
9	Spectrometer Frequency	75.48
10	Spectral Width	18028.8
11	Lowest Frequency	-1458.4
12	Nucleus	13C
13	Acquired Size	32768
14	Spectral Size	65536

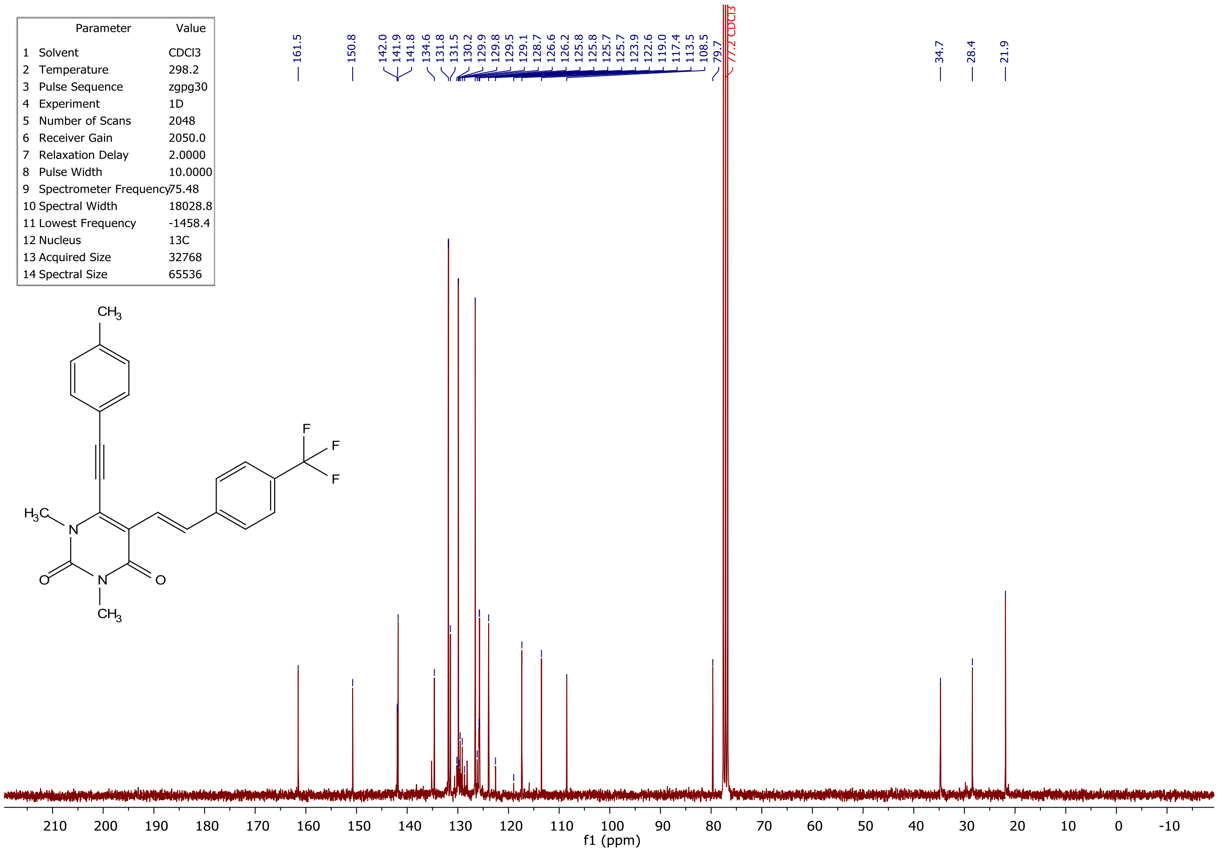

1,3-Dimethyl-5-(4-dimethylaminophenyl)-6-[2-(biphenyl)ethynyl]uracil (5i)

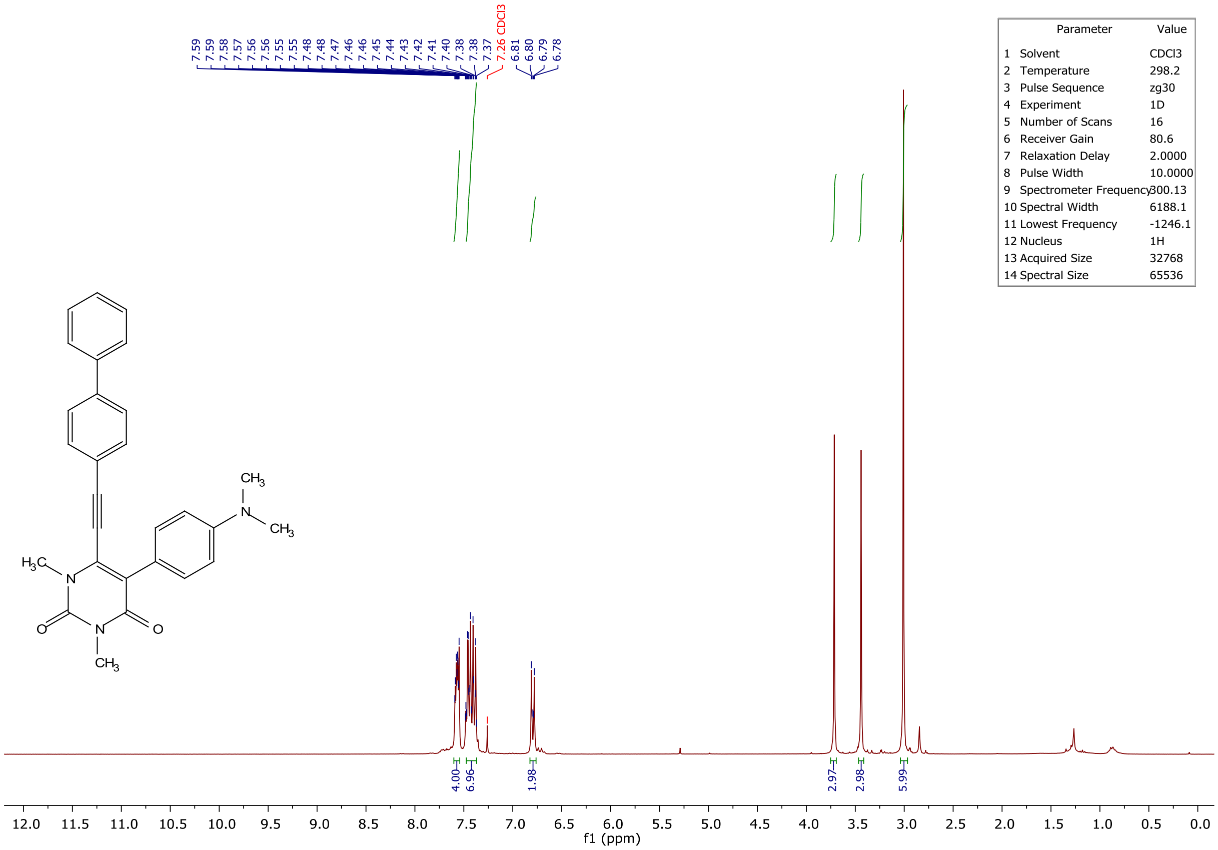

	Parameter	Value
1	Solvent	CDCl3
2	Temperature	298.2
3	Pulse Sequence	zg30
4	Experiment	1D
5	Number of Scans	16
6	Receiver Gain	80.6
7	Relaxation Delay	2.0000
8	Pulse Width	10.0000
9	Spectrometer Frequency	300.13
10	Spectral Width	6188.1
11	Lowest Frequency	-1246.1
12	Nucleus	1H
13	Acquired Size	32768
14	Spectral Size	65536

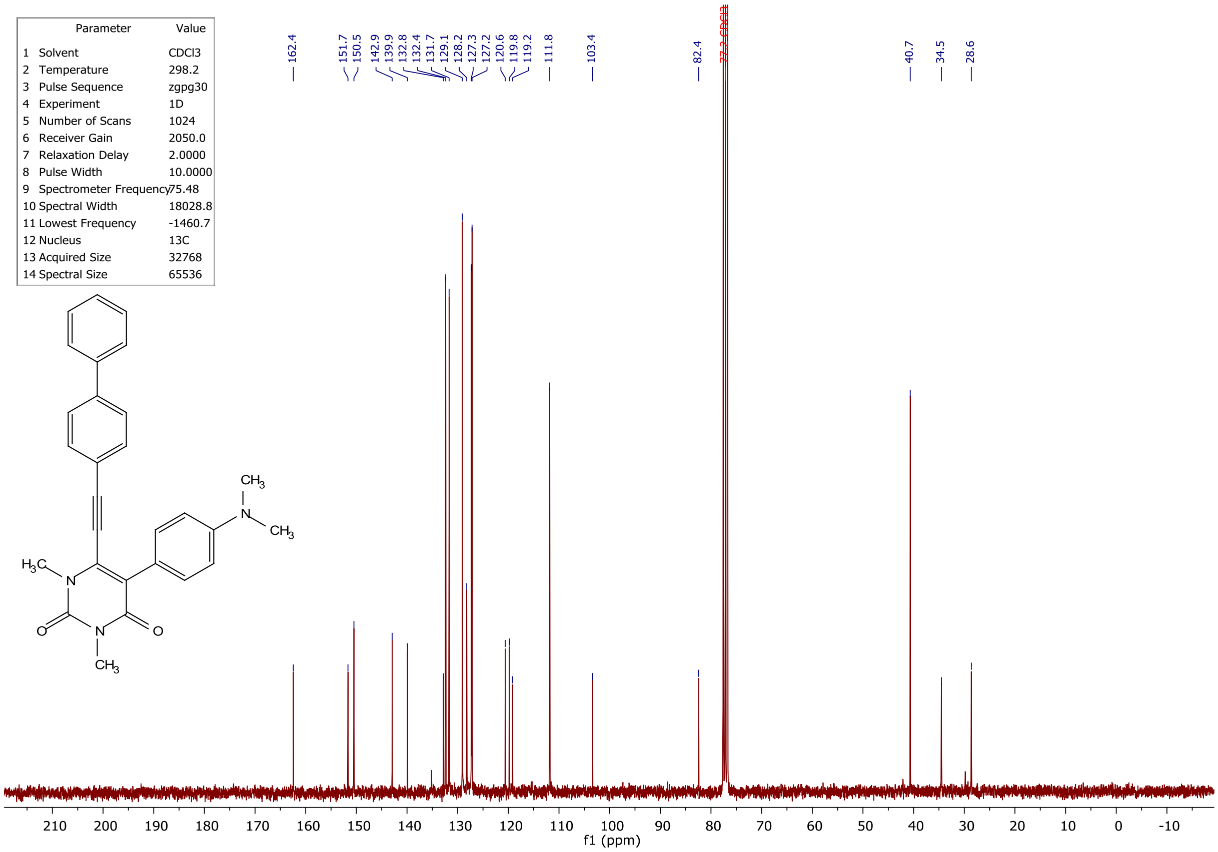

	Parameter	Value
1	Solvent	CDCl3
2	Temperature	298.2
3	Pulse Sequence	zgpg30
4	Experiment	1D
5	Number of Scans	1024
6	Receiver Gain	2050.0
7	Relaxation Delay	2.0000
8	Pulse Width	10.0000
9	Spectrometer Frequency	75.48
10	Spectral Width	18028.8
11	Lowest Frequency	-1460.7
12	Nucleus	13C
13	Acquired Size	32768
14	Spectral Size	65536

1,3-Dimethyl-5-(4-*tert*-butylphenyl)-6-[2-(4-*tert*-butylphenyl)ethynyl]uracil (5j)

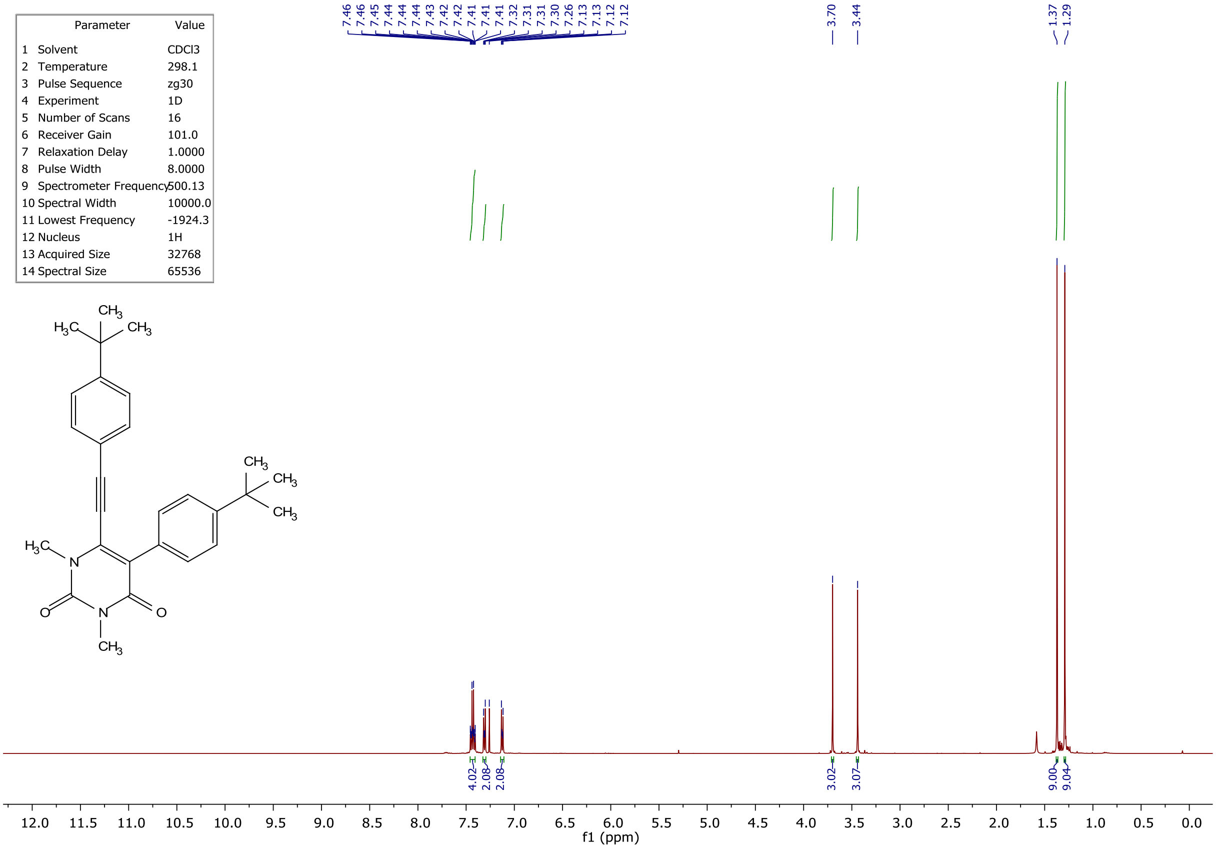

	Parameter	Value
1	Solvent	CDCl3
2	Temperature	298.1
3	Pulse Sequence	zg30
4	Experiment	1D
5	Number of Scans	16
6	Receiver Gain	101.0
7	Relaxation Delay	1.0000
8	Pulse Width	8.0000
9	Spectrometer Frequency	500.13
10	Spectral Width	10000.0
11	Lowest Frequency	-1924.3
12	Nucleus	1H
13	Acquired Size	32768
14	Spectral Size	65536

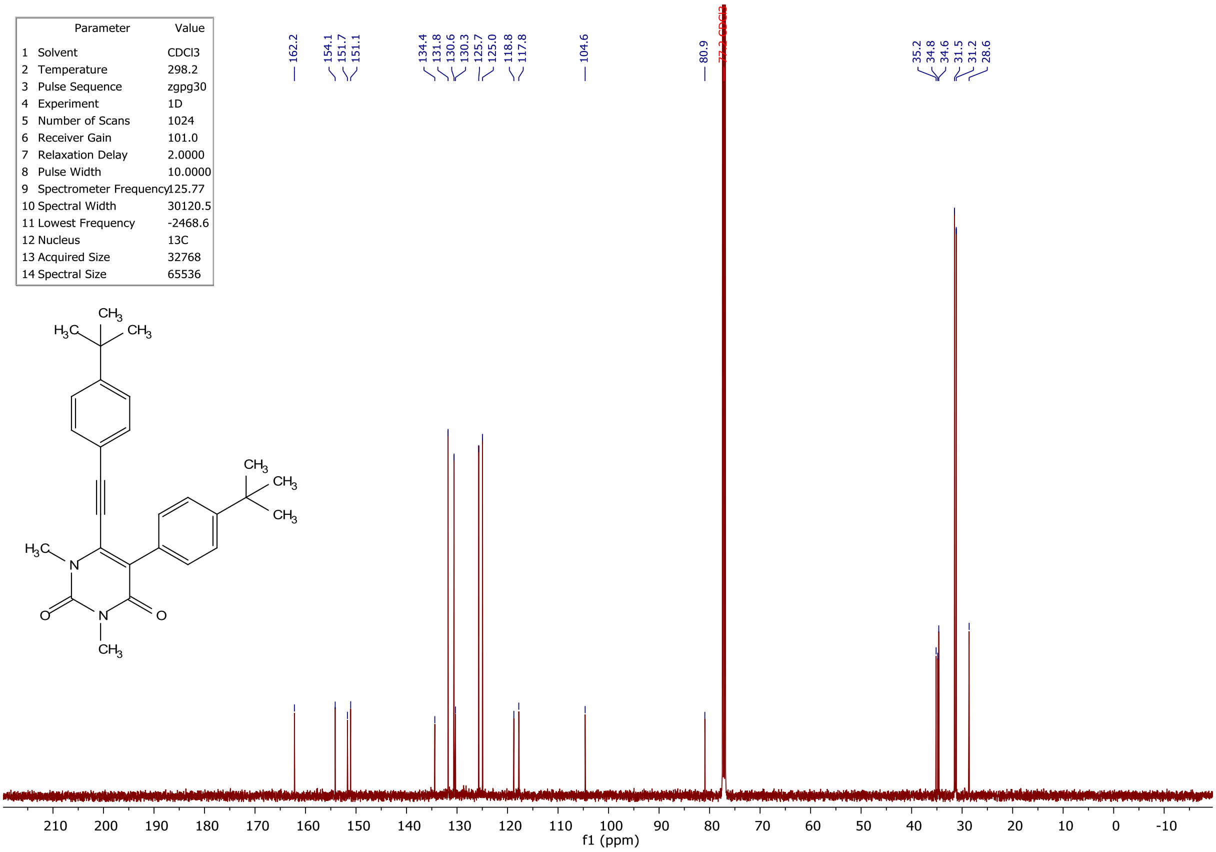

	Parameter	Value
1	Solvent	CDCl3
2	Temperature	298.2
3	Pulse Sequence	zgpg30
4	Experiment	1D
5	Number of Scans	1024
6	Receiver Gain	101.0
7	Relaxation Delay	2.0000
8	Pulse Width	10.0000
9	Spectrometer Frequency	125.77
10	Spectral Width	30120.5
11	Lowest Frequency	-2468.6
12	Nucleus	13C
13	Acquired Size	32768
14	Spectral Size	65536

1,3-Dimethyl-5-(4-methylphenyl)-6-[2-(4-dimethylaminophenyl)ethynyl]uracil (5k)

	Parameter	Value
1	Solvent	CDCl3
2	Temperature	298.2
3	Pulse Sequence	zg30
4	Experiment	1D
5	Number of Scans	16
6	Receiver Gain	203.0
7	Relaxation Delay	2.0000
8	Pulse Width	10.0000
9	Spectrometer Frequency	300.13
10	Spectral Width	6188.1
11	Lowest Frequency	-1247.1
12	Nucleus	1H
13	Acquired Size	32768
14	Spectral Size	65536

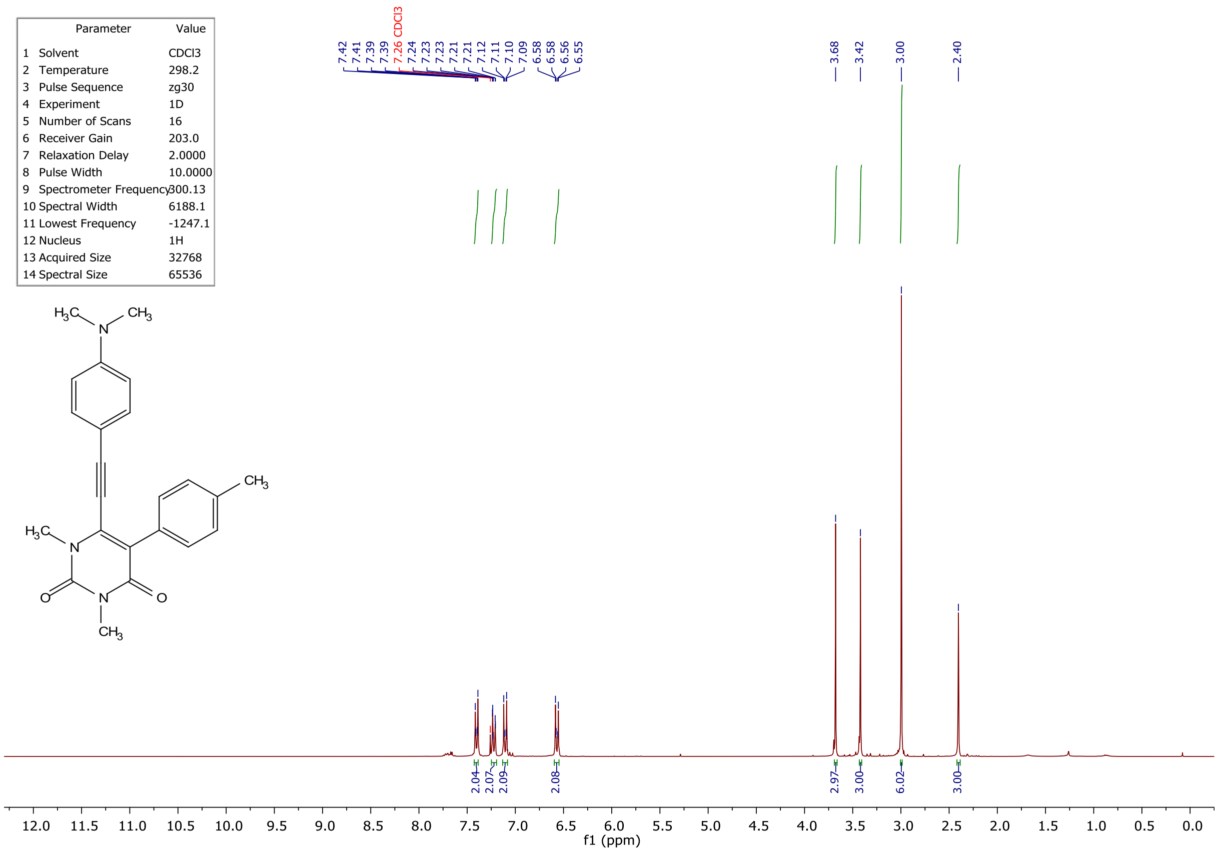

	Parameter	Value
1	Solvent	CDCl3
2	Temperature	298.2
3	Pulse Sequence	zgpg30
4	Experiment	1D
5	Number of Scans	1024
6	Receiver Gain	2050.0
7	Relaxation Delay	2.0000
8	Pulse Width	9.6000
9	Spectrometer Frequency	75.48
10	Spectral Width	18028.8
11	Lowest Frequency	-1460.8
12	Nucleus	13C
13	Acquired Size	32768
14	Spectral Size	65536

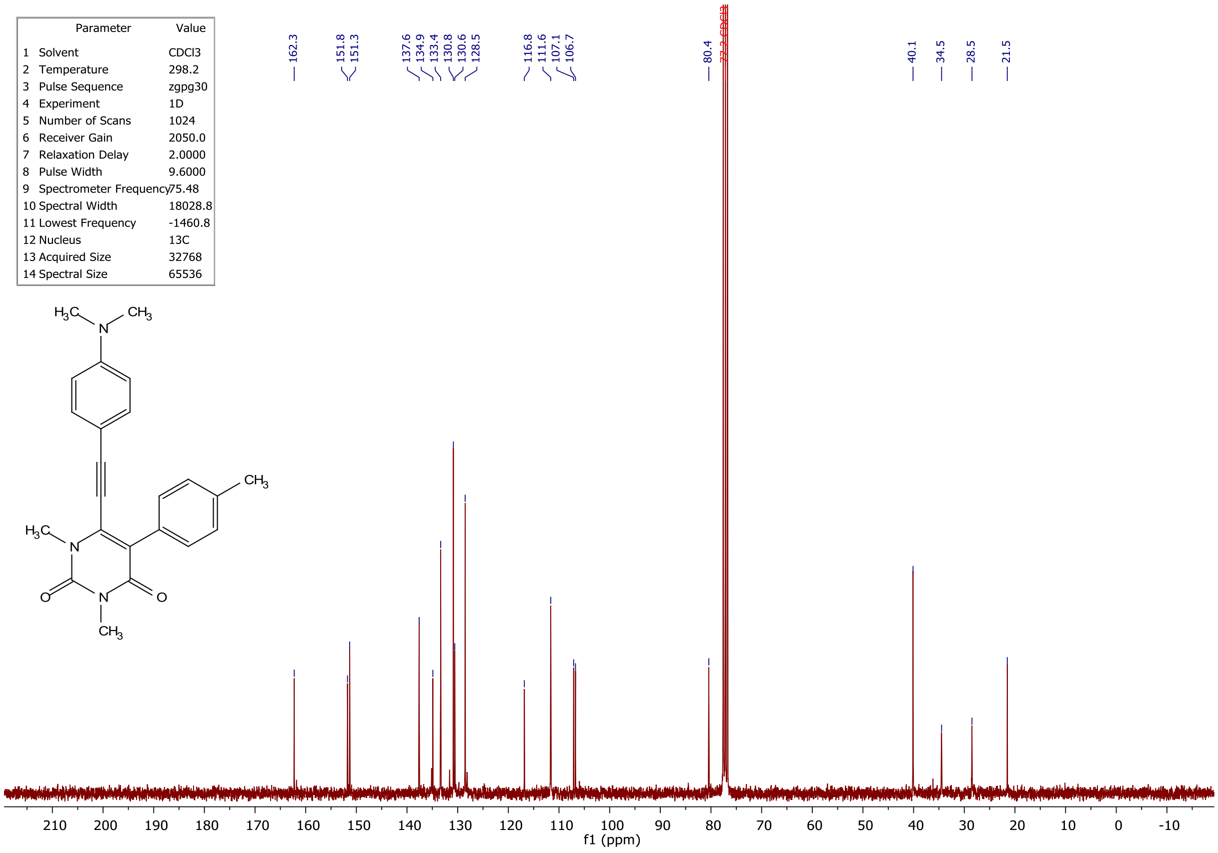

1,3-Dimethyl-5-(4-trifluoromethylphenyl)-6-[2-(4-dimethylaminophenyl)ethynyl]uracil (5l)

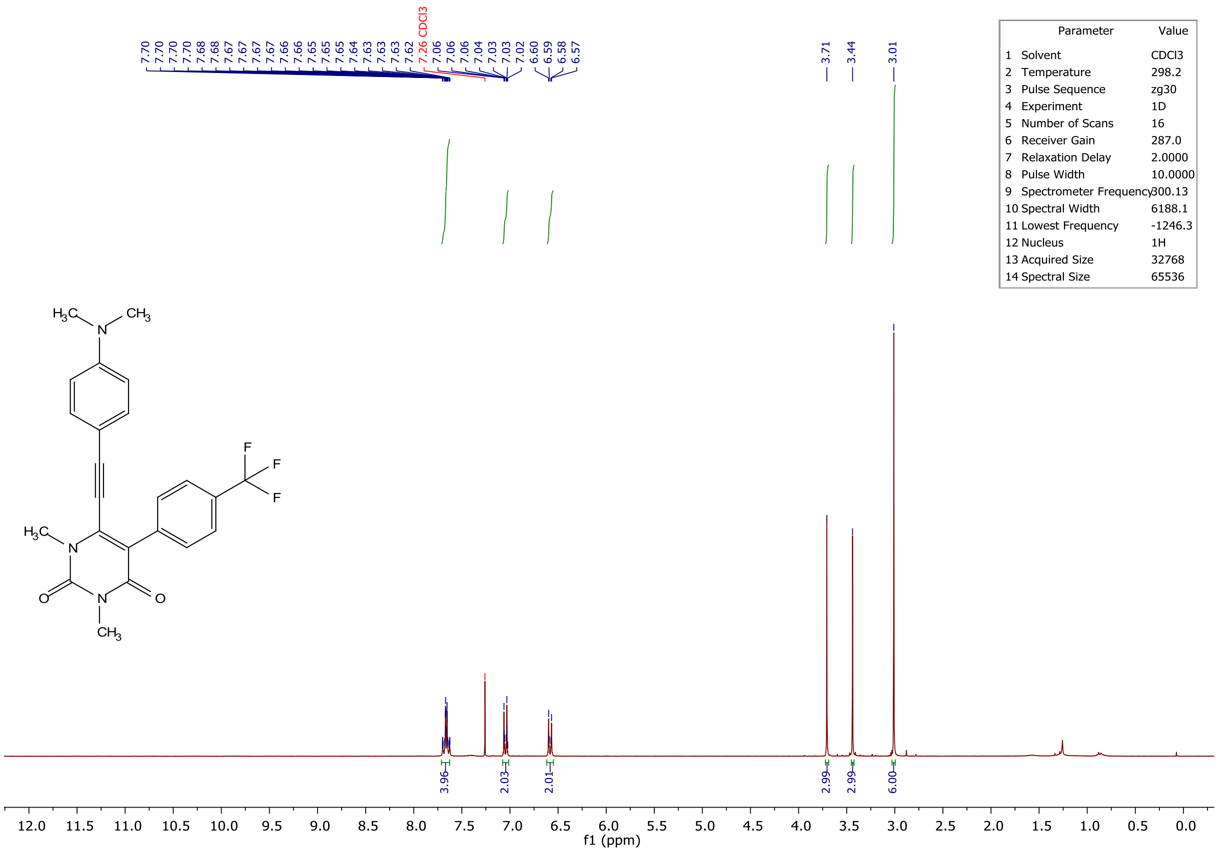

Parameter		Value
1	Solvent	CDCl3
2	Temperature	298.2
3	Pulse Sequence	zg30
4	Experiment	1D
5	Number of Scans	16
6	Receiver Gain	287.0
7	Relaxation Delay	2.0000
8	Pulse Width	10.0000
9	Spectrometer Frequency	300.13
10	Spectral Width	6188.1
11	Lowest Frequency	-1246.3
12	Nucleus	1H
13	Acquired Size	32768
14	Spectral Size	65536

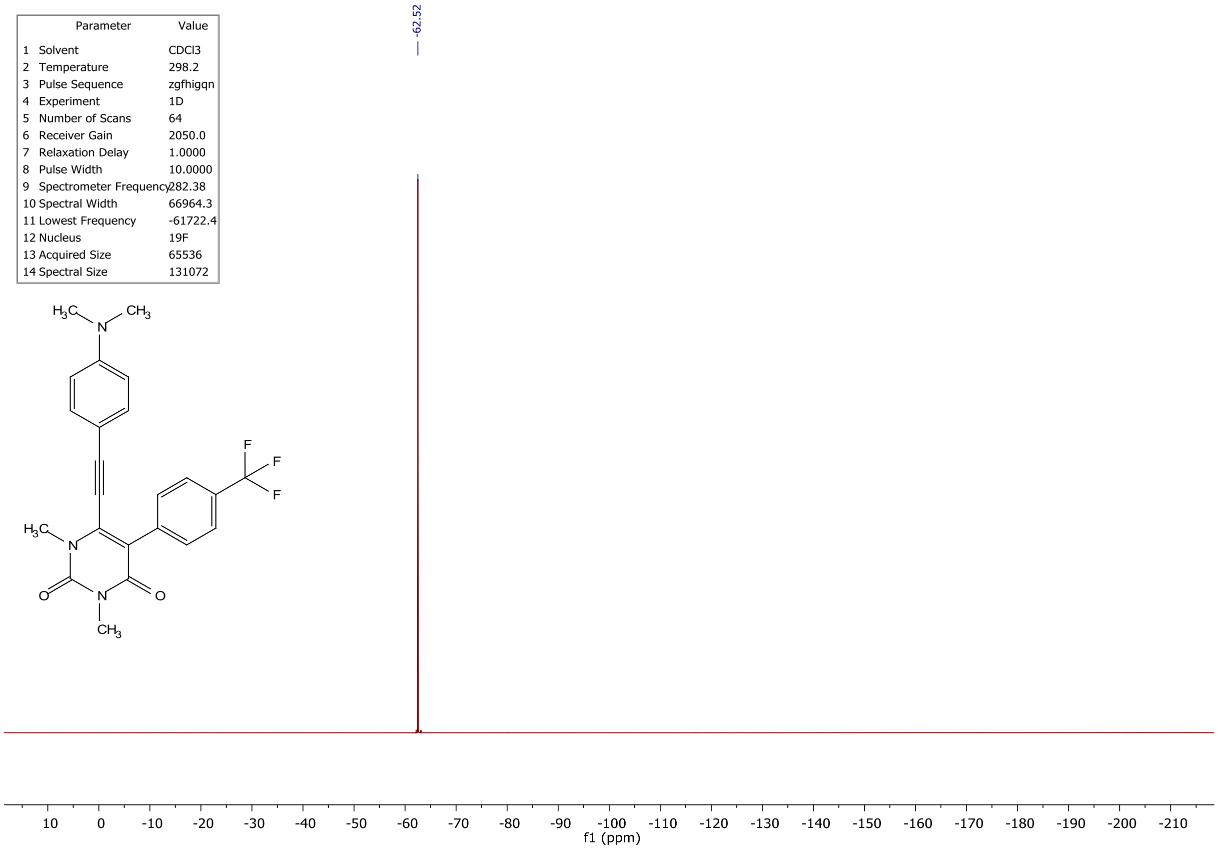

Parameter		Value
1	Solvent	CDCl3
2	Temperature	298.2
3	Pulse Sequence	zgfhigqn
4	Experiment	1D
5	Number of Scans	64
6	Receiver Gain	2050.0
7	Relaxation Delay	1.0000
8	Pulse Width	10.0000
9	Spectrometer Frequency	282.38
10	Spectral Width	66964.3
11	Lowest Frequency	-61722.4
12	Nucleus	19F
13	Acquired Size	65536
14	Spectral Size	131072

	Parameter	Value
1	Solvent	CDCl3
2	Temperature	298.2
3	Pulse Sequence	zgpg30
4	Experiment	1D
5	Number of Scans	5120
6	Receiver Gain	2050.0
7	Relaxation Delay	2.0000
8	Pulse Width	10.0000
9	Spectrometer Frequency	62.90
10	Spectral Width	15000.0
11	Lowest Frequency	-1201.0
12	Nucleus	13C
13	Acquired Size	32768
14	Spectral Size	65536

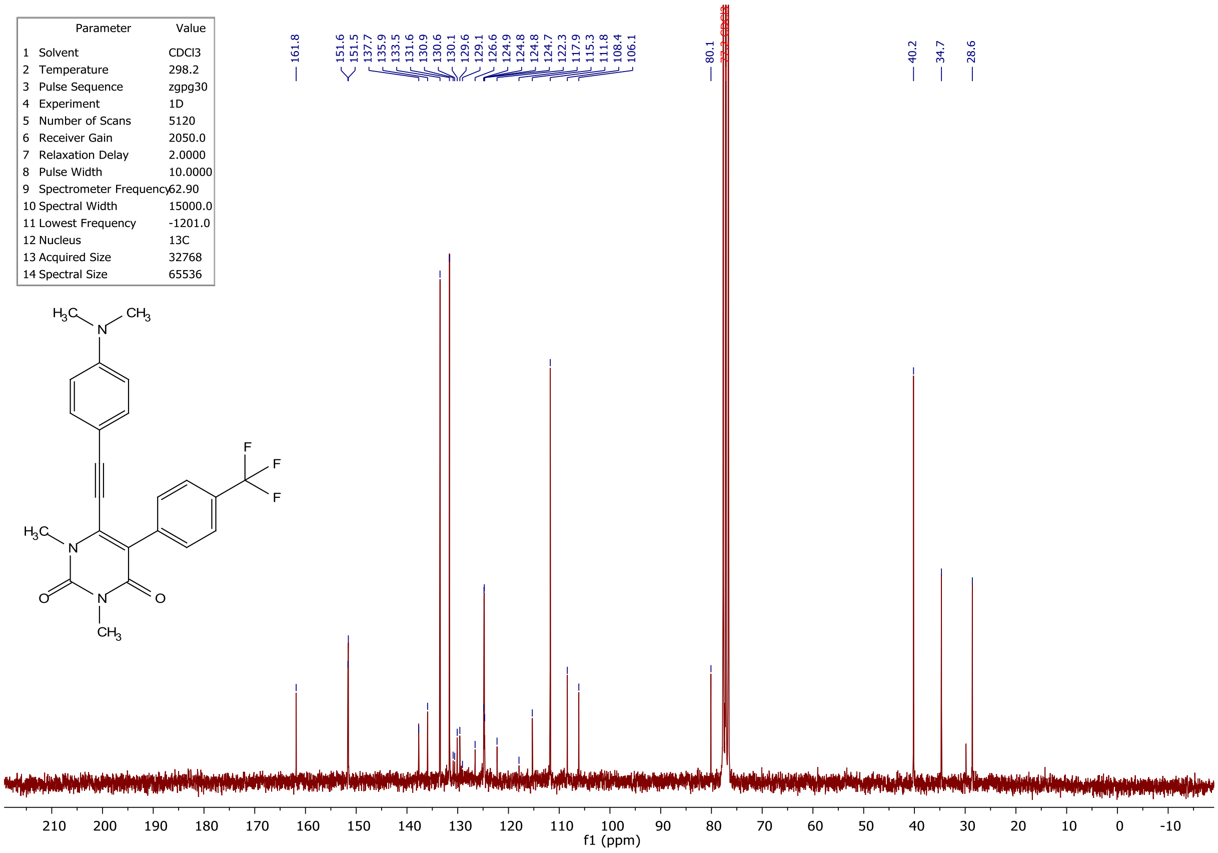

1,3-Dimethyl-5-(4-dimethylaminophenyl)-6-[2-(4-dimethylaminophenyl)ethynyl]uracil (5m)

	Parameter	Value
1	Solvent	CDCl3
2	Temperature	298.2
3	Pulse Sequence	zg30
4	Experiment	1D
5	Number of Scans	16
6	Receiver Gain	101.0
7	Relaxation Delay	1.0000
8	Pulse Width	8.0000
9	Spectrometer Frequency	500.13
10	Spectral Width	10000.0
11	Lowest Frequency	-1924.9
12	Nucleus	1H
13	Acquired Size	32768
14	Spectral Size	65536

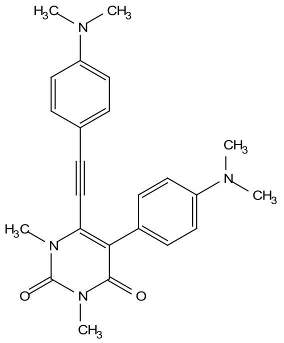

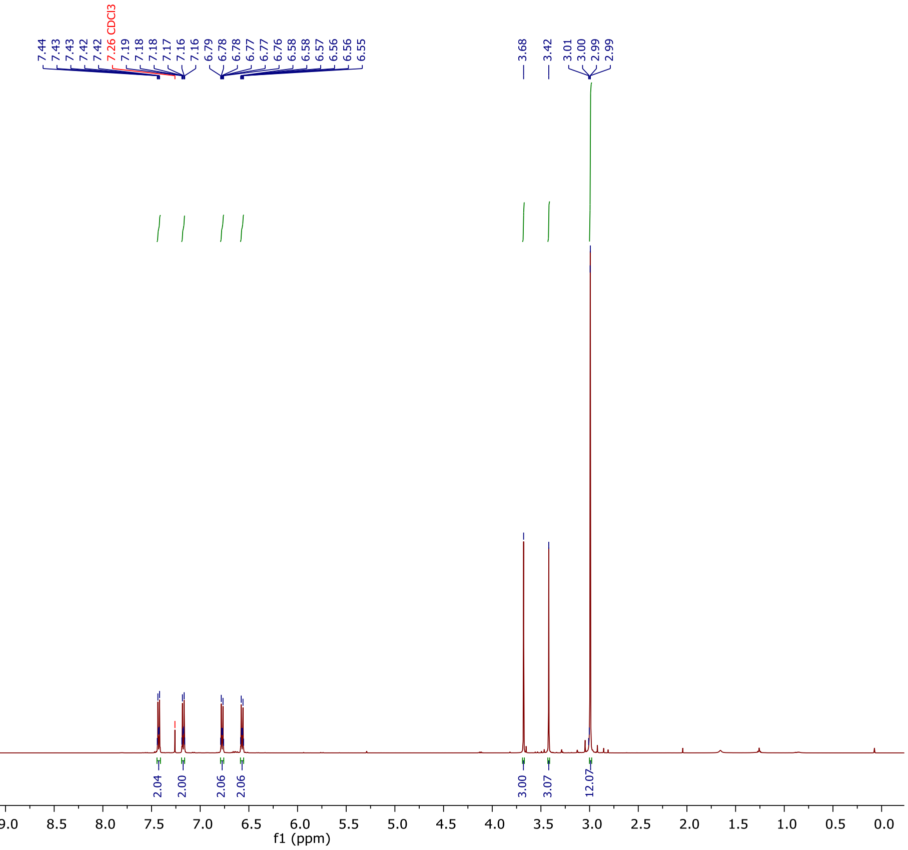

	Parameter	Value
1	Solvent	CDCl3
2	Temperature	298.1
3	Pulse Sequence	zgpg30
4	Experiment	1D
5	Number of Scans	1024
6	Receiver Gain	101.0
7	Relaxation Delay	2.0000
8	Pulse Width	10.0000
9	Spectrometer Frequency	125.77
10	Spectral Width	30120.5
11	Lowest Frequency	-2472.3
12	Nucleus	13C
13	Acquired Size	32768
14	Spectral Size	65536

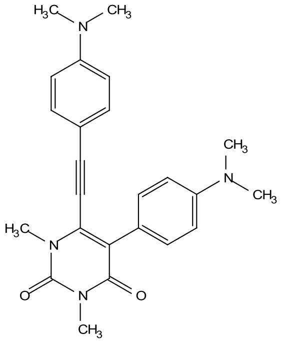

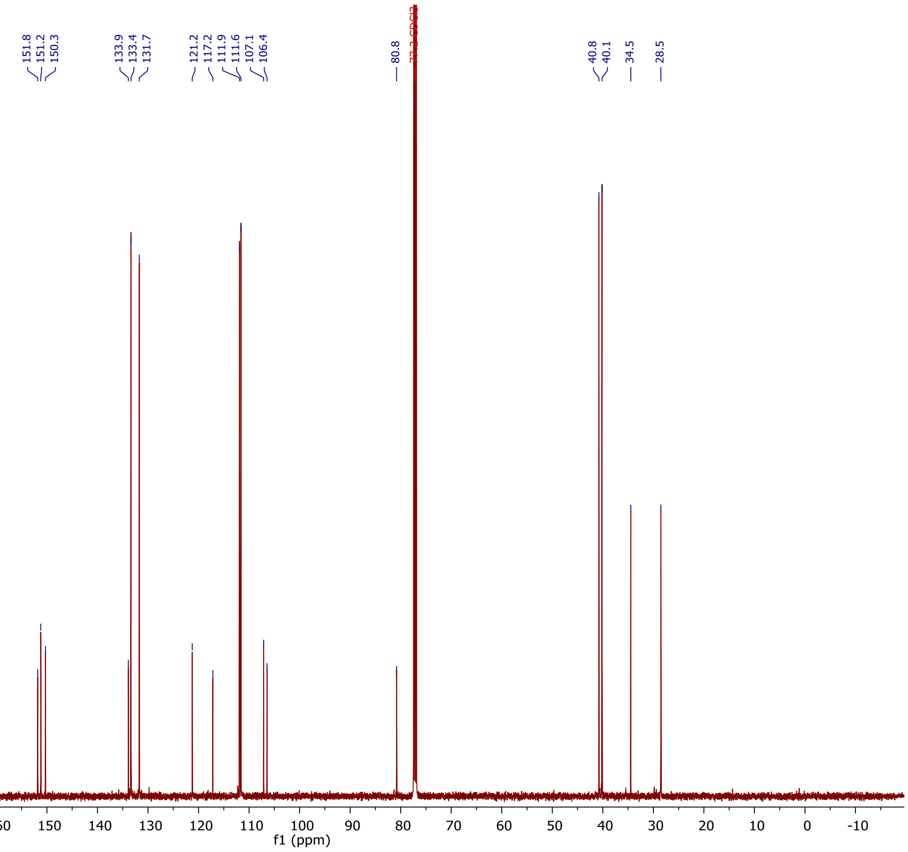

1,3-Dimethyl-6-[2-(4-fluorophenyl)ethynyl]-5-(4-dimethylaminophenyl)uracil (5p)

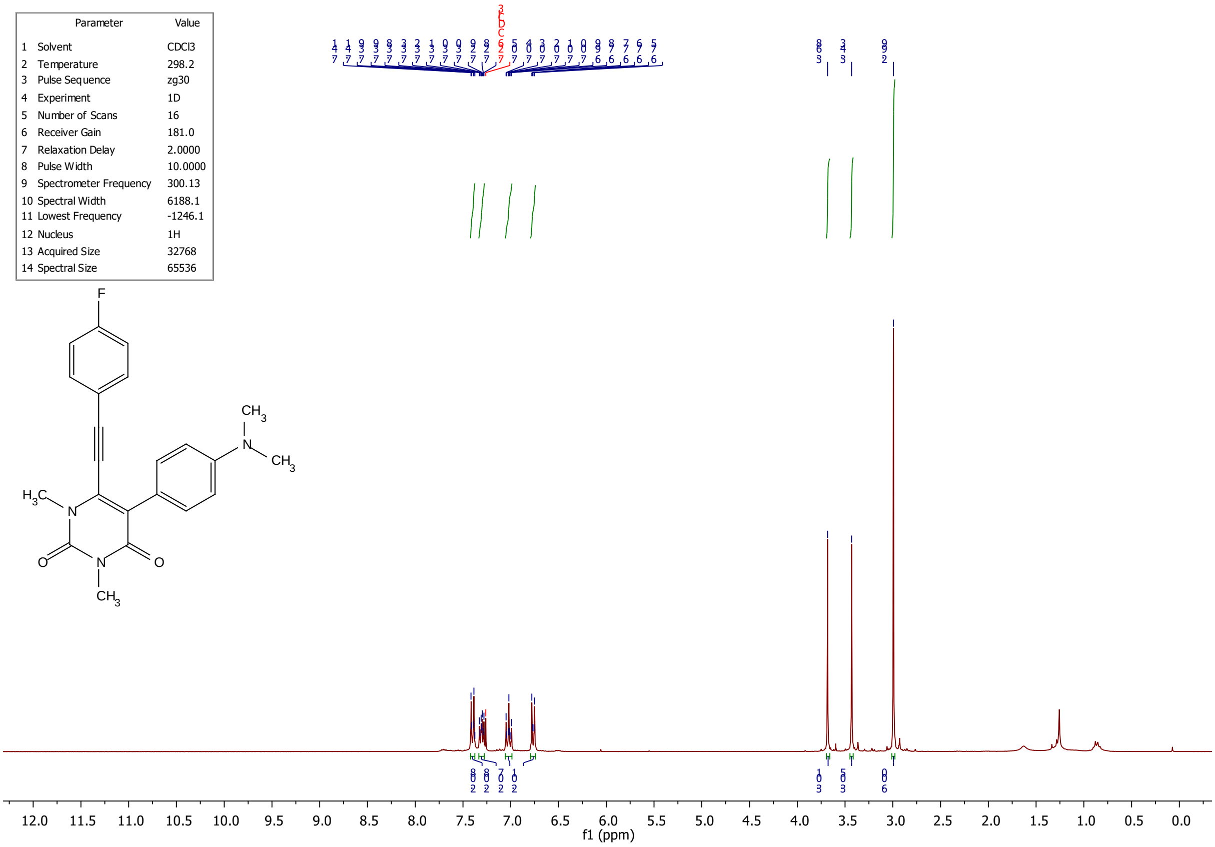

	Parameter	Value
1	Solvent	CDCl3
2	Temperature	298.2
3	Pulse Sequence	zg30
4	Experiment	1D
5	Number of Scans	16
6	Receiver Gain	181.0
7	Relaxation Delay	2.0000
8	Pulse Width	10.0000
9	Spectrometer Frequency	300.13
10	Spectral Width	6188.1
11	Lowest Frequency	-1246.1
12	Nucleus	1H
13	Acquired Size	32768
14	Spectral Size	65536

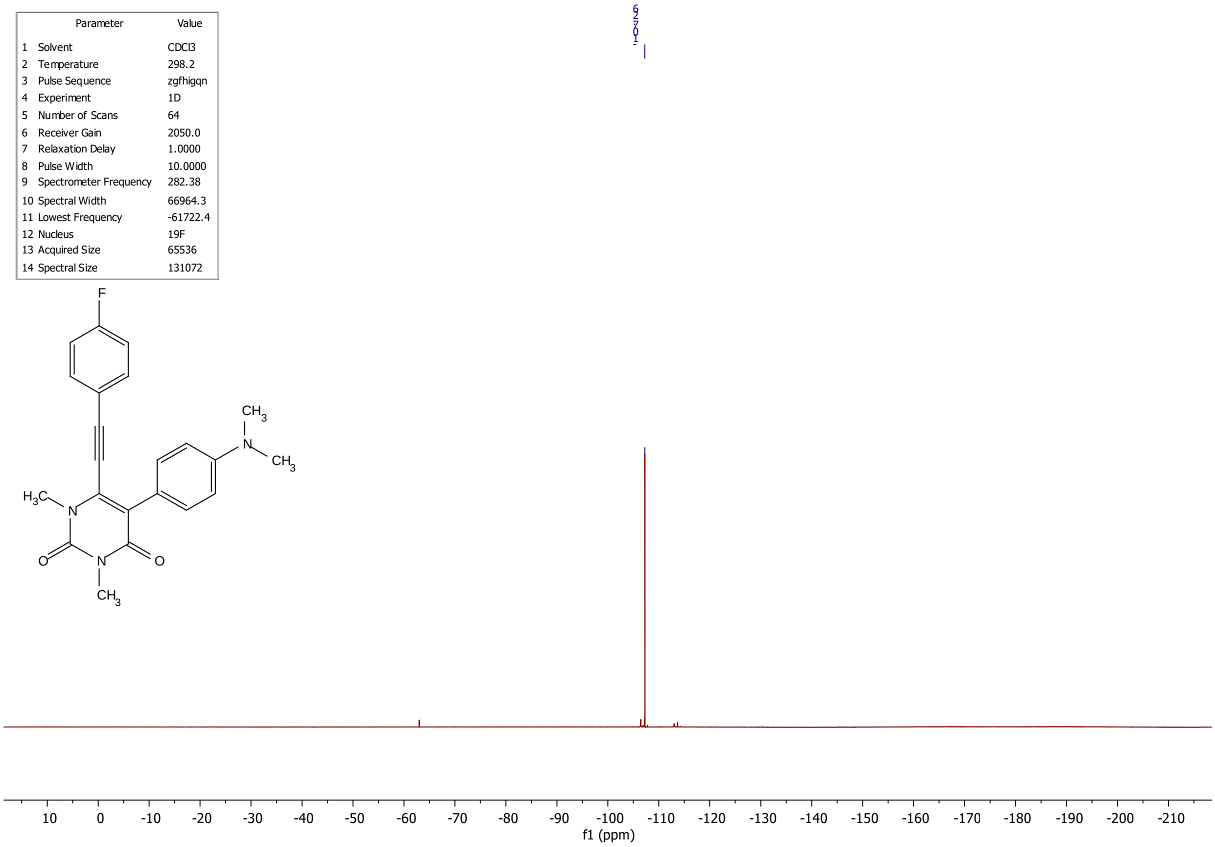

	Parameter	Value
1	Solvent	CDCl3
2	Temperature	298.2
3	Pulse Sequence	zgfhigqn
4	Experiment	1D
5	Number of Scans	64
6	Receiver Gain	2050.0
7	Relaxation Delay	1.0000
8	Pulse Width	10.0000
9	Spectrometer Frequency	282.38
10	Spectral Width	66964.3
11	Lowest Frequency	-61722.4
12	Nucleus	19F
13	Acquired Size	65536
14	Spectral Size	131072

	Parameter	Value
1	Solvent	CDCl3
2	Temperature	298.2
3	Pulse Sequence	zgpg30
4	Experiment	1D
5	Number of Scans	1024
6	Receiver Gain	2050.0
7	Relaxation Delay	2.0000
8	Pulse Width	10.0000
9	Spectrometer Frequency	75.48
10	Spectral Width	18028.8
11	Lowest Frequency	-1468.1
12	Nucleus	13C
13	Acquired Size	32768
14	Spectral Size	65536

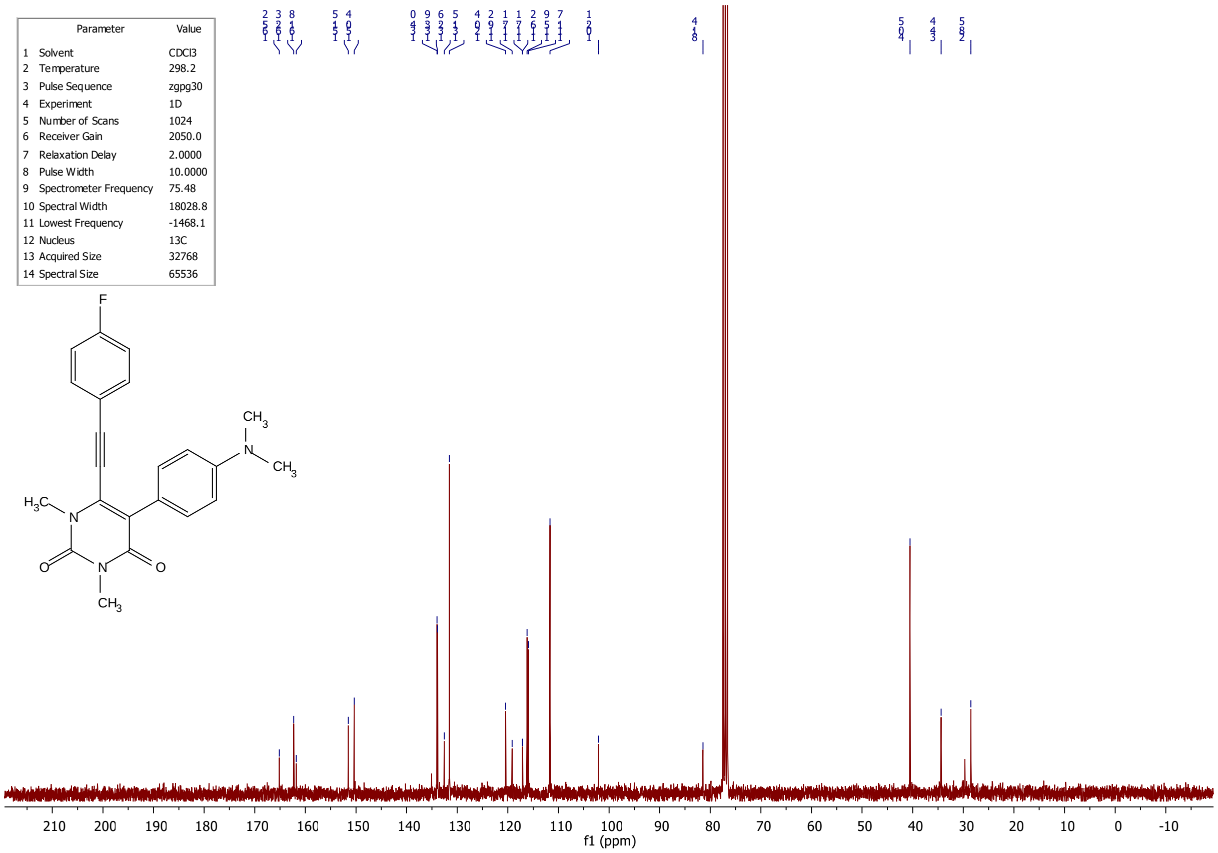

1,3-Dimethyl-6-[2-(4-trifluoromethylphenyl)ethynyl]-5-(4-dimethylaminophenyl)uracil (5q)

	Parameter	Value
1	Solvent	CDCl3
2	Temperature	298.1
3	Pulse Sequence	zg30
4	Experiment	1D
5	Number of Scans	16
6	Receiver Gain	101.0
7	Relaxation Delay	1.0000
8	Pulse Width	8.0000
9	Spectrometer Frequency	500.13
10	Spectral Width	10000.0
11	Lowest Frequency	-1925.2
12	Nucleus	1H
13	Acquired Size	32768
14	Spectral Size	65536

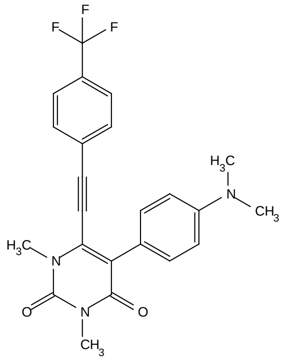

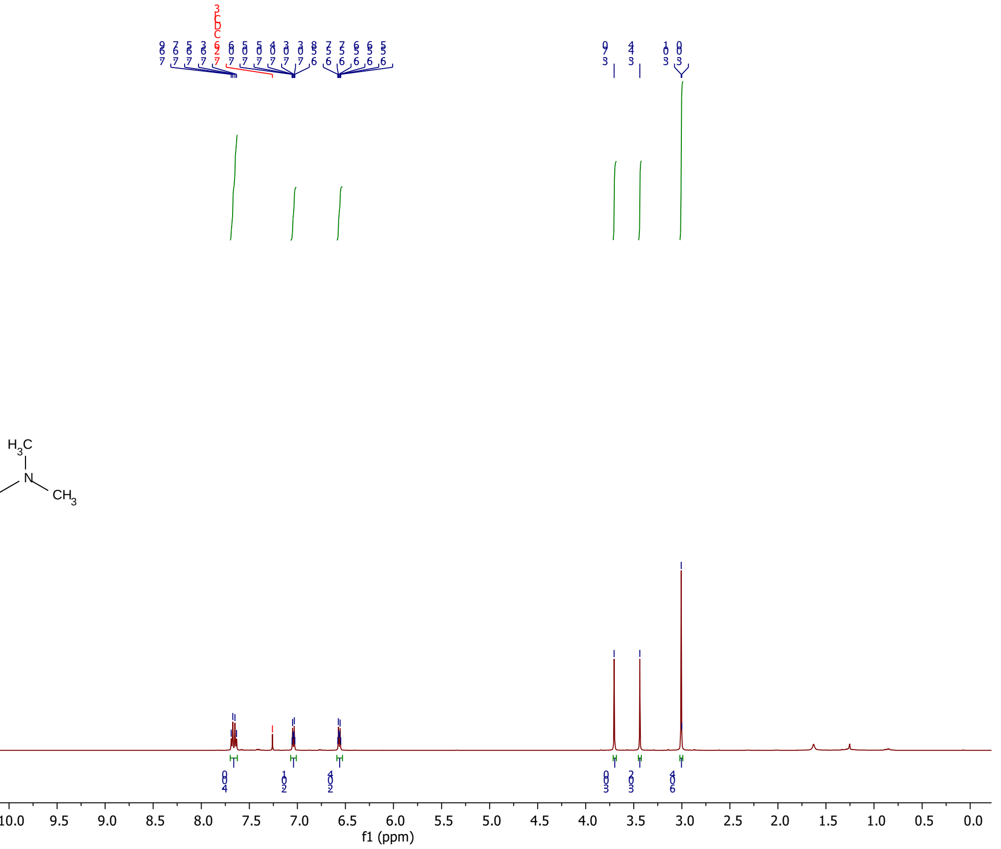

	Parameter	Value
1	Solvent	CDCl3
2	Temperature	298.2
3	Pulse Sequence	zgig
4	Experiment	1D
5	Number of Scans	16
6	Receiver Gain	101.0
7	Relaxation Delay	1.0000
8	Pulse Width	12.0000
9	Spectrometer Frequency	470.55
10	Spectral Width	113636.4
11	Lowest Frequency	-103877.4
12	Nucleus	19F
13	Acquired Size	65536
14	Spectral Size	131072

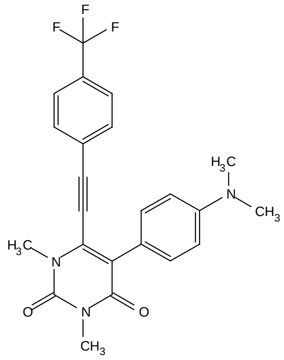

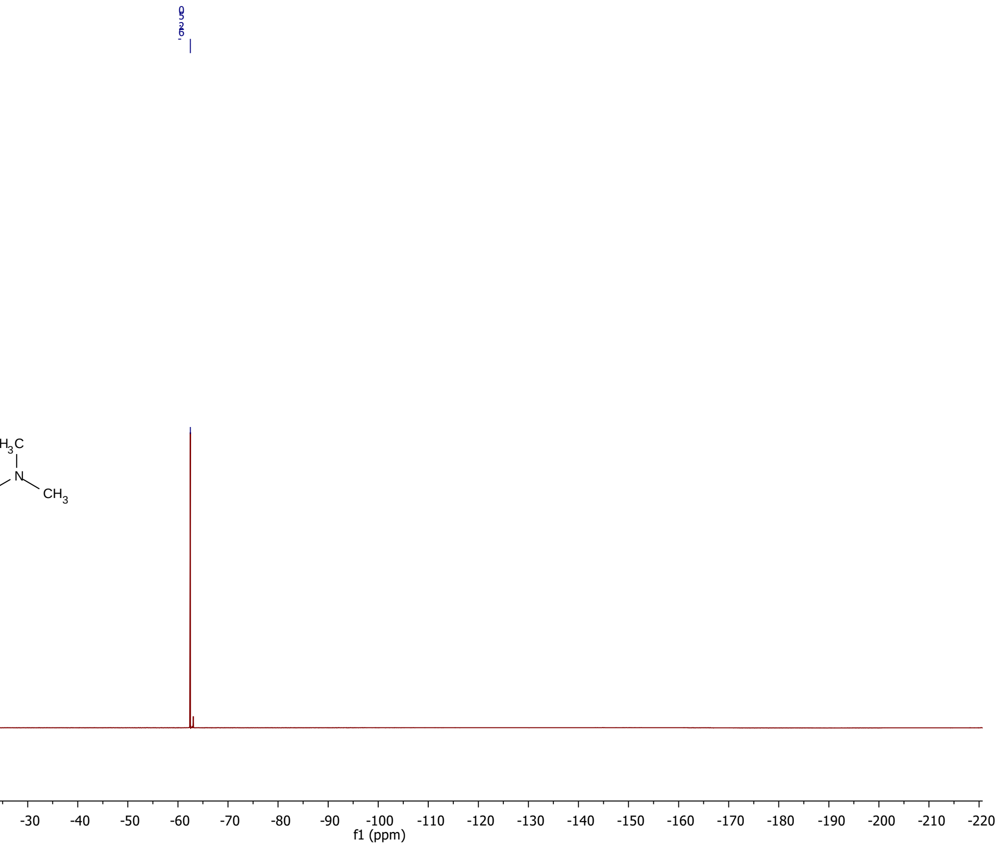

	Parameter	Value
1	Solvent	CDCl3
2	Temperature	298.1
3	Pulse Sequence	zgpg30
4	Experiment	1D
5	Number of Scans	4096
6	Receiver Gain	101.0
7	Relaxation Delay	2.0000
8	Pulse Width	10.0000
9	Spectrometer Frequency	125.77
10	Spectral Width	30120.5
11	Lowest Frequency	-2468.9
12	Nucleus	13C
13	Acquired Size	32768
14	Spectral Size	65536

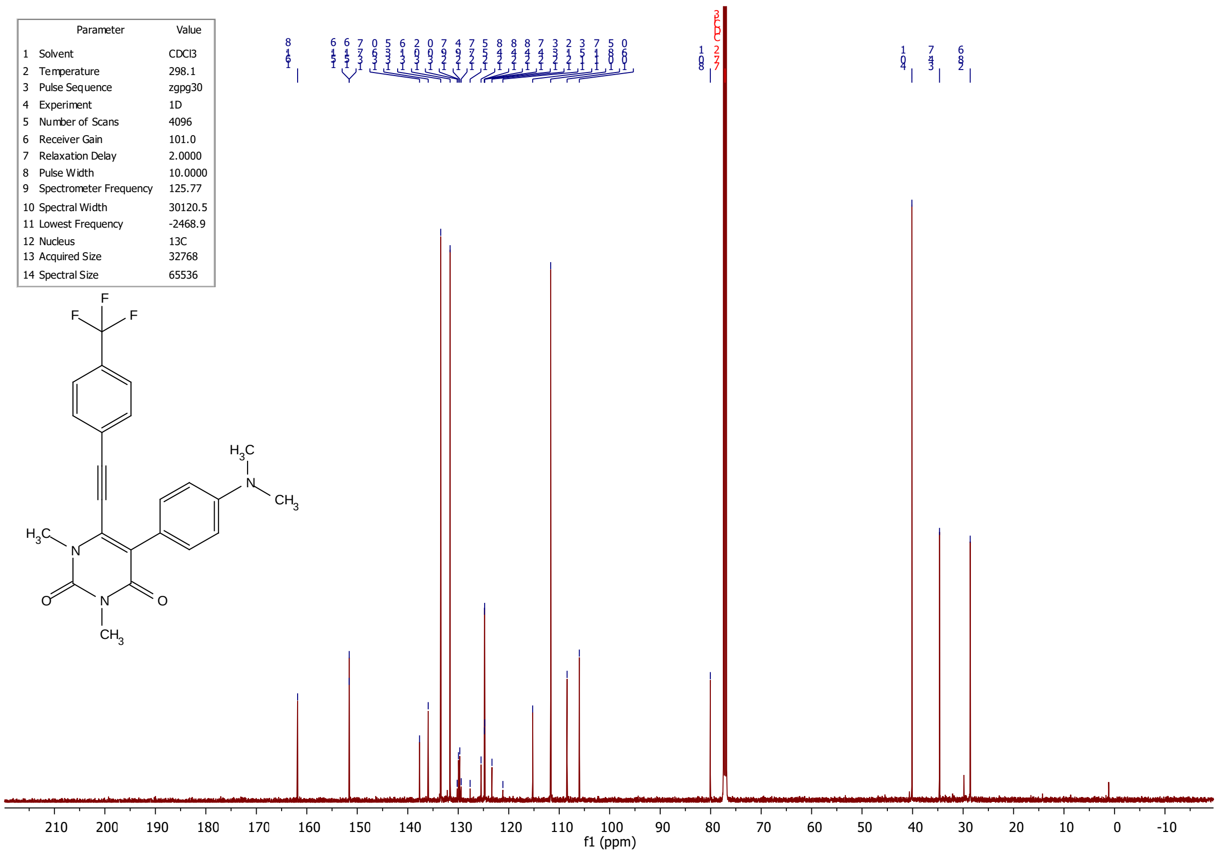

6-((4-(Dimethylamino)phenyl)ethynyl)-1,3-dimethyl-5-(thiophen-3-yl)pyrimidine-2,4(1*H*,3*H*)-dione (5r)

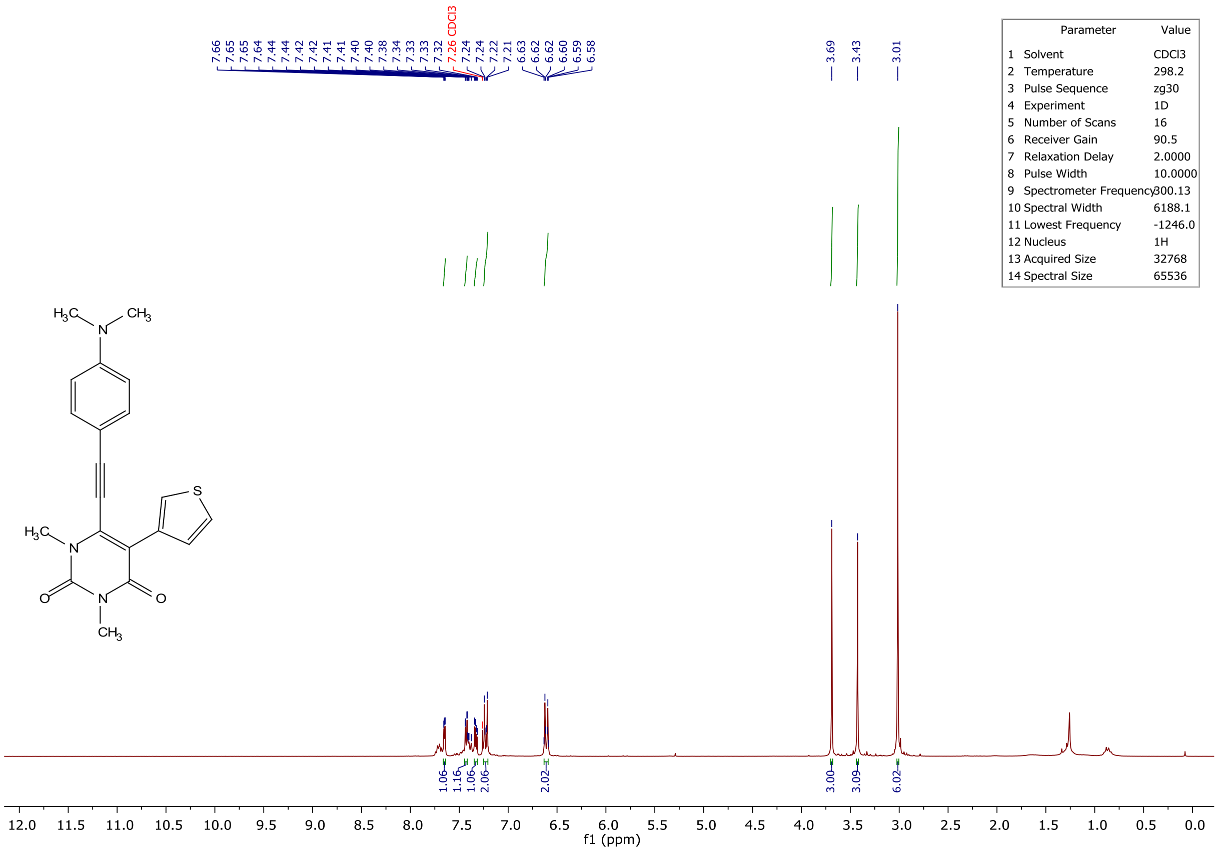

	Parameter	Value
1	Solvent	CDCl3
2	Temperature	298.2
3	Pulse Sequence	zg30
4	Experiment	1D
5	Number of Scans	16
6	Receiver Gain	90.5
7	Relaxation Delay	2.0000
8	Pulse Width	10.0000
9	Spectrometer Frequency	300.13
10	Spectral Width	6188.1
11	Lowest Frequency	-1246.0
12	Nucleus	1H
13	Acquired Size	32768
14	Spectral Size	65536

	Parameter	Value
1	Solvent	CDCl3
2	Temperature	298.2
3	Pulse Sequence	zgpg30
4	Experiment	1D
5	Number of Scans	1024
6	Receiver Gain	2050.0
7	Relaxation Delay	2.0000
8	Pulse Width	10.0000
9	Spectrometer Frequency	75.48
10	Spectral Width	18028.8
11	Lowest Frequency	-1460.0
12	Nucleus	13C
13	Acquired Size	32768
14	Spectral Size	65536

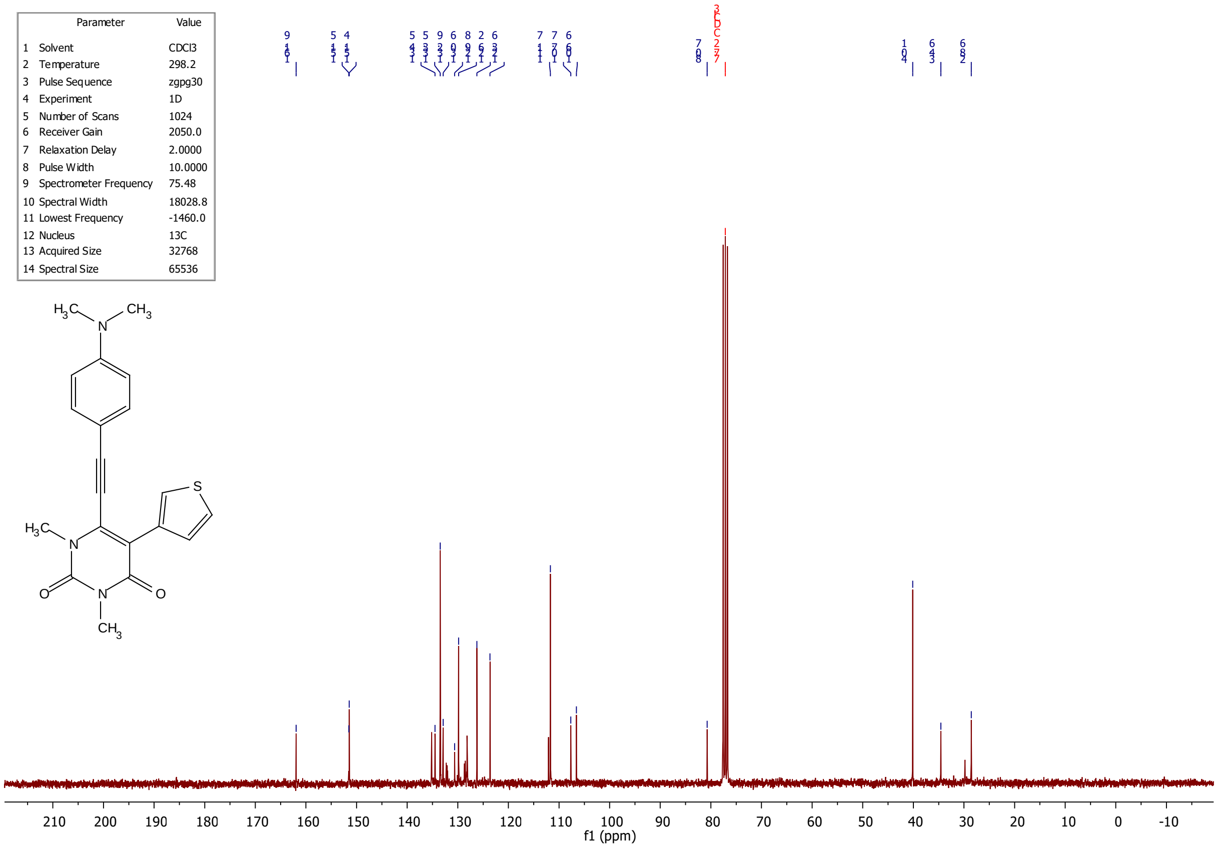

5-(4-(Dimethylamino)phenyl)-1,3-dimethyl-6-(thiophen-3-ylethynyl)pyrimidine-2,4(1*H*,3*H*)-dione (5s)

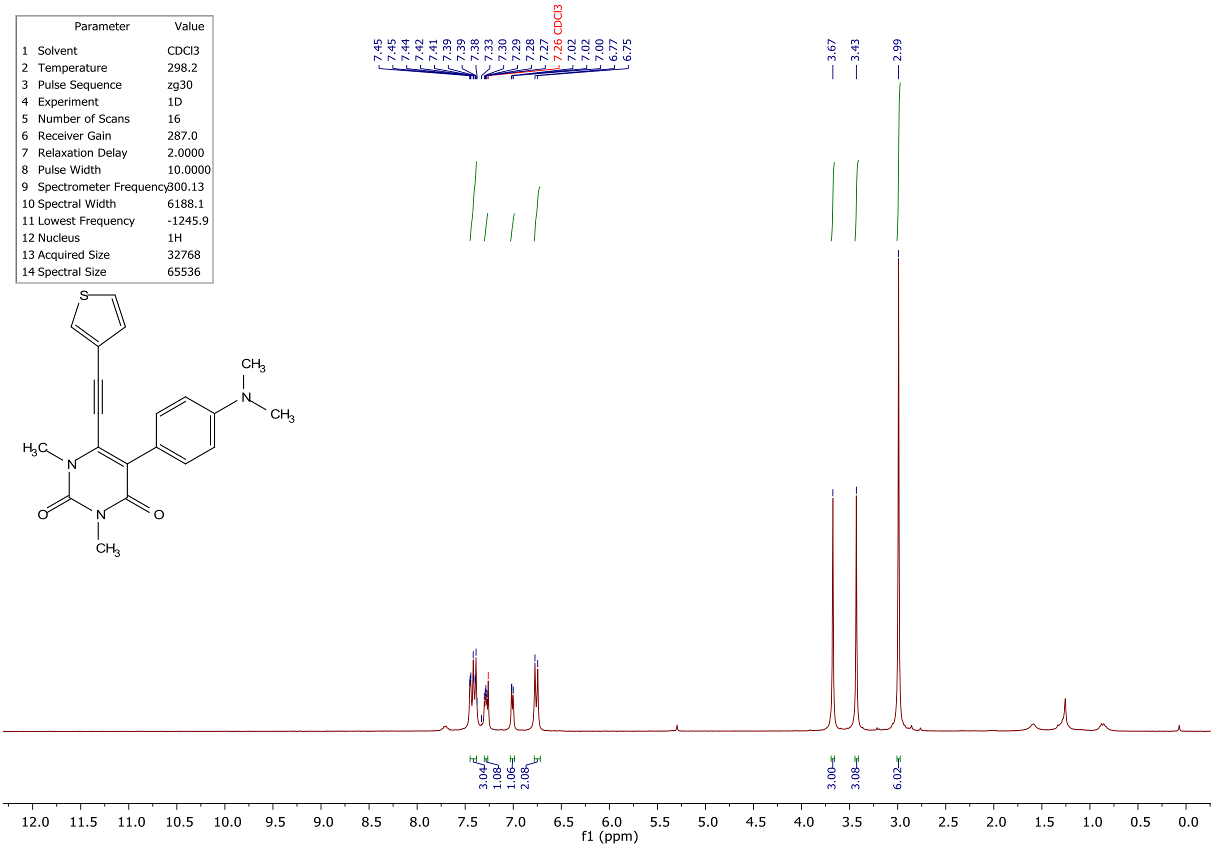

Parameter		Value
1	Solvent	CDCl3
2	Temperature	298.2
3	Pulse Sequence	zg30
4	Experiment	1D
5	Number of Scans	16
6	Receiver Gain	287.0
7	Relaxation Delay	2.0000
8	Pulse Width	10.0000
9	Spectrometer Frequency	300.13
10	Spectral Width	6188.1
11	Lowest Frequency	-1245.9
12	Nucleus	1H
13	Acquired Size	32768
14	Spectral Size	65536

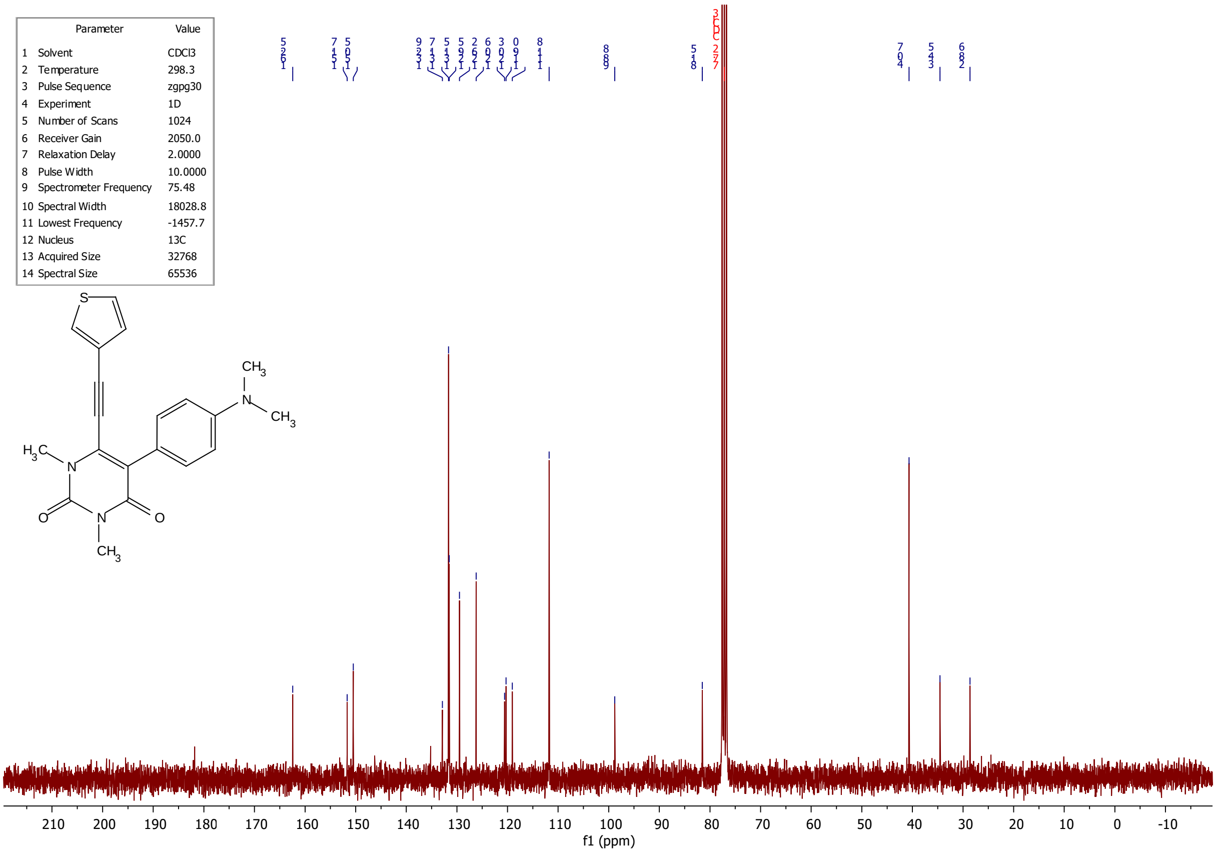

Parameter		Value
1	Solvent	CDCl3
2	Temperature	298.3
3	Pulse Sequence	zgpg30
4	Experiment	1D
5	Number of Scans	1024
6	Receiver Gain	2050.0
7	Relaxation Delay	2.0000
8	Pulse Width	10.0000
9	Spectrometer Frequency	75.48
10	Spectral Width	18028.8
11	Lowest Frequency	-1457.7
12	Nucleus	13C
13	Acquired Size	32768
14	Spectral Size	65536

5-(Furan-3-yl)-1,3-dimethyl-6-(*p*-tolylethynyl)pyrimidine-2,4(1*H*,3*H*)-dione (5t)

	Parameter	Value
1	Solvent	CDCl3
2	Temperature	298.2
3	Pulse Sequence	zg30
4	Experiment	1D
5	Number of Scans	16
6	Receiver Gain	203.0
7	Relaxation Delay	2.0000
8	Pulse Width	10.0000
9	Spectrometer Frequency	300.13
10	Spectral Width	6188.1
11	Lowest Frequency	-1246.4
12	Nucleus	1H
13	Acquired Size	32768
14	Spectral Size	65536

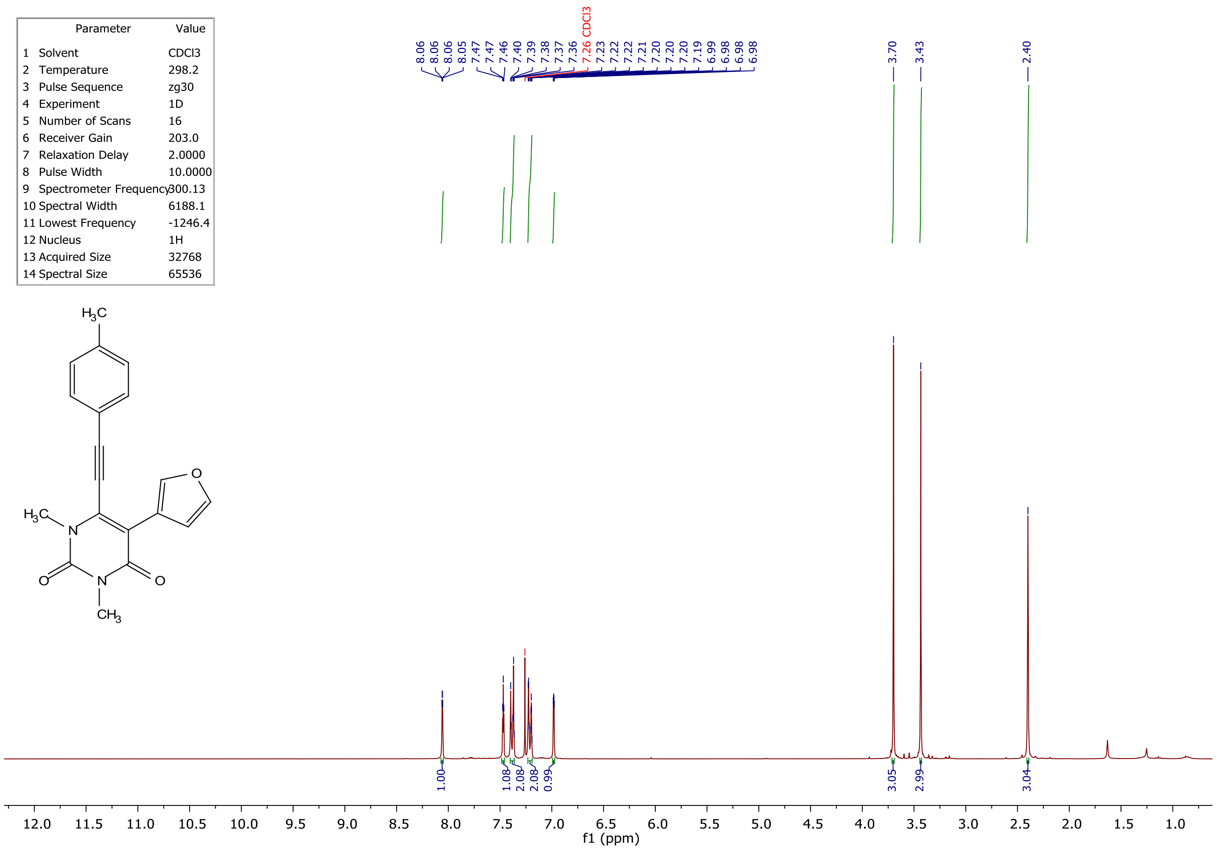

	Parameter	Value
1	Solvent	CDCl3
2	Temperature	298.4
3	Pulse Sequence	zgpg30
4	Experiment	1D
5	Number of Scans	1024
6	Receiver Gain	2050.0
7	Relaxation Delay	2.0000
8	Pulse Width	10.0000
9	Spectrometer Frequency	75.48
10	Spectral Width	18028.8
11	Lowest Frequency	-1458.5
12	Nucleus	13C
13	Acquired Size	32768
14	Spectral Size	65536

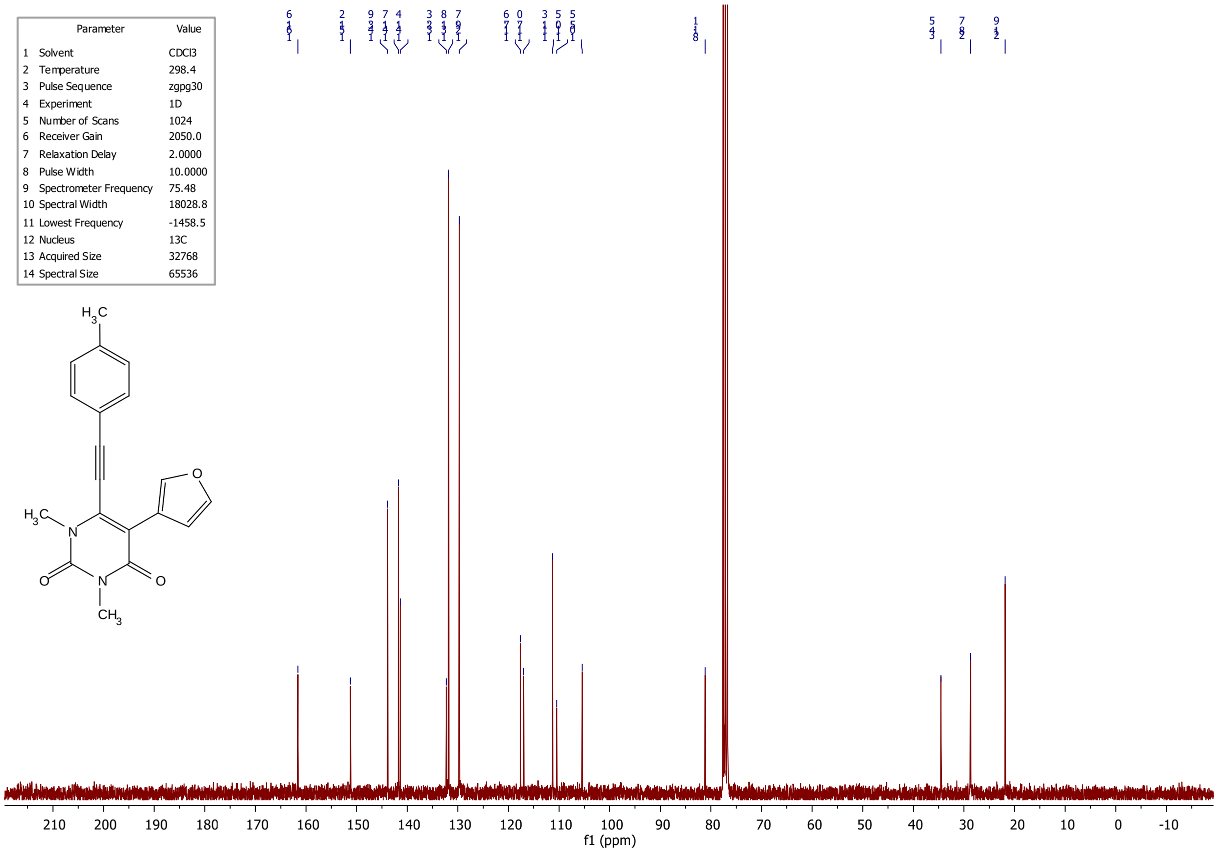

Supplement: File 1 — Experimental data and copies of spectra. [file Beilstein_J_Org_Chem-20-898-s001.pdf]
